# Diastereoselective Additive Trifluoromethylation/Halogenation of Isoxazole Triflones: Synthesis of All-Carbon-Functionalized Trifluoromethyl Isoxazoline Triflones

**DOI:** 10.1002/open.201300044

**Published:** 2014-02-13

**Authors:** Hiroyuki Kawai, Yutaka Sugita, Etsuko Tokunaga, Hiroyasu Sato, Motoo Shiro, Norio Shibata

**Affiliations:** [a] Department of Frontier Materials, Graduate School of Engineering, Nagoya Institute of TechnologyGokiso, Showa-ku, Nagoya 466-8555 (Japan); [b] Rigaku Corporation, 3-9-12 Mastubara-choAkishima, Tokyo 196-8666 (Japan)

**Keywords:** agrochemicals, halogenations, isoxazolines, trifluoromethanesulfonyl, trifluoromethylations

## Abstract

Highly functionalized 5-trifluoromethyl-2-isoxazoline derivatives featuring a triflyl (SO_2_CF_3_) group at the 4-position were successfully synthesized via diastereoselective trifluoromethylation and halogenation of isoxazole triflones using the Ruppert– Prakash reagent. The trifluoromethylation is quite general in terms of the substrates including 3,5-diaryl isoxazole triflones and 3-aryl-5-styrylisoxazole triflones to provide products in high yields with excellent diastereoselectivities. The highly functionalized 5-trifluoromethyl-2-isoxazoline derivatives are expected to be a new class of antiparasiticides. Thus the triflyl group both activates isoxazoles and the 4-postion of CF_3_ adducts, and has a potential biological function.

Individually, heterocycles and fluorinated compounds have attracted much attention of chemists in pharmaceutical and agrochemical industries over the past few decades. Thus, a search for novel drug candidates based on fluorinated heterocyclic frameworks has become a new dependable strategy in modern medicinal chemistry.[1a] In 2004, 3,5-diaryl-5-(trifluoromethyl)-2-isoxazoline derivatives were disclosed as promising agrochemicals of pest control agents by Nissan chemical industries.[2] This discovery promptly induced many academic and industrial chemists to focus on these small trifluoromethylated heterocycles as important leads of agrochemicals and veterinary medicines.[3a] A large number of trifluoromethylated heterocyclic variants have been designed, including isoxazolines, pyrrolines and pyrazolines with a quaternary carbon bearing a CF_3_ group at the 5-position as a common structural feature (Figure 1).[4]–[6] In this context, we were interested in 4-functionalized 3,5-diaryl-5-(trifluoromethyl)-2-isoxazoline derivatives as novel lead candidates of drug discovery in a future market. The potential of 1-, 2-, 3-and 5-positions at this small ring are well investigated; however, research on the functionalization at the 4-position of this scaffold is rather immature.

**Figure 1:**
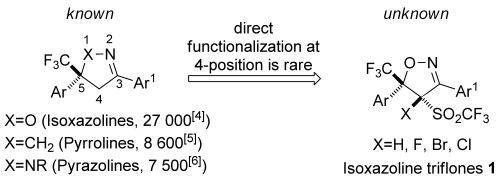
Biologically potent trifluoromethyl isoxazolines, pyrrolines, pyrazolines, and highly functionalized isoxazoline triflones 1 (unknown).

The trifluoromethanesulfonyl (triflyl, SO_2_CF_3_, Tf) group is the strongest electron-withdrawing group (EWG), equivalent to the nitro group (Hammett substituent constants: SO_2_CF_3_, *σ*_p_=0.83, *σ*_m_=0.96; NO_2_, *σ*_p_=0.71, *σ*_m_=0.78), while the lipophilicity of the two is exactly opposite (SO_2_CF_3_, *π*_p_=0.55; NO_2_, *π*_p_=−0.28)[1a],[7a] Thus, introduction of the triflyl group into organic molecules is an effective method to dramatically alter the chemical properties of parent molecules without changing molecular complexities. Indeed, the triflyl group has been successfully used in the fields of chiral catalysts,[8a] pharmaceuticals,[9a] and advanced functional materials.[10a] As part of our research programs directed at the design and synthesis of biologically attractive heteroaryl triflones[11a] and diaryl-5-(trifluoromethyl)-2-isoxazoline derivatives,[12a] we were fascinated by unknown 4-functionalized triflones derivatives **1**. However, functionalization at the 4-position using a well-studied building block strategy is rare.[2]–[6] In 2011, we reported trifluoromethylation at the 5-position of isoxazoles by nucleophilic addition using the Ruppert–Prakash reagent, (trifluoromethyl)trimethylsilane (Me_3_SiCF_3_).[12b] The key for this transformation is the activation of nonreactive aromatic isoxazoles with a nitro group at the 4-position, which achieved the first trifluoromethylation of aromatic isoxazoles. In contrast to the nitro group, we envisioned that the triflyl group should (1) activate aromatic isoxazoles, (2) promote additional functionalization at the 4-position, and (3) have a potential biological function. We disclose herein the synthesis of 5-trifluoromethyl-4-triflyl-2-isoxazolines **1** (X=H, Figure 1, Scheme 1) by direct additive trifluoromethylation of isoxazole triflones **2** with Me_3_SiCF_3_ in high yields and with high diastereoselectivities. The resulting isoxazoline triflones were efficiently halogenated under natural conditions to provide all-carbon functionalized isoxazoline triflones **1** (X=F, Cl, Br) in excellent diastereoselectivities individually, or in a one-pot sequential procedure from isoxazole triflones **2**. The triflyl analogue of antiparasiticide **1 p** was prepared by this method. Cinnamyl-substituted isoxazoline triflone **1 q** was also synthesized regioselectively under the same reaction condition (Scheme 1).

**Scheme 1:**
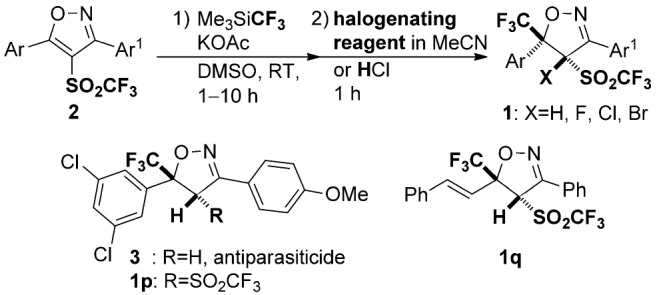
Synthesis of highly functionalized trifluoromethyl isoxazoline triflones 1.

We first examined the reaction of 3,5-diphenyl-4-(trifluoromethanesulfonyl)isoxazole (**2 a**) as a model substrate under the same reaction condition employed for the previously reported additive trifluoromethylation of 4-nitroisoxazoles,[12b] namely, with Me_3_SiCF_3_ using NaOAc in *N*,*N*-dimethylformamide (DMF) in the presence of a catalytic amount of cetyltrimethylammonium bromide ([CH_3_(CH_2_)_15_N(CH_3_)_3_]Br) at ambient temperature. The desired trifluoromethylated product was obtained with moderate yield and excellent diastereoselectivity (43 %, d.r.=94:6; Entry 1, Table 1). The yield improved to 65 % in the absence of cetyltrimethylammonium bromide (65 %, Entry 2). After screening several bases (Entries 1–9), the yield of **1 a** was increased to 76 % when the reaction was carried out using KOAc (Entry 9). The choice of solvent is crucially important in conversion, and the best result was obtained by treating **2 a** with Me_3_SiCF_3_ (2.0 equiv) at room temperature in dimethyl sulfoxide (DMSO) in the presence of KOAc (1.5 equiv). Substrate **2 a** was completely consumed in 3 h, and the desired product **1 a** was obtained in 91 % yield (Entry 13).

**Table 1:** Optimization of reaction conditions.

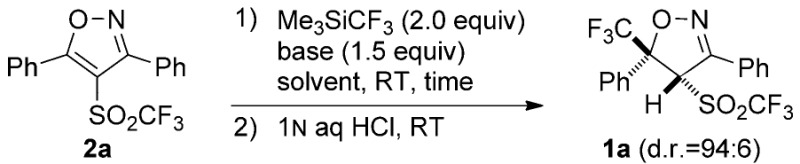				
Entry^[a]^	Base	Solvent	*t* [h]	Yield [%]^[b]^
1^[c]^	NaOAc	DMF	24	43
2	NaOAc	DMF	19	65
3	K_2_CO_3_	DMF	19	3
4	KOH	DMF	24	cp
5	*t*BuOK	DMF	19	17
6	KF	DMF	19	50
7	CsF	DMF	24	nr
8	LiOAc	DMF	19	22
9	KOAc	DMF	19	76
10	KOAc	DMI	20	trace
11	KOAc	DMA	20	6
12	KOAc	NMP	20	36
13	KOAc	DMSO	3	91

[a] The reaction of **2 a** with Me_3_SiCF_3_ (2.0 equiv) was carried out in the presence of base (1.5 equiv) at ambient temperature.

[b] Isolated yield.

[c] Cetyltrimethylammonium bromide (30 mol %) was added. cp=complex, nr=no reaction.

With optimal conditions in hand, the scope of the trifluoromethylation of isoxazole triflones **2** was explored with a variety of substrates selected in order to establish the generality of the process using this strategy (see Table 2). A series of 3,5-diary-4-triflyl-isoxazole **2 b**–**g** with a variety of substituents at their aromatic rings (Ar) such as methyl, methoxy, bromo, chloro, nitro as well as sterically demanding naphthyl moiety, were nicely converted to the corresponding trifluoromethylated adducts **1 b**–**g** in high to excellent yield (80–96 %) with excellent diastereoselectivities (d.r.=93:7–97:3; Table 2, Entries 2–7). Heteroaryl-substituted isoxazole triflone **2 h** was also a suitable substrate for this transformation, affording trifluoromethylated adduct **1 h** in 85 % yield with d.r.=99:1 (Entry 8). Interestingly, for isoxazole triflone **2 i**, which has an enolizable proton, the trifluoromethylation reaction proceeded nicely to provide the corresponding CF_3_ adduct **1 i** in good yield as a single diastereomer (Entry 9). We next examined the substrate scope differing in the nature of the aryl substituents of Ar^1^. A series of isoxazole triflones **2 j**–**o** were nicely converted to SO_2_CF_3_-substituted 5-trifluoromethyl-2-isoxazolines **1 j**–**o** in 87–99 % yield with high diastereoselectivities (d.r.=93:7–95:5), these being almost independent of the functional groups on the aromatic ring Ar^1^, such as, methyl, methoxy, chloro, bromo and nitro as well as sterically demanding naphthyl moiety (Entries 10–15). With facile access to this range of 5-trifluoromethyl-4-triflyl-2-isoxazolines **1**, we next considered the synthesis of multiply substituted triflyl-isoxazoline **1 p**. 5-Trifluoromethyl-2-isoxazoline **3** is a compound possessing high antiparasitic activity (Scheme 1);[13a] therefore, its SO_2_CF_3_ derivative **1 p** would be an attractive biologically active candidate (Figure 1). As expected, trifluoromethylation of **2 p** smoothly proceeded to give **1 p** in 98 % yield. The relative stereochemistry of (4S*,5R*)-**1 h** was clearly determined by X-ray analysis (Figure 2), and the stereochemistry of all the other products **1** were tentatively assigned by ^1^H and ^19^F NMR spectral comparison with **1 h**.

**Table 2:** Diastereoselective trifluoromethylation of isoxazole triflones 2 a–p.

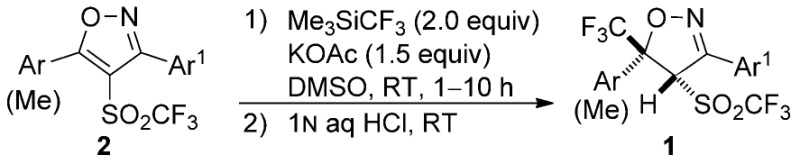						
Entry^[a]^	2	Ar (or Me)	Ar^1^	1	d.r.[b]	Yield [%]^[c]^
1	**2 a**	Ph	Ph	**1 a**	94:6	91
2^[d]^	**2 b**	4-MeC_6_H_4_	Ph	**1 b**	93:7	85
3	**2 c**	4-MeOC_6_H_4_	Ph	**1 c**	95:5	96
4^[d]^	**2 d**	4-ClC_6_H_4_	Ph	**1 d**	97:3	88
5	**2 e**	4-BrC_6_H_4_	Ph	**1 e**	96:4	90
6	**2 f**	4-NO_2_C_6_H_4_	Ph	**1 f**	97:3	80
7^[d]^	**2 g**	2-naphthyl	Ph	**1 g**	96:4	93
8	**2 h**	2-furanyl	Ph	**1 h**	99:1	85
9	**2 i**	Me	Ph	**1 i**	100:0	64
10	**2 j**	Ph	4-MeC_6_H_4_	**1 j**	93:7	87
11	**2 k**	Ph	4-MeOC_6_H_4_	**1 k**	93:7	89
12^[d]^	**2 l**	Ph	4-ClC_6_H_4_	**1 l**	94:6	89
13^[d]^	**2 m**	Ph	4-BrC_6_H_4_	**1 m**	94:6	89
14	**2 n**	Ph	4-NO_2_C_6_H_4_	**1 n**	95:5	92
15^[d]^	**2 o**	Ph	2-naphthyl	**1 o**	96:4	99
16	**2 p**	3,5-Cl_2_C_6_H_3_	4-MeOC_6_H_4_	**1 p**	95:5	98

[a] The reaction of **1** with Me_3_SiCF_3_ (2.0 equiv) was carried out in the presence of KOAc (1.5 equiv) in DMSO at ambient temperature, unless otherwise noted.

[b] Determined by ^19^F NMR.

[c] Isolated yield. [d] The reaction was carried out with Me_3_SiCF_3_ (4.0 equiv) and KOAc (3.0 equiv).

**Figure 2:**
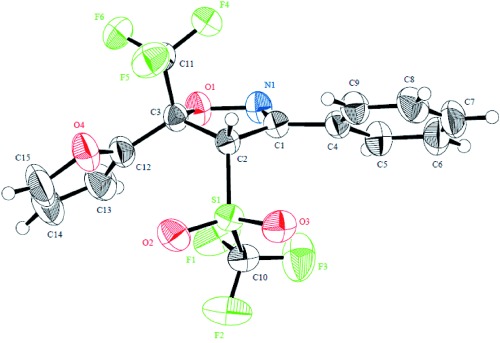
X-ray crystallographic analysis of 1 h (CCDC 866121).

Trifluoromethylation of cinnamyl-substituted isoxazole triflone **2 q** was next investigated (Scheme 2).[14a] Under the same reaction condition, trifluoromethylated adduct **1 q** at the 5-position of isoxazoline was regioselectively obtained in 80 % yield as single diastereomer, accompanied with small amounts of 1,6-conjugated adduct **4** (9 %).

**Scheme 2:**
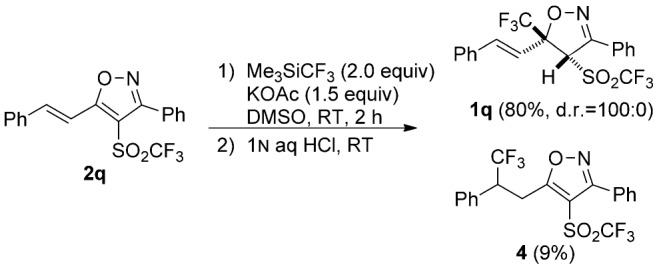
1,4-Regioselective trifluoromethylation of cinnamyl-substituted isoxazole triflone 2 q.

We finally examined the halogenation reactions of trifluoromethylated adduct **1 a** to all-carbon functionalized isoxazolines (Scheme 3), since 4-halogenated 3,5-diaryl-5-(trifluoromethyl)-2-isoxazoline derivatives have emerged as targets in 2009.[15a] Fluorination of **1 a** with Selectfluor® in acetonitrile gave a highly functionalized 4-fluoro-5-(trifluoromethyl)-4-(trifluoromethanesulfonyl)-2-isoxazoline **1 a-F** in high yield with good diastereoselectivity (89 %, d.r.=80:20). It should be noted that the fluorination of nitro-analogue **5** under the same reaction condition failed to provide any fluorination product. This phenomenon apparently resulted from strong lipophilic and electron-withdrawing features of the triflyl group. Other halogenation reactions were also smoothly achieved, namely chlorination and bromination, under simple conditions using *N*-chlorosuccinimide (NCS) and *N*-bromosuccinimide (NBS) to afford **1 a-Cl** and **1 a-Br** in excellent yields with 94 % and 96 %, respectively.

**Scheme 3:**
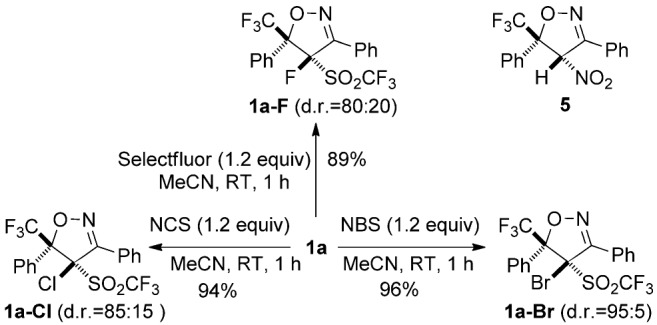
Halogenations of trifluoromethylated adduct 1 a to 1 a-X.

The trifluoromethylation/halogenation reactions proceeded in a one-pot sequential protocol without any loss of yield and selectivity. Namely, after completion of the first trifluoromethylation of **2 a** with Me_3_SiCF_3_, monitored by TLC analysis, a solution of halogenating reagents (Selectfluor®, NCS or NBS) in acetonitrile was added into the reaction mixture. It should be noted that both yields and diastereoselectivities were comparable to the results achieved for the two-step reactions (Scheme 4).

**Scheme 4:**
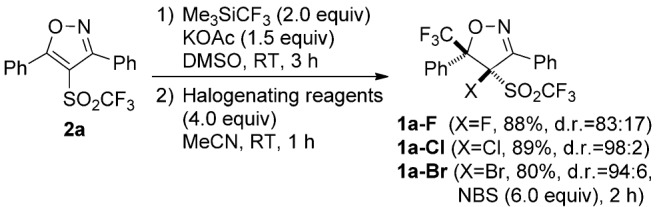
Sequential trifluomethylation/halogenation of 2 a to 1 a-X.

It is known that nucleophilic trifluoromethylation to conjugated alkenes essentially proceeds by a 1,2-addition, not a 1,4-addition except for several specific cases.[16a] We thus propose that this trifluoromethylation is explained by a 1,2-type addition of a CF_3_ anion to a species **A**, which is a resonance structure of **2 a**, to provide intermediates **B**. The electrophilic approach of H^+^ or X^+^ to **B** from the face opposite to the existing phenyl group furnish **1 a** or **1 a-X** with high diastereoselectivities (Scheme 5).

**Scheme 5:**
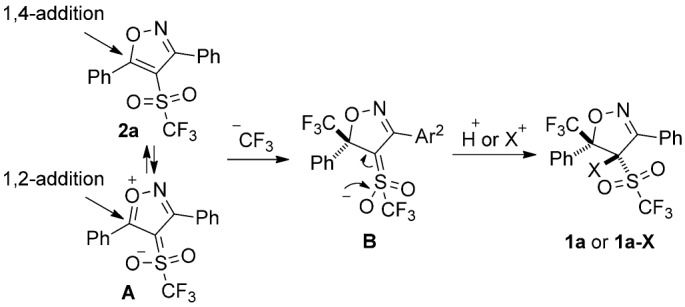
A proposed reaction mechanism from 2 a to 1 a-X.

In summary, activation of aromatic isoxazoles with a triflyl group at the 4-position realized the regio-and diastereoselective trifluoromethylation of isoxazole triflones **2** by Me_3_SiCF_3_ at the 5-position, which directly provided biologically potent, highly functionalized 3,5-disubstituted-5-(trifluoromethyl)-2-isoxazoline featuring the SO_2_CF_3_ group at the 4-position. Hence, the triflyl group not only activates the 5-position of aromatics and 4-position of resulting isoxazolines but also provides a new class of highly functionalized 5-trifluoromethyl-2-isoxazoline derivatives **1** attractive for agrochemicals. Biological activities of selected 5-(trifluoromethyl)-2-isoxazoline derivatives **1** and asymmetric variants of this method are now under consideration.
