# Supplementary material for: Diastereoselective Additive Trifluoromethylation/Halogenation of Isoxazole Triflones: Synthesis of All-Carbon-Functionalized Trifluoromethyl Isoxazoline Triflones
Source: ChemistryOpen. 2014 Feb 13;3(1):14–8. doi: 10.1002/open.201300044 (PMC3943607; doi:10.1002/open.201300044)
Supplement: Supplementary file 1 [file open0003-0014-sd1.pdf]

ChemistryOPEN

Supporting Information

© 2014 The Authors. Published by Wiley-VCH Verlag GmbH & Co. KGaA, Weinheim

Diastereoselective Additive Trifluoromethylation/ Halogenation of Isoxazole Triflones: Synthesis of All-Carbon-Functionalized Trifluoromethyl Isoxazoline Triflones

Hiroyuki Kawai,[a] Yutaka Sugita,[a] Etsuko Tokunaga,[a] Hiroyasu Sato,[b] Motoo Shiro,[b] and Norio Shibata*[a]

open_201300044_sm_miscellaneous_information.pdf

Experimental Section

General Methods:

All reactions were performed in oven-dried glassware under a positive pressure of nitrogen. Solvents were transferred *via* syringe and were introduced into the reaction vessels though a rubber septum. All reactions were monitored by thin-layer chromatography (TLC) carried out on 0.25 mm Merck silica-gel (60-F254). The TLC plates were visualized with UV light and 7% phosphomolybdic acid or $KMnO_4$ in water/heat. Column chromatography was carried out on a column packed with silica-gel 60N spherical neutral size 63-210 μm. The ^{1}H-NMR (300 MHz), ^{19}F-NMR (282 MHz), ^{13}C-NMR (150.9 MHz) spectra for solution in $CDCl_3$ or CD_3CN were recorded on a Buruker Avance 600 and a Varian Mercury 300. Chemical shifts (δ) are expressed in ppm downfield from internal TMS, $CHCl_3$ or CH_3CN. Mass spectra were recorded on a SHIMADZU LCMS-2010EV. Infrared spectra were recorded on a JASCO FT/ IR-200 spectrometer.

General procedure for the trifluoromethylation reaction of isoxazole triflones:

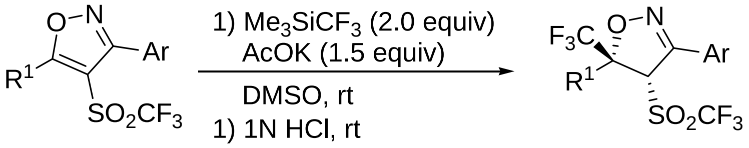

To a stirred suspension of isoxazole triflones **2** (0.10 mmol) and KOAc (14.7 mg, 0.15 mmol, 1.5 equiv) in DMSO (0.5 mL) was added Me_3SiCF_3 (29.6 μL, 0.20 mmol, 2.0 equiv) at room temperature under nitrogen atmosphere. After reaction mixture was stirred for appropriate time described below at the ambient temperature, 1N HCl aq. was added to the reaction mixture and stirred for 30 minutes. After dilution with water, the whole reaction mixture was extracted with AcOEt, and the combined organic layers was washed with brine, dried over Na_2SO_4 and concentrated under reduced pressure. The residue was purified by column chromatography on silica gel to give trifluoromethylated compounds **1**.

(4S*,5R*)-3,5-Diphenyl-5-(trifluoromethyl)-4-(trifluoromethylsulfonyl)-4,5-dihydroisoxazole (1a)

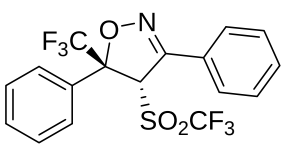

A reaction of isoxazole triflones **2a** (35.3 mg, 0.10 mmol), Me_3SiCF_3 (29.6 μL, 0.20 mmol, 2.0 equiv) and KOAc (14.7 mg, 0.15 mmol, 1.5 equiv) in DMSO (0.5 mL) for 3 h at the room temperature, and a purification by column chromatography on silica gel (*n*-hexane/ethyl acetate = 90/10) to give trifluoromethylated adduct **1a** (38.4 mg, 91%, dr = 94:6) as a white solid.
^{1}H NMR ($CDCl_3$, 300 MHz) δ 5.64 (s, 1H), 7.47-7.56 (m, 6H), 7.71-7.78 (m, 4H); ^{13}C NMR ($CDCl_3$, 150.9 MHz) δ 72.7 (m), 91.0 (q, *J* = 29.2 Hz), 118.8 (q, *J* = 330.5 Hz), 123.5 (q, *J* = 290.7 Hz), 125.8, 126.3, 128.0, 128.2, 128.5, 128.9, 130.7, 131.8, 153.0; ^{19}F NMR ($CDCl_3$, 282 MHz) (dr = 94:6) δ -78.7 (s, 0.94 × 3F), -73.0 (s, 0.94 × 3F), -71.6 (m, 0.06 × 3F), -68.8 (m, 0.06 × 3F); IR (KBr) 3076, 2913, 1499, 1451, 1387, 1332, 1217, 1166, 1112, 985, 952, 915, 885, 761, 745, 696, 625, 538, 503, 454 cm^{-1}; mp = 155.0-156.0 ºC ($CHCl_3$); MS (ESI, *m/z*) 422 [M−H]$^-$, HRMS (ESI) calcd. for $C_{17}H_{10}F_6NO_3S$ [M−H]$^-$: 422.0286 Found: 422.0288.

(4S*,5R*)-3-Phenyl-5-*p*-tolyl-5-(trifluoromethyl)-4-(trifluoromethylsulfonyl)-4,5-dihydroisoxazole (1b)

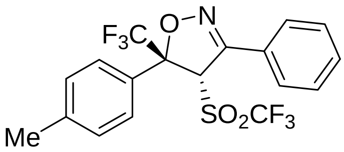

A reaction of isoxazole triflones **2b** (36.7 mg, 0.10 mmol), Me_3SiCF_3 (59.1 μL, 0.40 mmol, 4.0 equiv) and KOAc (29.4 mg, 0.30 mmol, 3.0 equiv) in DMSO (0.5 mL) for 1 h at the room temperature, and a purification by column chromatography on silica gel (*n*-hexane/ethyl acetate = 90/10) to give trifluoromethylated adduct **1b** (37.1 mg, 85%, dr = 93:7) as a white solid.

^{1}H NMR ($CDCl_3$, 300 MHz) δ 2.41 (s, 3H), 5.62 (s, 1H), 7.28 (d, *J* = 8.1 Hz, 2H), 7.46-7.57 (m, 3H), 7.64 (d, *J* = 8.4 Hz, 2H), 7.70-7.73 (m, 2H); ^{13}C NMR ($CDCl_3$, 150.9 MHz) δ 21.3, 72.6, 91.1 (q, J = 29.2 Hz), 118.8 (q, *J* = 330.5 Hz), 122.7, 123.5 (q, *J* = 290.7 Hz), 128.0, 128.4, 128.93, 128.96, 131.7, 140.9, 152.9 ; ^{19}F NMR ($CDCl_3$, 282 MHz) (dr = 93:7) δ -78.8 (s, 0.93 × 3F), -73.0 (s, 0.93 × 3F), -71.6 (m, 0.07 × 3F), -69.0 (m, 0.07 × 3F); IR (KBr) 3036, 2917, 1734, 1518, 1446, 1388, 1217, 1166, 1112, 944, 912, 884, 811, 763, 747, 690, 630, 553, 535, 505 cm^{-1}; mp = 133.0-136.0 ºC ($CHCl_3$); MS (ESI, *m/z*) 436 $[M-H]^-$, HRMS (ESI) calcd. for $C_{18}H_{12}F_6NO_3S$ $[M-H]^-$: 436.0442 Found: 436.0453.

(4S*,5R*)-5-(4-Methoxyphenyl)-3-phenyl-5-(trifluoromethyl)-4-(trifluoromethylsulfonyl)-4,5-dihydroisoxazole (1c)

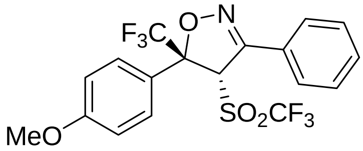

A reaction of isoxazole triflones **2c** (38.3 mg, 0.10 mmol), Me_3SiCF_3 (29.6 μL, 0.20 mmol, 2.0 equiv) and KOAc (14.7 mg, 0.15 mmol, 1.5 equiv) in DMSO (0.5 mL) for 2 h at the room temperature, and a purification by column chromatography on silica gel (*n*-hexane/ethyl acetate = 90/10) to give trifluoromethylated adduct **1c** (43.6 mg, 96%, dr = 95:5) as a white solid.

^{1}H NMR ($CDCl_3$, 300 MHz) δ 3.85 (s, 3H), 5.61 (s, 1H), 6.98 (d, J = 8.7 Hz, 2H), 7.47-7.57 (m, 3H), 7.66-7.72 (m, 4H); ^{13}C NMR ($CDCl_3$, 150.9 MHz) δ 55.3, 72.7, 91.0 (q, *J* = 29.7 Hz), 113.6, 117.3, 118.8 (q, *J* = 331.0 Hz), 123.5 (q, *J* = 290.7 Hz), 126.3, 128.0, 128.9, 130.1, 131.7, 152.9, 161.2; ^{19}F NMR ($CDCl_3$, 282 MHz) (dr = 95:5) δ -79.0 (s, 0.95 × 3F), -73.0 (s, 0.95 × 3F), -71.6 (m, 0.05 × 3F), -69.2 (m, 0.05 × 3F); IR (KBr) 2914, 2843, 1615, 1519, 1467, 1389, 1196, 1110, 1032, 986, 913, 822, 764, 691, 630, 586, 536, 446 cm^{-1}; mp = 130.0-131.0 ºC ($CHCl_3$); MS (ESI, *m/z*) 452 $[M-H]^-$, HRMS (ESI) calcd. for $C_{18}H_{12}F_6NO_4S$ $[M-H]^-$: 452.0391 Found: 452.0403.

(4S*,5R*)-5-(4-Chlorophenyl)-3-phenyl-5-(trifluoromethyl)-4-(trifluoromethylsulfonyl)-4,5-dihydroisoxazole (1d)

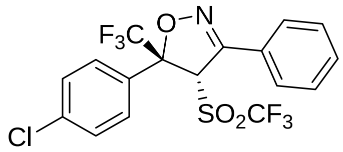

A reaction of isoxazole triflones **2d** (38.8 mg, 0.10 mmol), Me_3SiCF_3 (59.1 μL, 0.40 mmol, 4.0 equiv) and KOAc (29.4 mg, 0.30 mmol, 3.0 equiv) in DMSO (0.5 mL) for 1 h at the room temperature, and a purification by column chromatography on silica gel (*n*-hexane/ethyl acetate = 90/10) to give trifluoromethylated adduct **1d** (40.4 mg, 88%, dr = 97:3) as a white solid.

^{1}H NMR ($CDCl_3$, 300 MHz) δ 5.62 (s, 1H), 7.45-7.56 (m, 5H), 7.69-7.72 (m, 4H); ^{13}C NMR ($CDCl_3$, 150.9 MHz) δ 72.6 (m), 90.6 (q, *J* = 29.7 Hz), 118.7 (q, *J* = 330.5 Hz), 123.3 (q, *J* = 290.7 Hz), 124.3, 126.0, 128.0, 128.6, 129.0, 130.0, 131.9, 137.2, 153.0; ^{19}F NMR ($CDCl_3$, 282 MHz) (dr = 97:3) δ -78.8 (s, 0.97 × 3F), -73.0 (s, 0.97 × 3F), -71.5 (m, 0.03 × 3F), -68.8 (m, 0.03 × 3F); IR (KBr) 3060, 2916, 1600, 1497, 1446, 1388, 1333, 1207, 1112, 1018, 987, 946, 909, 822, 748, 690, 630, 539, 504, 445 cm^{-1}; mp = 154.0-155.5 ºC ($CHCl_3$); MS (ESI, *m/z*) 456 $[M-H]^-$, HRMS (ESI) calcd. for $C_{17}H_9ClF_6NO_3S$ $[M-H]^-$: 455.9896 Found: 455.9898.

(4S*,5R*)-5-(4-Bromophenyl)-3-phenyl-5-(trifluoromethyl)-4-(trifluoromethylsulfonyl)-4,5-dihydroisoxazole (1e)

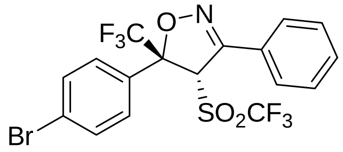

A reaction of isoxazole triflones **2e** (43.2 mg, 0.10 mmol), Me_3SiCF_3 (29.6 μL, 0.20 mmol, 2.0 equiv) and KOAc (14.7 mg, 0.15 mmol, 1.5 equiv) in DMSO (0.5 mL) for 2 h at the room temperature, and a purification by column chromatography on silica gel (*n*-hexane/ethyl acetate = 95/5) to give trifluoromethylated adduct **1e** (45.0 mg, 90%, dr = 96:4) as a white solid.

^{1}H NMR ($CDCl_3$, 300 MHz) δ 5.62 (s, 1H), 7.47-7.57 (m, 3H), 7.63 (s, 4H), 7.71 (d, *J* = 6.9 Hz, 2H); ^{13}C NMR ($CDCl_3$, 150.9 MHz) δ 72.9, 91.1 (q, *J* = 29.7 Hz), 119.2 (q, *J* = 330.5 Hz), 123.6 (q, *J* = 289.7 Hz), 125.2, 125.9, 126.4, 128.4, 129.4, 130.6, 132.0, 132.4, 153.4; ^{19}F NMR ($CDCl_3$, 282 MHz) (dr = 96:4) δ -78.8 (s, 0.96 × 3F), -72.9 (s, 0.96 × 3F), -71.4 (m, 0.04 × 3F), -68.8 (m, 0.04 × 3F); IR (KBr) 3060, 2915, 1594, 1495, 1446, 1388, 1332, 1205, 1118, 1015, 986, 946, 909, 884, 818, 763, 689, 629, 537, 503 cm^{-1}; mp = 145.0-146.5 ºC ($CHCl_3$); MS (ESI, *m/z*) 500 $[M-H]^-$, HRMS (ESI) calcd. for $C_{17}H_9BrF_6NO_3S$ $[M-H]^-$: 499.9391 Found: 499.9400.

(4S*,5R*)-5-(4-Nitrophenyl)-3-phenyl-5-(trifluoromethyl)-4-(trifluoromethylsulfonyl)-4,5-dihydroisoxazole (1f)

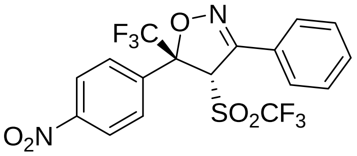

A reaction of isoxazole triflones **2f** (39.8 mg, 0.10 mmol), Me_3SiCF_3 (29.6 μL, 0.20 mmol, 2.0 equiv) and KOAc (14.7 mg, 0.15 mmol, 1.5 equiv) in DMSO (0.5 mL) for 10 h at the room temperature, and a purification by column chromatography on silica gel (*n*-hexane/ethyl acetate = 70/30) to give trifluoromethylated adduct **1f** (37.3 mg, 80%, dr = 97:3) as a off-white solid.
^{1}H NMR ($CDCl_3$, 300 MHz) δ 5.71 (s, 1H), 7.50-7.61 (m, 3H), 7.74 (d, *J* = 6,9 Hz, 2H), 7.99 (d, *J* = 8.4 Hz, 2H), 8.35 (d, *J* = 9.0 Hz, 2H); ^{13}C NMR ($CDCl_3$, 150.9 MHz) δ 72.5, 90.4 (q, *J* = 29.7 Hz), 118.6 (q, *J* = 330.5 Hz), 123.1 (q, *J* = 290.7 Hz), 123.2, 125.6, 128.0, 129.1, 130.0, 132.2, 132.6, 149.2, 153.1; ^{19}F NMR ($CDCl_3$, 282 MHz) (dr = 97:3) δ -78.4 (s, 0.97 × 3F), -73.1 (s, 0.97 × 3F), -71.2 (m, 0.03 × 3F), -68.2 (m, 0.03 × 3F); IR (KBr) 3125, 2916, 1705, 1604, 1523, 1449, 1385, 1356, 1217, 1117, 991, 911, 855, 801, 745, 712, 688, 625, 540, 498 cm^{-1}; mp = 180.0-181.0 ºC ($CHCl_3$); MS (ESI, *m/z*) 467 [M−H]$^-$, HRMS (ESI) calcd. for $C_{17}H_9F_6N_2O_5S$ [M−H]$^-$: 467.0136 Found: 467.0148.

(4S*,5R*)-5-(Naphthalen-2-yl)-3-phenyl-5-(trifluoromethyl)-4-(trifluoromethylsulfonyl)-4,5-dihydroisoxazole (1g)

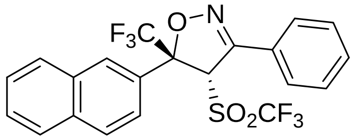

A reaction of isoxazole triflones **2g** (40.3 mg, 0.10 mmol), Me_3SiCF_3 (59.1 μL, 0.40 mmol, 4.0 equiv) and KOAc (29.4 mg, 0.30 mmol, 3.0 equiv) in DMSO (0.5 mL) for 2 h at the room temperature, and a purification by column chromatography on silica gel (*n*-hexane/ethyl acetate = 95/5) to give trifluoromethylated adduct **1g** (43.9 mg, 93%, dr = 96:4) as a white solid.
^{1}H NMR ($CDCl_3$, 300 MHz) δ 5.73 (s, 1H), 7.49-7.62 (m, 5H), 7.74-7.77 (m, 3H), 7.88-7.96 (m, 3H), 8.33 (s, 1H); ^{13}C NMR ($CDCl_3$, 150.9 MHz) δ 73.2, 91.7 (q, *J* = 29.2 Hz), 119.2 (q, *J* = 331.0 Hz), 123.5, 123.9 (q. *J* = 290.7 Hz), 125.2, 126.7, 127.2, 128.0, 128.2, 128.4, 129.3, 129.4, 129.5, 132.2, 132.8, 134.3, 153.4; ^{19}F NMR ($CDCl_3$, 282 MHz) (dr = 96:4) δ -78.3 (s, 0.96 × 3F), -72.9 (s, 0.96 × 3F), -71.5 (m, 0.04 × 3F), -68.5 (m, 0.04 × 3F); IR (KBr) 3068, 2920, 1508, 1445, 1386, 1332, 1214, 1164, 1111, 971, 910, 815, 748, 689, 625, 549, 533, 480 cm^{-1}; mp = 195.0-197.0 ºC ($CHCl_3$); MS

(ESI, *m/z*) 472 [M−H]$^-$, HRMS (ESI) calcd. for $C_{21}H_{12}F_6NO_3S$ [M−H]$^-$: 472.0442 Found: 472.0445.

(4S*,5R*)-5-(Furan-2-yl)-3-phenyl-5-(trifluoromethyl)-4-(trifluoromethylsulfonyl)-4,5-dihydroisoxazole (1h)

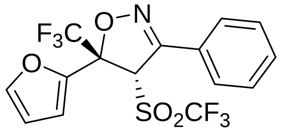

A reaction of isoxazole triflones **2h** (34.3 mg, 0.10 mmol), Me_3SiCF_3 (29.6 μL, 0.20 mmol, 2.0 equiv) and KOAc (14.7 mg, 0.15 mmol, 1.5 equiv) in DMSO (0.5 mL) for 2 h at the room temperature, and a purification by column chromatography on silica gel (*n*-hexane/ethyl acetate = 90/10) to give trifluoromethylated adduct **1h** (35.2 mg, 85%, dr = 99:1) as a white solid.
^{1}H NMR ($CDCl_3$, 300 MHz) δ 5.58 (s, 1H), 6.53 (d, *J* = 1.5 Hz, 1H), 6.87 (d, *J* = 3.3 Hz, 1H), 7.47-7.56 (m, 3H), 7.61 (s, 1H), 7.71 (d, *J* = 7.2 Hz, 2H); ^{13}C NMR ($CDCl_3$, 150.9 MHz) δ 71.6 (m), 87.9 (q, *J* = 31.7 Hz), 111.2, 114.7, 118.9 (q, *J* = 330.5 Hz), 122.5 (q, *J* = 290.7 Hz), 125.8, 128.0, 129.0, 132.0, 138.7, 145.1, 152.4; ^{19}F NMR ($CDCl_3$, 282 MHz) (dr = 99:1) δ -79.5 (s, 0.99 × 3F), -72.9 (s, 0.99 × 3F), -71.8 (m, 0.01 × 3F), -69.5 (m, 0.01 × 3F); IR (KBr) 3132, 2913, 1499, 1446, 1388, 1334, 1209, 1111, 1030, 1000, 968, 912, 889, 763, 748, 690, 627, 593, 539, 498 cm^{-1}; mp = 120.0-121.5 ºC ($CHCl_3$); MS (ESI, *m/z*) 412 [M−H]$^-$, HRMS (ESI) calcd. for $C_{15}H_8F_6NO_4S$ [M−H]$^-$: 412.0078 Found: 412.0092.

(4S*,5R*)-5-Methyl-3-phenyl-5-(trifluoromethyl)-4-(trifluoromethylsulfonyl)-4,5-dihydroisoxazole (1i)

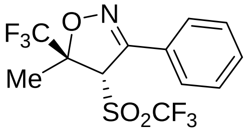

A reaction of isoxazole triflones **2i** (29.1 mg, 0.10 mmol), Me_3SiCF_3 (29.6 μL, 0.20 mmol, 2.0 equiv) and KOAc (14.7 mg, 0.15 mmol, 1.5 equiv) in DMSO (0.5 mL) for 2 h at the room temperature, and a purification by column chromatography on silica gel (*n*-hexane/acetone = 95/5) to give trifluoromethylated adduct **1i** (23.3 mg, 64%, single diastereomer) as a white solid.
^{1}H NMR ($CDCl_3$, 300 MHz) δ 2.04 (s, 3H), 5.26 (s, 1H), 7.44-7.53 (m, 3H), 7.68 (d, *J* = 6.9 Hz, 2H); ^{13}C NMR ($CDCl_3$, 150.9 MHz) δ 14.3, 71.1, 88.1 (q, *J* = 29.7 Hz), 119.0 (q, *J* = 330.0 Hz), 123.6 (q, *J* = 288.7 Hz), 126.0, 127.9, 128.9, 131.8, 151.7; ^{19}F NMR ($CDCl_3$, 282 MHz) (dr = 100:0) δ -83.4 (s, 3F), -73.3 (s, 3F); IR (KBr) 3072, 2906, 1447, 1385, 1167, 1111, 913, 896, 802, 764, 746, 715, 691, 635, 611, 580, 546, 530, 484, 441 cm^{-1}; mp = 85.0-87.5 ºC ($CHCl_3$); MS (ESI, *m/z*) 360 [M−H]$^-$, HRMS (ESI) calcd. for $C_{12}H_8F_6NO_3S$ [M−H]$^-$: 360.0129 Found: 360.0127.

(4S*,5R*)-5-Phenyl-3-*p*-tolyl-5-(trifluoromethyl)-4-(trifluoromethylsulfonyl)-4,5-dihydroisoxazole (1j)

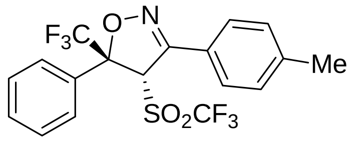

A reaction of isoxazole triflones **2j** (36.7 mg, 0.10 mmol), Me_3SiCF_3 (29.6 μL, 0.20 mmol, 2.0 equiv) and KOAc (14.7 mg, 0.15 mmol, 1.5 equiv) in DMSO (0.5 mL) for 10 h at the room temperature, and a purification by column chromatography on silica gel (*n*-hexane/ethyl acetate = 95/5) to give trifluoromethylated adduct **1j** (37.9 mg, 87%, dr = 93:7) as a white solid.
^{1}H NMR ($CDCl_3$, 300 MHz) δ 2.42 (s, 3H), 5.63 (s, 1H), 7.30 (d, J = 8.1 Hz, 2H), 7.44-7.49 (m, 3H), 7.62 (d, J = 7.8 Hz, 2H), 7.76 (d, J = 6.9 Hz, 2H); ^{13}C NMR ($CDCl_3$, 150.9 MHz) δ 21.6, 72.8, 90.8 (q, J = 29.7 Hz), 118.8 (q, J = 330.5 Hz), 123.5 (q, J = 291.2 Hz), 123.4, 125.9, 127.9, 128.2, 128.6, 129.7, 130.6, 142.4, 152.9; ^{19}F NMR ($CDCl_3$, 282 MHz) (dr = 93:7) δ -78.7 (s, 0.93 × 3F), -72.9 (s, 0.93 × 3F), -71.4 (m, 0.07 × 3F), -68.8 (m, 0.07 × 3F); IR (KBr) 3033, 2912, 1609, 1499, 1452, 1387, 1333, 1217, 1165, 1112, 985, 952, 918, 886, 815, 749, 695, 625, 539, 507 cm^{-1}; mp = 153.5-154.5 ºC ($CHCl_3$); MS (ESI, *m/z*) 436 [M−H]$^-$, HRMS (ESI) calcd. for $C_{18}H_{12}F_6NO_3S$ [M−H]$^-$: 436.0442 Found: 436.0448.

(4S*,5R*)-3-(4-Methoxyphenyl)-5-phenyl-5-(trifluoromethyl)-4-(trifluoromethylsulfonyl)-4,5-dihydroisoxazole (1k)

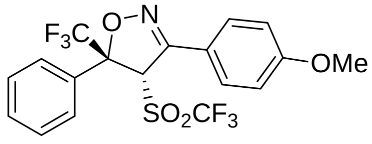

A reaction of isoxazole triflones **2k** (38.3 mg, 0.10 mmol), Me_3SiCF_3 (29.6 μL, 0.20 mmol, 2.0 equiv) and KOAc (14.7 mg, 0.15 mmol, 1.5 equiv) in DMSO (0.5 mL) for 2 h at the room temperature, and a purification by column chromatography on silica gel (*n*-hexane/ethyl acetate = 95/5) to give trifluoromethylated adduct **1k** (40.2 mg, 89%, dr = 93:7) as a white solid.
^{1}H NMR ($CDCl_3$, 300 MHz) δ 3.86 (s, 3H), 5.61 (s, 1H), 6.99 (d, J = 8.7 Hz, 2H), 7.44-7.49 (m, 3H), 7.68 (d, J = 8.4 Hz, 2H), 7.76 (d, J = 7.8 Hz, 2H); ^{13}C NMR ($CDCl_3$, 150.9 MHz) δ 55.4, 73.0 (m), 90.7 (q, J = 29.2 Hz), 114.4, 118.5, 118.8 (q, J = 331.0 Hz), 123.5 (q, J = 290.7 Hz), 125.9, 128.2, 128.6, 129.7, 130.6, 152.4, 162.4; ^{19}F NMR ($CDCl_3$, 282 MHz) (dr = 93:7) δ -78.6 (s, 0.93 × 3F), -72.8 (s, 0.93 × 3F), -71.3 (m, 0.07 × 3F), -68.8 (m, 0.07 × 3F); IR (KBr) 2960, 2913, 2837, 1610, 1518, 1452, 1389, 1339, 1022, 953, 912, 828, 761, 724, 696, 626, 539, 471 cm^{-1}; mp = 156.5-157.5 ºC ($CHCl_3$); MS (ESI, *m/z*) 452 [M−H]$^-$, HRMS (ESI) calcd. for $C_{18}H_{12}F_6NO_4S$ [M−H]$^-$: 452.0391

Found: 452.0389.

(4S*,5R*)-3-(4-Chlorophenyl)-5-phenyl-5-(trifluoromethyl)-4-(trifluoromethylsulfonyl)-4,5-dihydroisoxazole (1l)

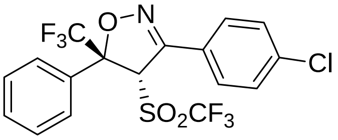

A reaction of isoxazole triflones **2l** (38.8 mg, 0.10 mmol), Me_3SiCF_3 (59.1 μL, 0.40 mmol, 4.0 equiv) and KOAc (29.4 mg, 0.30 mmol, 3.0 equiv) in DMSO (0.5 mL) for 2 h at the room temperature, and a purification by column chromatography on silica gel (*n*-hexane/ethyl acetate = 95/5) to give trifluoromethylated adduct **1l** (40.7 mg, 89%, dr = 94:6) as a white solid.

^{1}H NMR ($CDCl_3$, 300 MHz) δ 5.60 (s, 1H), 7.47-7.50 (m, 5H), 7.68 (d, *J* = 8.4 Hz, 2H), 7.75 (d, *J* = 6.6 Hz, 2H); ^{13}C NMR ($CDCl_3$, 150.9 MHz) δ 73.1 (m), 91.5 (q, *J* = 29.2 Hz), 119.2 (q, *J* = 331.0 Hz), 123.8 (q, *J* = 291.2 Hz), 125.2, 125.9, 128.7, 128.9, 129.67, 129.74, 131.2, 138.6, 152.5; ^{19}F NMR ($CDCl_3$, 282 MHz) (dr = 94:6) δ -78.8 (s, 0.94 × 3F), -72.7 (s, 0.94 × 3F), -71.2 (m, 0.06 × 3F), -68.9 (m, 0.06 × 3F); IR (KBr) 3081, 2909, 1716, 1600, 1495, 1452, 1388, 1332, 1206, 1111, 953, 924, 796, 752, 718, 696, 624, 535, 501 cm^{-1}; mp = 128.5-130.0 ºC ($CHCl_3$); MS (ESI, *m/z*) 456 $[M-H]^-$, HRMS (ESI) calcd. for $C_{17}H_9ClF_6NO_3S$ $[M-H]^-$: 455.9896 Found: 455.9907.

(4S*,5R*)-3-(4-Bromophenyl)-5-phenyl-5-(trifluoromethyl)-4-(trifluoromethylsulfonyl)-4,5-dihydroisoxazole (1m)

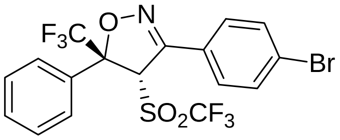

A reaction of isoxazole triflones **2m** (43.2 mg, 0.10 mmol), Me_3SiCF_3 (59.1 μL, 0.40 mmol, 4.0 equiv) and KOAc (29.4 mg, 0.30 mmol, 3.0 equiv) in DMSO (0.5 mL) for 2 h at the room temperature, and a purification by column chromatography on silica gel (*n*-hexane/ethyl acetate = 95/5) to give trifluoromethylated adduct **1m** (44.7 mg, 89%, dr = 94:6) as a white solid.

^{1}H NMR ($CDCl_3$, 300 MHz) δ 5.60 (s, 1H), 7.47-7.51 (m, 3H), 7.59-7.67 (m, 4H), 7.75 (d, *J* = 6.6 Hz, 2H); ^{13}C NMR ($CDCl_3$, 150.9 MHz) δ 72.6 (m), 91.1 (q, *J* = 29.2 Hz), 118.8 (q, *J* = 330.5 Hz), 123.4 (q, *J* = 290.2 Hz), 125.2, 125.5, 126.5, 128.3, 128.5, 129.4, 130.8, 132.3, 152.2; ^{19}F NMR ($CDCl_3$, 282 MHz) (dr = 94:6) δ -78.8 (s, 0.94 × 3F), -72.6 (s, 0.94 × 3F), -71.2 (m, 0.06 × 3F), -68.9 (m, 0.06 × 3F); IR (KBr) 3080, 2908, 1586, 1490, 1387, 1329, 1217, 1111, 1011, 985, 952, 924, 885, 822, 750, 714, 695, 622, 537, 508 cm^{-1}; mp = 147.5-149.0 ºC ($CHCl_3$); MS (ESI, *m/z*) 500 $[M-H]^-$,

HRMS (ESI) calcd. for $C_{17}H_9BrF_6NO_3S$ $[M-H]^-$: 499.9391 Found: 499.9402.

(4S*,5R*)-3-(4-Nitrophenyl)-5-phenyl-5-(trifluoromethyl)-4-(trifluoromethylsulfonyl)-4,5-dihydroisoxazole (1n)

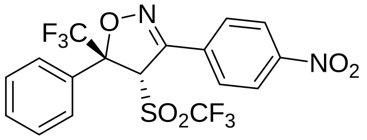

A reaction of isoxazole triflones **2n** (39.8 mg, 0.10 mmol), Me_3SiCF_3 (29.6 µL, 0.20 mmol, 2.0 equiv) and KOAc (14.7 mg, 0.15 mmol, 1.5 equiv) in DMSO (0.5 mL) for 5 h at the room temperature, and a purification by column chromatography on silica gel (*n*-hexane/ethyl acetate = 90/10) to give trifluoromethylated adduct **1n** (43.1 mg, 92%, dr = 95:5) as a off-white solid.
^{1}H NMR ($CDCl_3$, 300 MHz) δ 5.71 (s, 1H), 7.50-7.53 (m, 3H), 7.75 (q, *J* = 7.2 Hz, 2H), 7.96 (q, *J* = 8.7 Hz, 2H), 8.36 (d, *J* = 8.7 Hz); ^{13}C NMR ($CDCl_3$, 150.9 MHz) δ 72.6 (m), 91.6 (q, *J* = 29.2 Hz), 118.8 (q, *J* = 331.0 Hz), 123.3 (q, *J* = 290.2 Hz), 124.1, 125.1, 128.4, 129.1, 131.0, 132.3, 149.5, 151.5; ^{19}F NMR ($CDCl_3$, 282 MHz) (dr = 95:5) δ -78.9 (s, 0.95 × 3F), -72.4 (s, 0.95 × 3F), -70.9 (m, 0.05 × 3F), -68.9 (m, 0.05 × 3F); IR (KBr) 3124, 2953, 1716, 1610, 1574, 1521, 1453, 1383, 1214, 1115, 982, 952, 925, 853, 748, 715, 693, 624, 592, 504 cm^{-1}; mp = 155.0-158.0 ºC ($CHCl_3$); MS (ESI, *m/z*) 467 $[M-H]^-$, HRMS (ESI) calcd. for $C_{17}H_9F_6N_2O_5S$ $[M-H]^-$: 467.0136 Found: 467.0144.

(4S*,5R*)-3-(Naphthalen-2-yl)-5-phenyl-5-(trifluoromethyl)-4-(trifluoromethylsulfonyl)-4,5-dihydroisoxazole (1o)

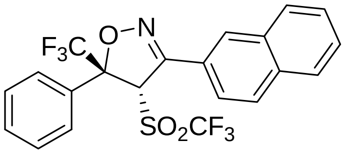

A reaction of isoxazole triflones **2o** (40.3 mg, 0.10 mmol), Me_3SiCF_3 (59.1 µL, 0.40 mmol, 4.0 equiv) and KOAc (29.4 mg, 0.30 mmol, 3.0 equiv) in DMSO (0.5 mL) for 2 h at the room temperature, and a purification by column chromatography on silica gel (*n*-hexane/ethyl acetate = 90/10) to give trifluoromethylated adduct **1o** (46.8 mg, 99%, dr = 96:4) as a white solid.
^{1}H NMR ($CDCl_3$, 300 MHz) δ 5.79 (s, 1H), 7.48-7.52 (m, 3H), 7.58-7.63 (m, 2H), 7.80 (d, *J* = 7.2 Hz, 2H), 7.86-7.97 (m, 4H), 8.17 (s, 1H); ^{13}C NMR ($CDCl_3$, 150.9 MHz) δ 72.8 (m), 91.1 (q, *J* = 29.2 Hz), 118.8 (q, *J* = 331.0 Hz), 123.5 (q, *J* = 290.7 Hz), 123.7, 123.9, 125.8, 127.2, 127.9, 128.2, 128.3, 128.6, 128.8, 128.99, 129.04, 130.7, 132.6, 134.7, 153.0; ^{19}F NMR ($CDCl_3$, 282 MHz) (dr = 96:4) δ -78.6 (s, 0.96 × 3F), -72.8 (s, 0.96 × 3F), -71.3 (m, 0.04 × 3F), -68.7 (m, 0.04 × 3F); IR (KBr) 3066, 2914, 1500, 1450, 1389, 1168, 1110, 985, 956, 906, 819, 795, 760, 716, 697, 616, 538,

511, 481 cm^{-1}; mp = 195.5-196.5 ºC ($CHCl_3$); MS (ESI, *m/z*) 472 [M−H]$^-$, HRMS (ESI) calcd. for $C_{21}H_{12}F_6NO_3S$ [M−H]$^-$: 472.0442 Found: 472.0449.

(4S*,5R*)-5-(3,5-Dichlorophenyl)-3-(4-methoxyphenyl)-5-(trifluoromethyl)-4-(trifluoromethylsulfonyl)-4,5-dihydroisoxazole (1p)

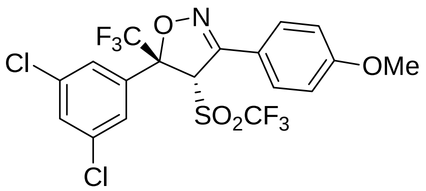

A reaction of isoxazole triflones **2p** (45.2 mg, 0.10 mmol), Me_3SiCF_3 (29.6 µL, 0.20 mmol, 2.0 equiv) and KOAc (14.7 mg, 0.15 mmol, 1.5 equiv) in DMSO (0.5 mL) for 2 h at the room temperature, and a purification by column chromatography on silica gel (*n*-hexane/ethyl acetate = 90/10) to give trifluoromethylated adduct **1p** (50.9 mg, 98%, dr = 95:5) as a white solid.

^{1}H NMR ($CDCl_3$, 300 MHz) δ 3.88 (s, 3H), 5.56 (s, 1H), 7.00 (d, *J* = 8.7 Hz, 2H), 7.52 (s, 1H), 7.65-7.68 (m, 4H); ^{13}C NMR ($CDCl_3$, 150.9 MHz) δ 55.5, 72.6 (m), 89.6 (q, *J* = 29.7 Hz), 114.5, 118.7 (q, *J* = 330.5 Hz), 123.0 (q, *J* = 291.2 Hz), 127.2, 129.1, 129.7, 130.9, 135.1, 152.7, 162.6 ;^{19}F NMR ($CDCl_3$, 282 MHz) (dr = 95:5) δ -78.5 (s, 0.95 × 3F), -72.9 (s, 0.95 × 3F), -70.9 (m, 0.05 × 3F), -68.4 (m, 0.05 × 3F); IR (KBr) 2959 1609, 1572, 1518, 1426, 1382, 1306, 1268, 1207, 1174, 1105, 1018, 868, 844, 806, 679, 621, 572, 502 cm^{-1}; mp = 146.0-147.5 ºC ($CHCl_3$); MS (ESI, *m/z*) 520 [M−H]$^-$, HRMS (ESI) calcd. for $C_{18}H_{10}C_{l2}F_6NO_4S$ [M−H]$^-$: 519.9612 Found: 519.9627.

(4S*,5R*)-(*E*)-3-Phenyl-5-styryl-5-(trifluoromethyl)-4-(trifluoromethylsulfonyl)-4,5-dihydroisoxazole (1q)

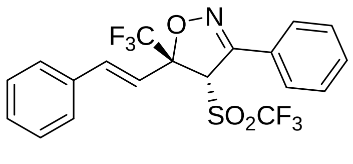

A reaction of isoxazole triflones **2q** (37.9 mg, 0.10 mmol), Me_3SiCF_3 (59.1 µL, 0.40 mmol, 4.0 equiv) and KOAc (29.4 mg, 0.30 mmol, 3.0 equiv) in DMSO (0.5 mL) for 2 h at the room temperature, and a purification by column chromatography on silica gel (*n*-hexane/ethyl acetate = 95/5) to give trifluoromethylated adduct **1q** (36.0 mg, 80%, single diastereomer) as a white solid.

^{1}H NMR ($CDCl_3$, 300 MHz) δ 5.49 (s, 1H), 6.50 (d, *J* = 16.2 Hz, 1H), 7.25 (d, *J* = 15.6 Hz, 1H), 7.36-7.42 (m, 3H), 7.47-7.56 (m, 5H), 7.71 (d, *J* = 7.2 Hz, 2H); ^{13}C NMR ($CDCl_3$, 150.9 MHz) δ 73.3 (m), 89.1 (q, J = 30.2 Hz), 113.2, 118.9 (q, J = 330.5 Hz), 123.0 (q, J = 289.7 Hz), 125.8, 127.3, 128.1, 128.9, 129.0, 129.4, 131.9, 134.8, 137.7, 151.9; ^{19}F NMR ($CDCl_3$, 282 MHz) (dr = 100:0) δ

-81.8 (s, 3F), -72.2 (s, 3F); IR (KBr) 3073, 2898, 1629, 1534, 1446, 1383, 1229, 1171, 1109, 964, 925, 894, 805, 753, 690, 632, 579, 532, 495 cm^{-1}; mp = 182.0-183.0 ºC ($CHCl_3$); MS (ESI, *m/z*) 448 $[M−H]^-$, HRMS (ESI) calcd. for $C_{19}H_{12}F_6NO_3S$ $[M−H]^-$: 448.0442 Found: 448.0450.

(4S*,5R*)-4-Fluoro-3,5-diphenyl-5-(trifluoromethyl)-4-(trifluoromethylsulfonyl)-4,5-dihydroisoxazole (1a-F)

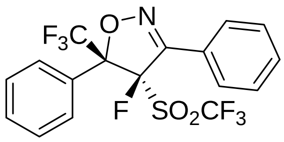

To a stirred solution of **1a** (127 mg, 0.3 mmol) in MeCN (6.0 mL) was added Selectfluor® (128 mg, 0.36 mmol, 1.2 equiv) at room temperature under nitrogen atmosphere, and then the reaction mixture was stirred for 1 h at the same temperature. After concentration under reduced pressure, the residue was purified by column chromatography on silica gel (*n*-hexane/ethyl acetate = 90/10) to give **1a-F (**118 mg, 89%, dr = 80:20) as a white solid.

(major isomer): white solid; ^1H NMR (CD_3CN, 300 MHz) δ 7.55-7.71 (m, 6H), 7.82-7.86 (m, 4H); ^{13}C NMR (CD_3CN, 150.9 MHz) δ 91.5 (dq, *J* = 15.5, 28.7 Hz), 119.8 (q, *J* = 332.0 Hz), 123.4 (dq, *J* = 5.7 Hz, 291.7 Hz), 124.7, 125.3, 129.0 (d, *J* = 3.0 Hz), 129.5, 129.7, 130.3, 132.7, 133.7, 154.0 (d, *J* = 15.1 Hz); ^{19}F NMR (CD_3CN, 282 MHz) δ -162.0 (qq, *J* = 8.2, 16.9 Hz, 1F), -71.6 (d, *J* = 16.6 Hz, 3F), -69.8 (d, *J* = 7.9 Hz, 3F); IR (KBr) 3068, 1601, 1496, 1450, 1393, 1332, 1215, 1113, 1028, 966, 899, 834, 759, 693, 618, 570, 528, 508 cm^{-1}; mp = 100.5-103.0 ºC (CH_3CN); MS (ESI, *m/z*) 422 $[M-F]^+$, HRMS calcd. for $C_{17}H_{10}F_6NO_3S$: 422.0286 $[M-F]^+$: Found: 422.0286.

(minor isomer): white solid; ^1H NMR (CD_3CN, 300 MHz) δ 7.48-7.55 (m, 4H), 7.58-7.64 (m, 4H), 7.72 (d, *J* = 6.9 Hz, 2H); ^{13}C NMR (CD_3CN, 150.9 MHz) δ 95.6 (m), 120.3 (q, *J* = 333.0 Hz), 122.8 (dq, *J* = 4.5, 285.2 Hz), 124.7, 126.2 (d, *J* = 6.0 Hz), 128.6, 129.8, 130.1, 130.3, 132.3, 133.3, 154.0 (d, *J* = 15.1 Hz); ^{19}F NMR (CD_3CN, 282 MHz) δ -147.2 (q, *J* = 13.5 Hz, 1F), -69.0 (d, *J* = 13.0 Hz, 3F), -66.7 (s, 3F); IR (KBr) 3073, 2928, 1964, 1600, 1450, 1397, 1208, 1095, 1025, 925, 904, 864, 761, 743, 692, 635, 619, 563, 513, 489 cm^{-1}; mp = 63.0-65.0 ºC (CH_3CN); MS (ESI, *m/z*) 422 $[M-F]^+$, HRMS calcd. for $C_{17}H_{10}F_6NO_3S$: 422.0286 $[M-F]^+$: Found: 422.0284.

(4R*,5R*)-4-Chloro-3,5-diphenyl-5-(trifluoromethyl)-4-(trifluoromethylsulfonyl)-4,5-dihydroisoxazole (1a-Cl)

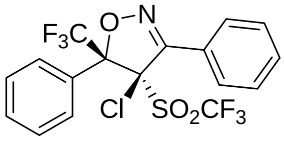

To a stirred solution of **2a** (106 mg, 0.25 mmol) in MeCN (5.0 mL) was added NCS (40.1 mg, 0.3 mmol, 1.2 equiv) at room temperature under nitrogen atmosphere, and then the reaction mixture was stirred for 1 h at the same temperature. After concentration under reduced pressure, the residue was purified by column chromatography on silica gel (*n*-hexane/ethyl acetate = 90/10) to give **1a-Cl (**108 mg, 94%, dr = 85:15) as a pale yellow oil.

(major isomer): off-white solid; ^{1}H NMR (CD_3CN, 300 MHz) δ 7.52-7.69 (m, 6H), 7.84-7.91 (m, 2H), 7.98 (d, J = 7.5 Hz); ^{19}F NMR (CD_3CN, 282 MHz) δ -71.7 (s, 3F), -66.3 (s, 3F); ^{13}C NMR (CD_3CN, 150.9 MHz) δ 93.2 (q, J = 27.7 Hz), 120.5 (q, J = 333.5 Hz), 123.7 (q, J = 292.7 Hz), 125.3, 126.0, 128.9, 129.80, 129.84, 129.89, 130.0, 132.5, 133.4, 156.2; ^{19}F NMR (CD_3CN, 282 MHz) δ -71.7 (s, 3F), -66.3 (s, 3F); IR (KBr) 3070, 2925, 1964, 1898, 1763, 1600, 1497, 1450, 1388, 1309, 1204, 1105, 1011, 961, 756, 688, 616, 580, 516 cm^{-1}; mp = 67.0-69.0 ºC (CH_3CN); MS (ESI, *m/z*) 422 [M-Cl]$^{+}$, HRMS calcd. for $C_{17}H_{10}F_6NO_3S$: 422.0286 [M-Cl]$^{+}$: Found: 422.0291.

(4R*,5R*)-4-Chloro-3,5-diphenyl-5-(trifluoromethyl)-4-(trifluoromethylsulfonyl)-4,5-dihydroisoxazole (1a-Br)

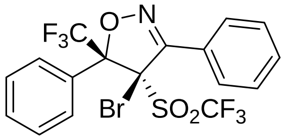

To a stirred solution of **2a** (423 mg, 1.0 mmol) in MeCN (10.0 mL) was added NBS (214 mg, 1.2 mmol, 1.2 equiv) at room temperature under nitrogen atmosphere, and then the reaction mixture was stirred for 1 h at the same temperature. After concentration under reduced pressure, the residue was purified by column chromatography on silica gel (*n*-hexane/ethyl acetate = 90/10) to give **1a-Br (**482 mg, 96%, dr = 95:5) as a off-white solid.

^{1}H NMR (CD_3CN, 300 MHz) δ 7.52-7.66 (m, 7H), 7.82 (d, J = 7.5 Hz, 1H), 7.97 (d, J = 7.5 Hz, 2H); ^{13}C NMR (CD_3CN, 150.9 MHz) δ 92.9 (q, J = 27.2 Hz), 119.9 (q, J = 333.5 Hz), 123.7 (q, J = 292.7 Hz), 125.5, 126.2, 128.7, 128.9, 129.6, 129.9, 130.3, 132.3, 133.2, 157.2; ^{19}F NMR (CD_3CN, 282 MHz) (dr = 95:5) δ -72.1 (s, 0.95 × 3F), -67.8 (s, 0.05 × 3F), -66.0 (s, 0.95 × 3F), -64.1 (s, 0.05 × 3F); IR (KBr) 3074, 2925, 1962, 1901, 1818, 1598, 1492, 1452, 1384, 1309, 1181, 1104, 1007, 932, 879, 757, 712, 616, 573, 508 cm^{-1}; mp = 110.0-111.5 ºC (CH_3CN); MS (ESI, *m/z*) 422 [M-Br]$^{+}$, HRMS calcd. for $C_{17}H_{10}F_6NO_3S$: 422.0286 [M-Br]$^{+}$: Found: 422.0284.

One-pot procedure (1a-F, Cl, Br)

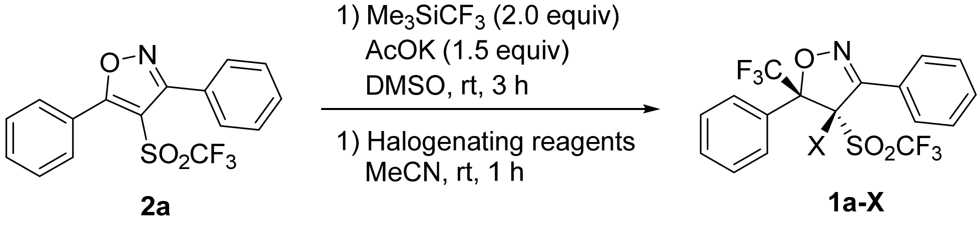

To a stirred suspension of isoxazole triflone **2a** (0.10 mmol) and KOAc (14.7 mg, 0.15 mmol, 1.5 equiv) in DMSO (0.5 mL) was added Me_3SiCF_3 (29.6 μL, 0.20 mmol, 2.0 equiv) at room temperature under nitrogen atmosphere. After reaction mixture was stirred for 3 h at the same temperature and the complete consumption of **2a** was checked by TLC analysis, halogenating reagent (Selectfluor® and NCS, 4.0 equiv; NBS. 6.0 equiv) in MeCN (0.5 mL) was added to the reaction mixture and stirred for 1 h. After dilution with water, the whole reaction mixture was extracted with Et_2O, and the combined organic layers was washed with brine, dried over Na_2SO_4 and concentrated under reduced pressure. The residue was purified by column chromatography on silica gel to give **1a-F** (39.0 mg, 88%, dr = 83:17), **1a-Cl** (40.7 mg, 89%, dr = 98:2), **1a-Br** (40.4 mg, 80%, dr = 94:6) as a white solid.

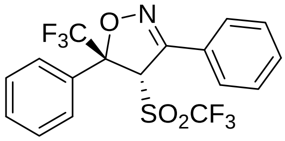

(4S*,5R*)-**1a**
(dr = 94:6)
^{1}H NMR

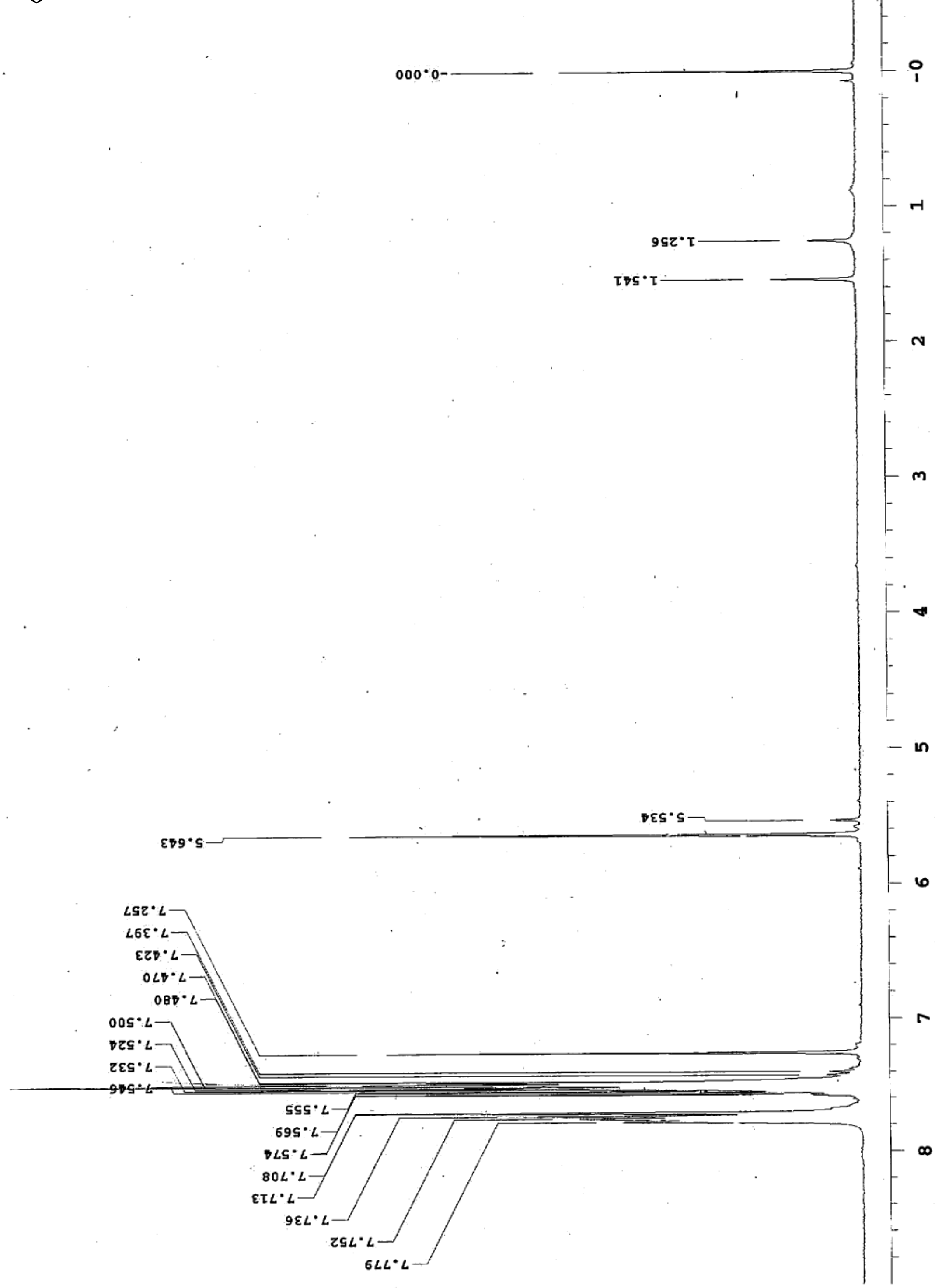

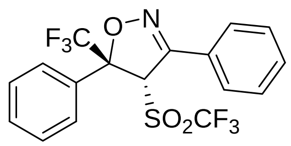

(4S*,5R*)-**1a**
(dr = 94:6)
^{19}F NMR

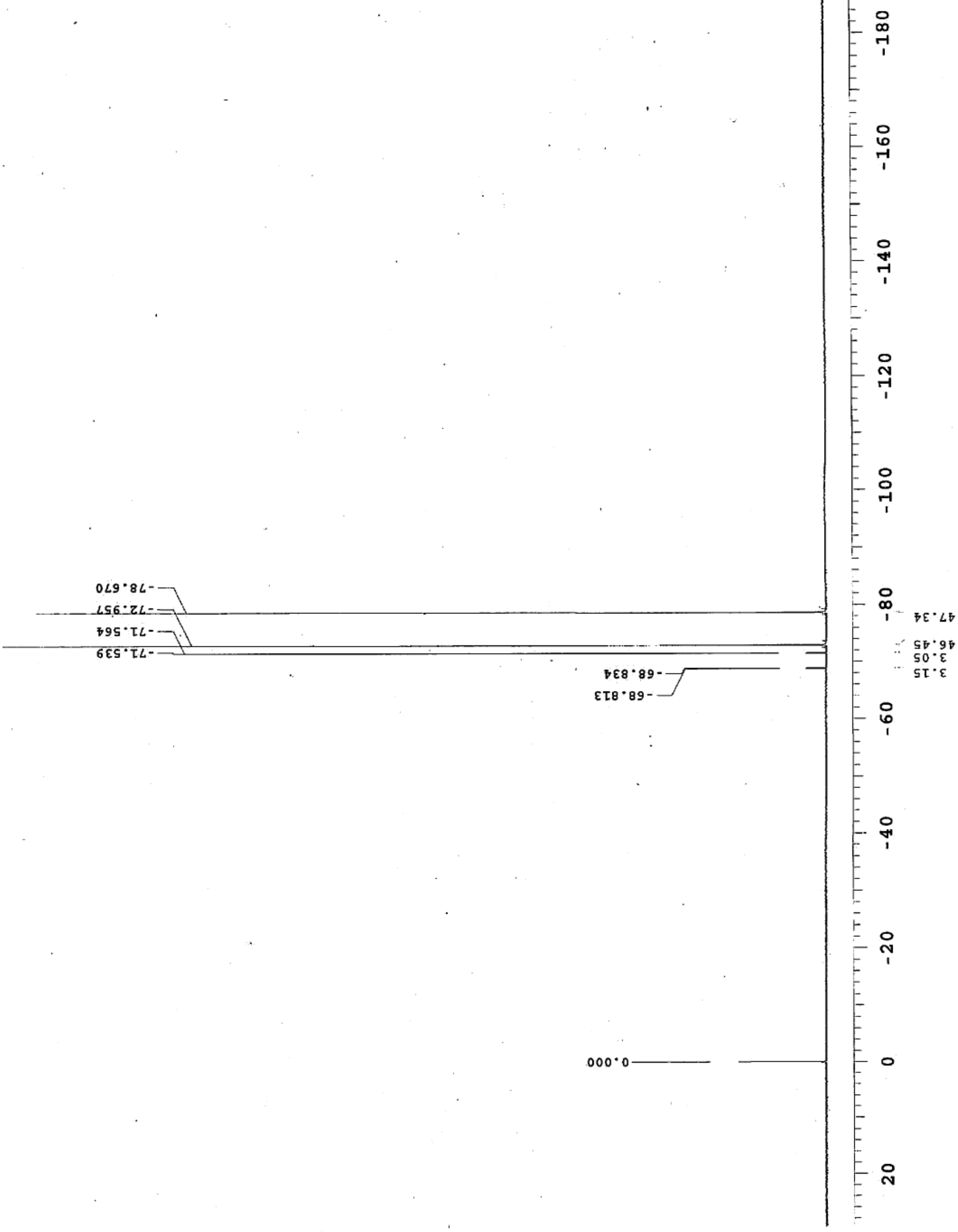

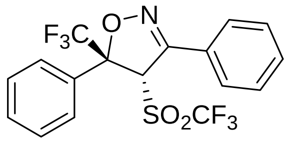

(4S*,5R*)-**1a**
(dr = 94:6)
^{13}C NMR

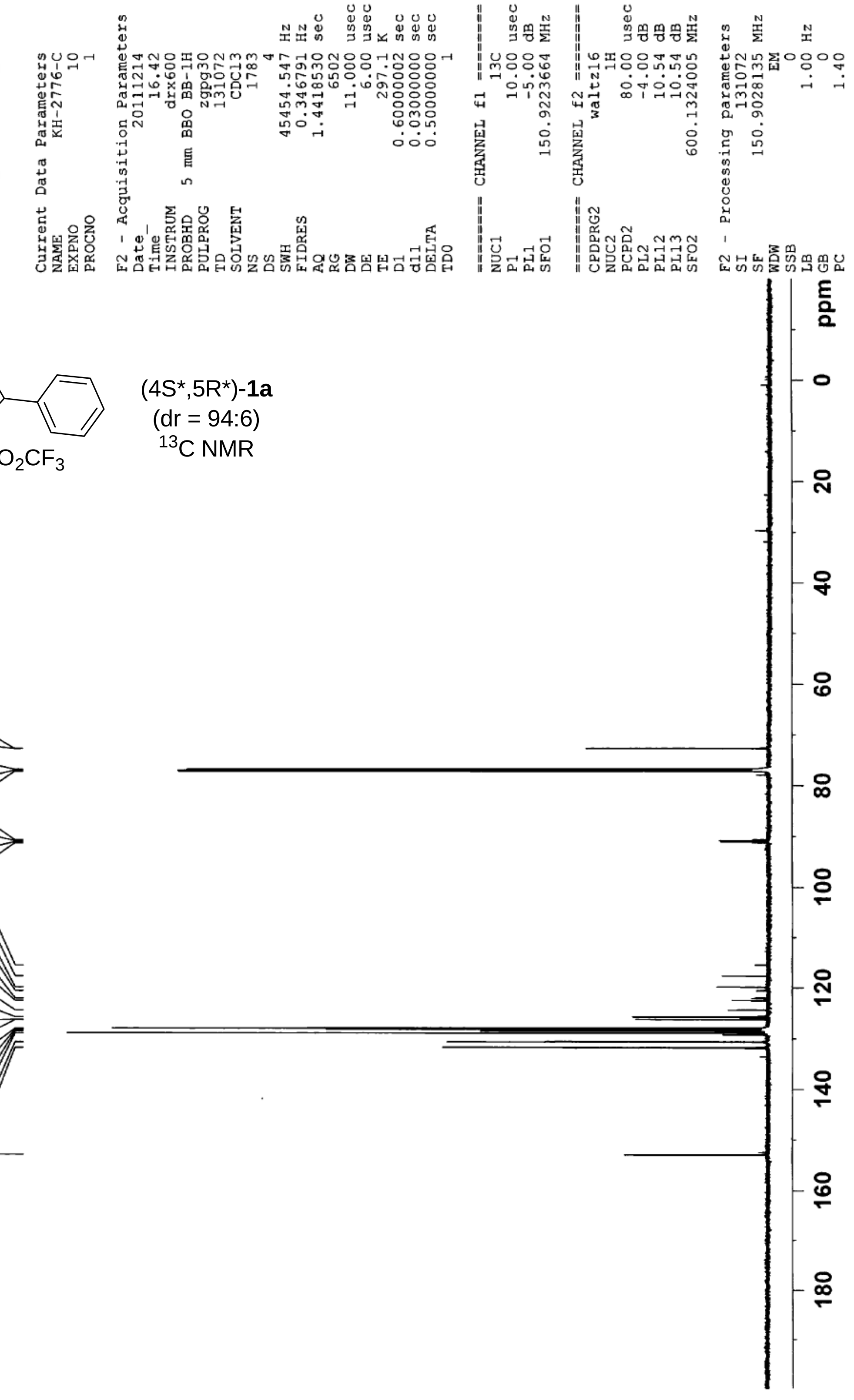

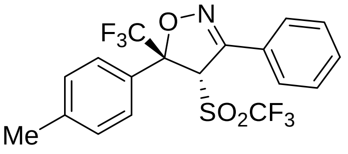

(4S*,5R*)-**1b**
(dr = 93:7)
^{1}H NMR

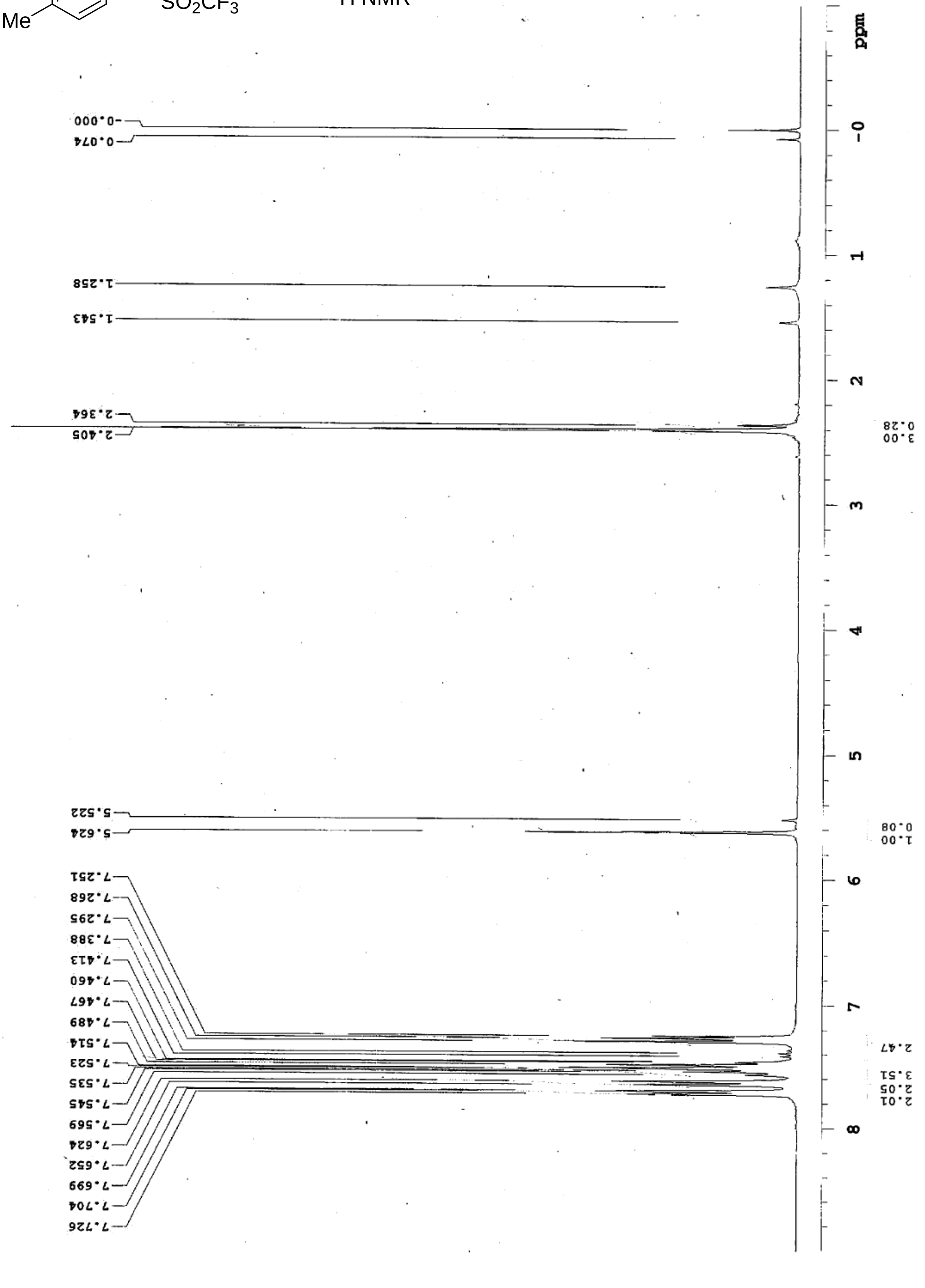

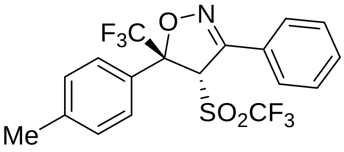

(4S*,5R*)-**1b**
(dr = 93:7)
^{19}F NMR

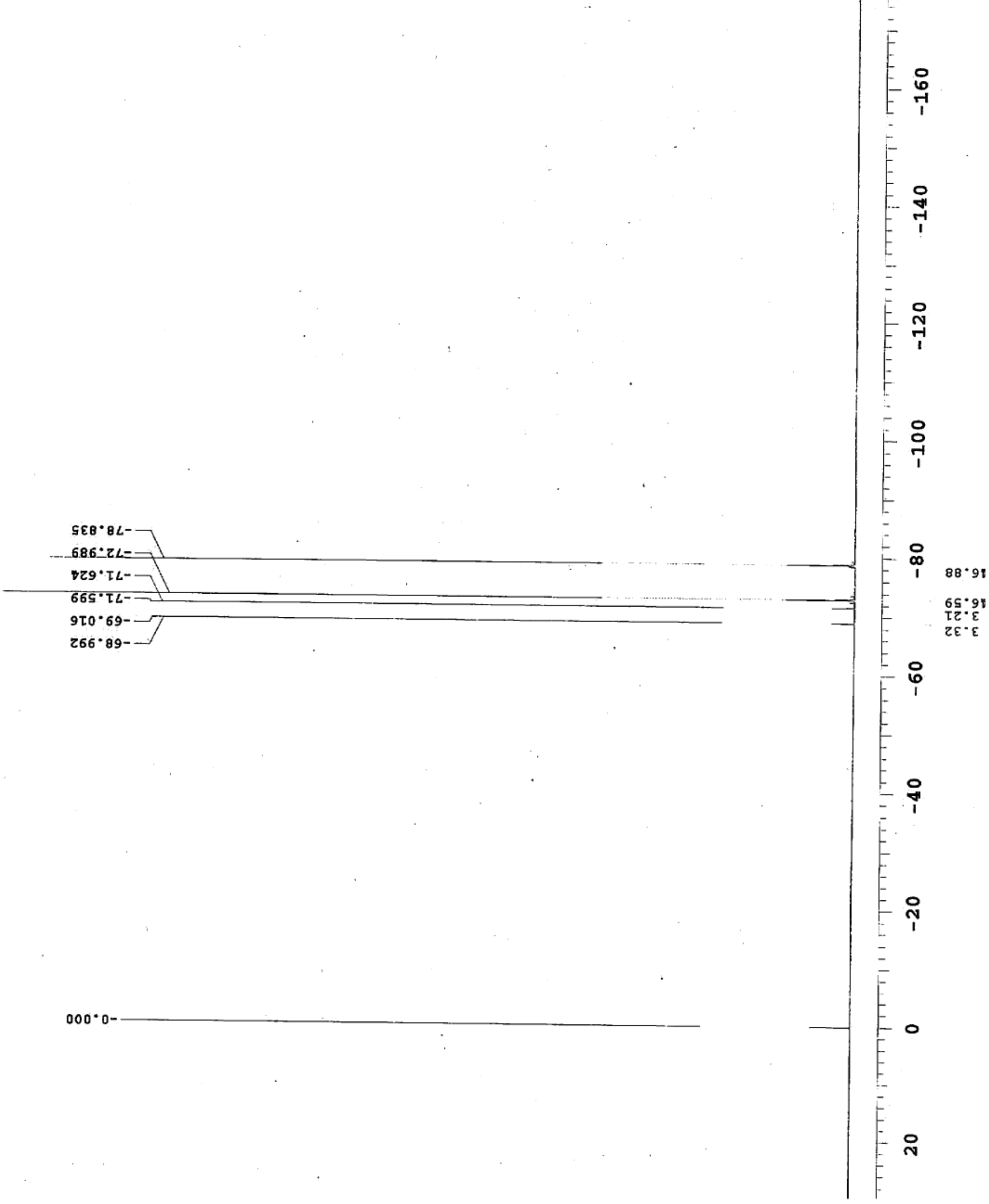

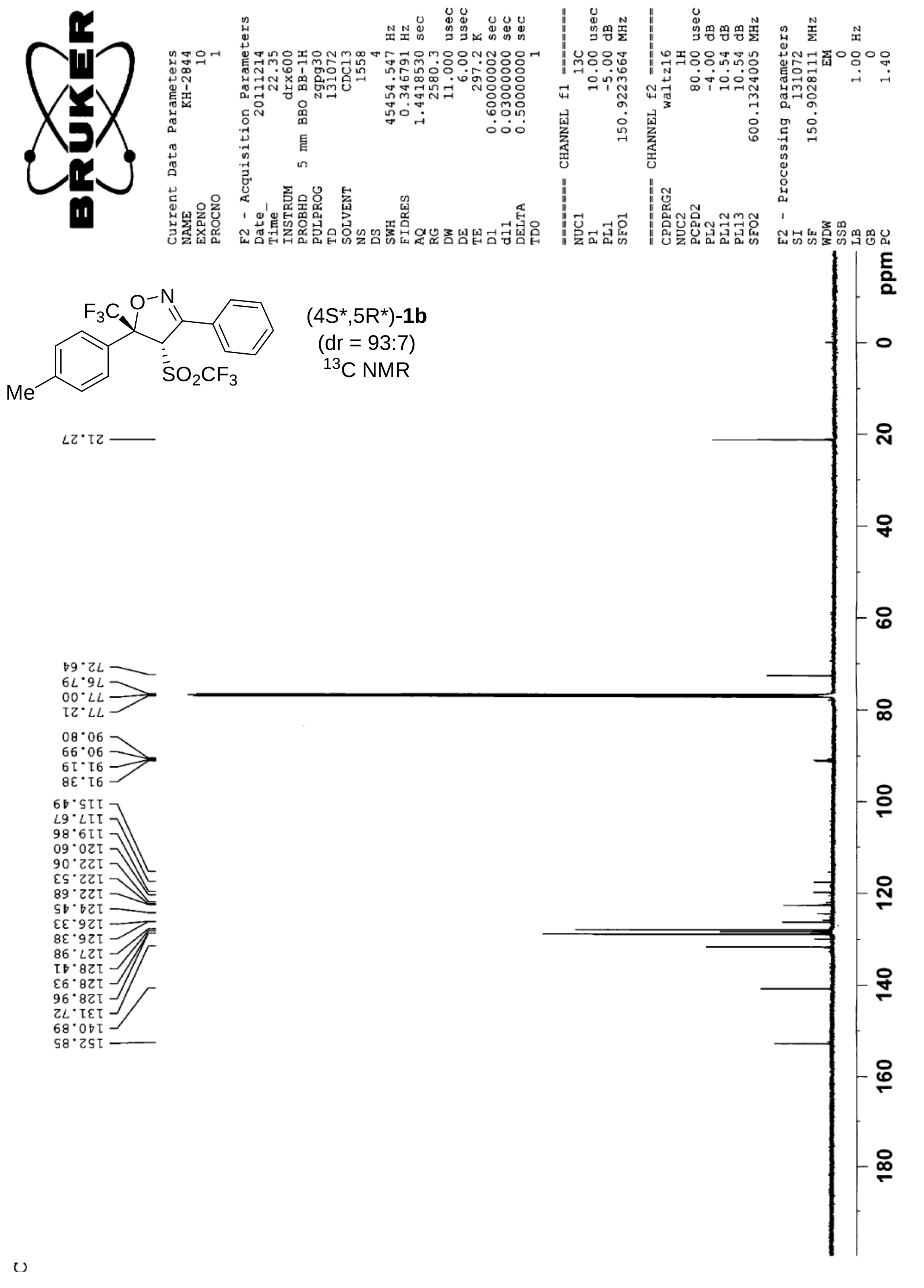
(4S*,5R*)-1b
(dr = 93:7)
13C NMR

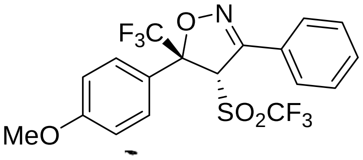

(4S*,5R*)-**1c**
(dr = 95:5)
^{1}H NMR

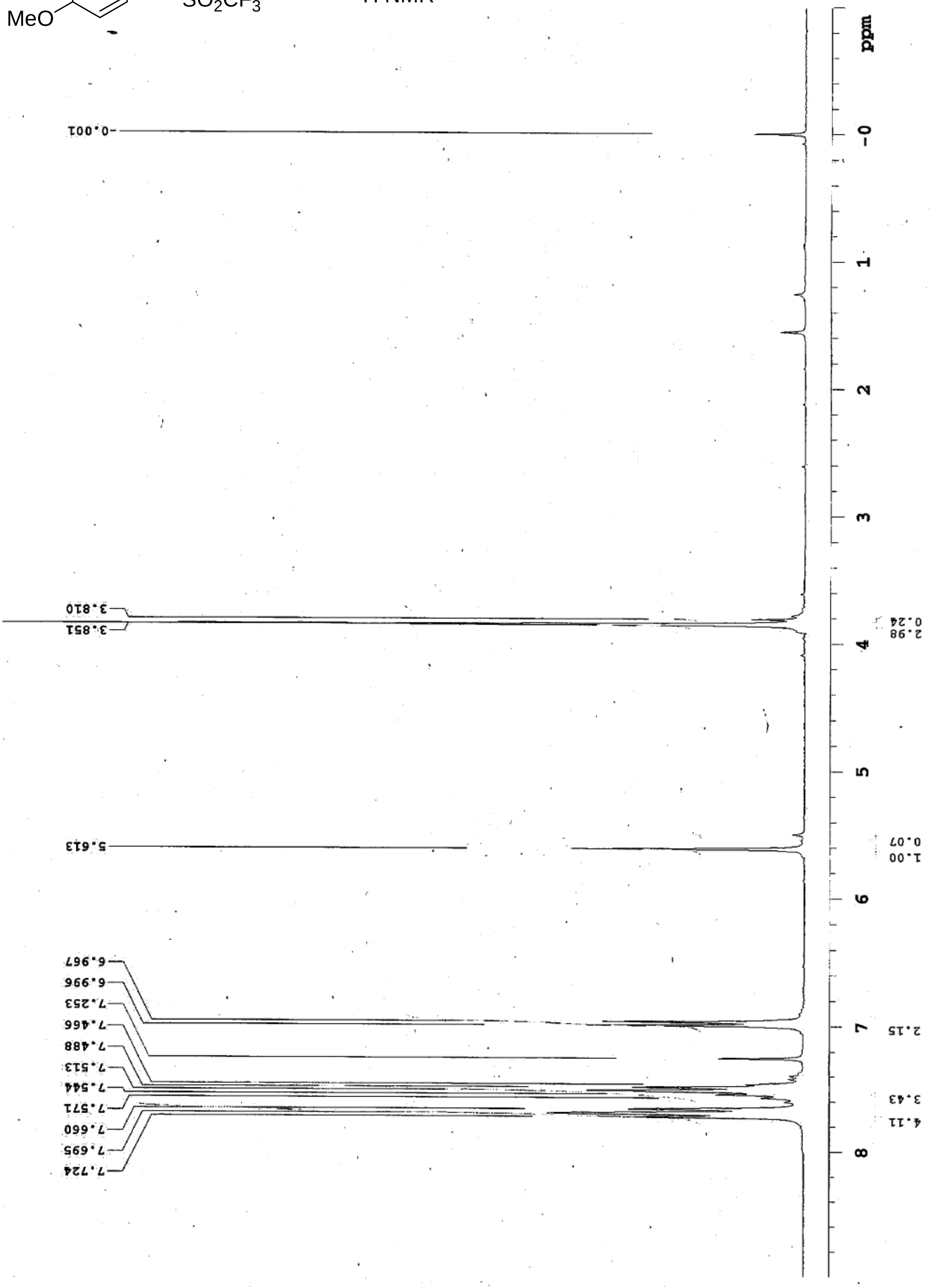

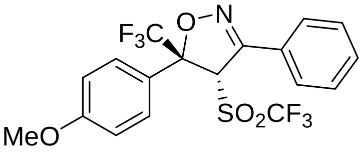

(4S*,5R*)-**1c**
(dr = 95:5)
^{19}F NMR

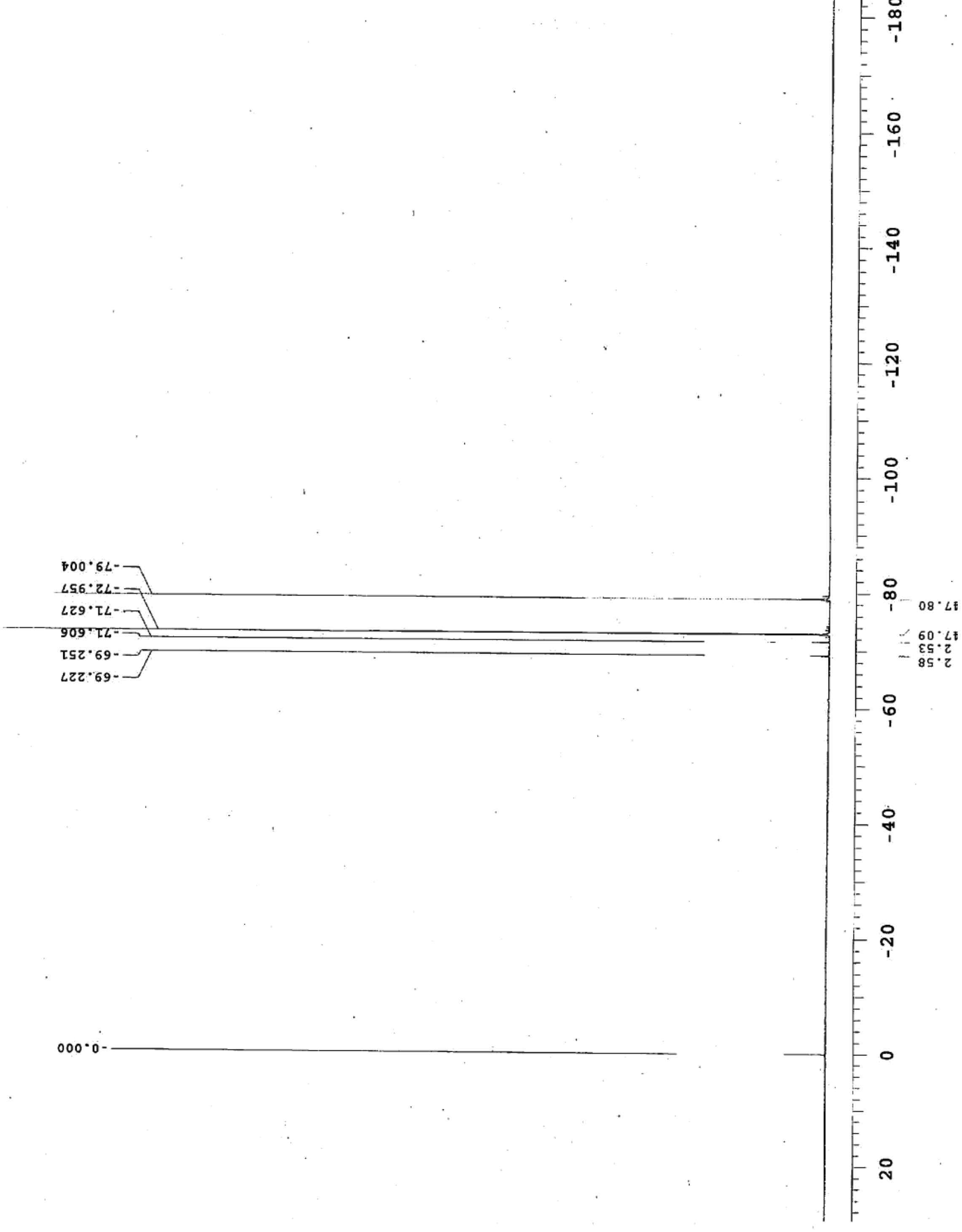

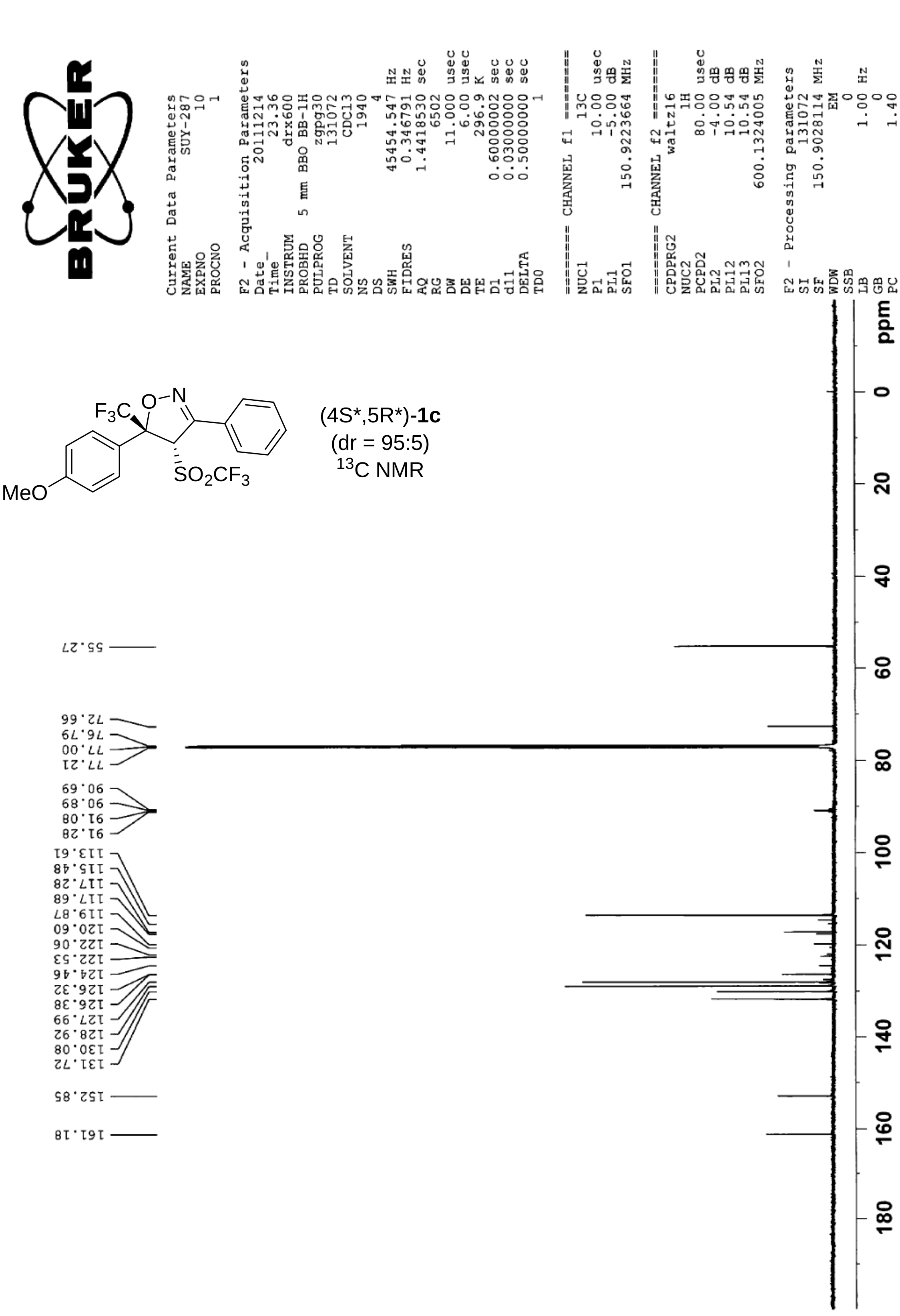

(4S*,5R*)-1c
(dr = 95:5)
13C NMR
MeO
F3C
O–N
SO2CF3
BRUKER
Current Data Parameters
NAME SUY-287
EXPNO 10
PROCNO 1
F2 - Acquisition Parameters
Date_ 20111214
Time 23.36
INSTRUM drx600
PROBHD 5 mm BBO BB-1H
PULPROG zgpg30
TD 131072
SOLVENT CDCl3
NS 1940
DS 4
SWH 45454.547 Hz
FIDRES 0.346791 Hz
AQ 1.4418530 sec
RG 6502
DW 11.000 usec
DE 6.00 usec
TE 296.9 K
D1 0.60000002 sec
d11 0.03000000 sec
DELTA 0.50000000 sec
TD0 1
======== CHANNEL f1 ========
NUC1 13C
P1 10.00 usec
PL1 -5.00 dB
SFO1 150.9223664 MHz
======== CHANNEL f2 ========
CPDPRG2 waltz16
NUC2 1H
PCPD2 80.00 usec
PL2 -4.00 dB
PL12 10.54 dB
PL13 10.54 dB
SFO2 600.1324005 MHz
F2 - Processing parameters
SI 131072
SF 150.9028114 MHz
WDW EM
SSB 0
LB 1.00 Hz
GB 0
PC 1.40
55.27
72.66
76.79
77.00
77.21
90.69
90.89
91.08
91.28
113.61
115.48
117.28
117.68
119.87
120.60
122.06
122.53
124.46
126.32
126.38
127.99
128.92
130.08
131.72
152.85
161.18
ppm
0
20
40
60
80
100
120
140
160
180

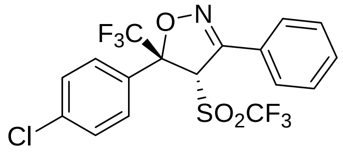

(4S*,5R*)-**1d**
(dr = 97:3)
^{1}H NMR

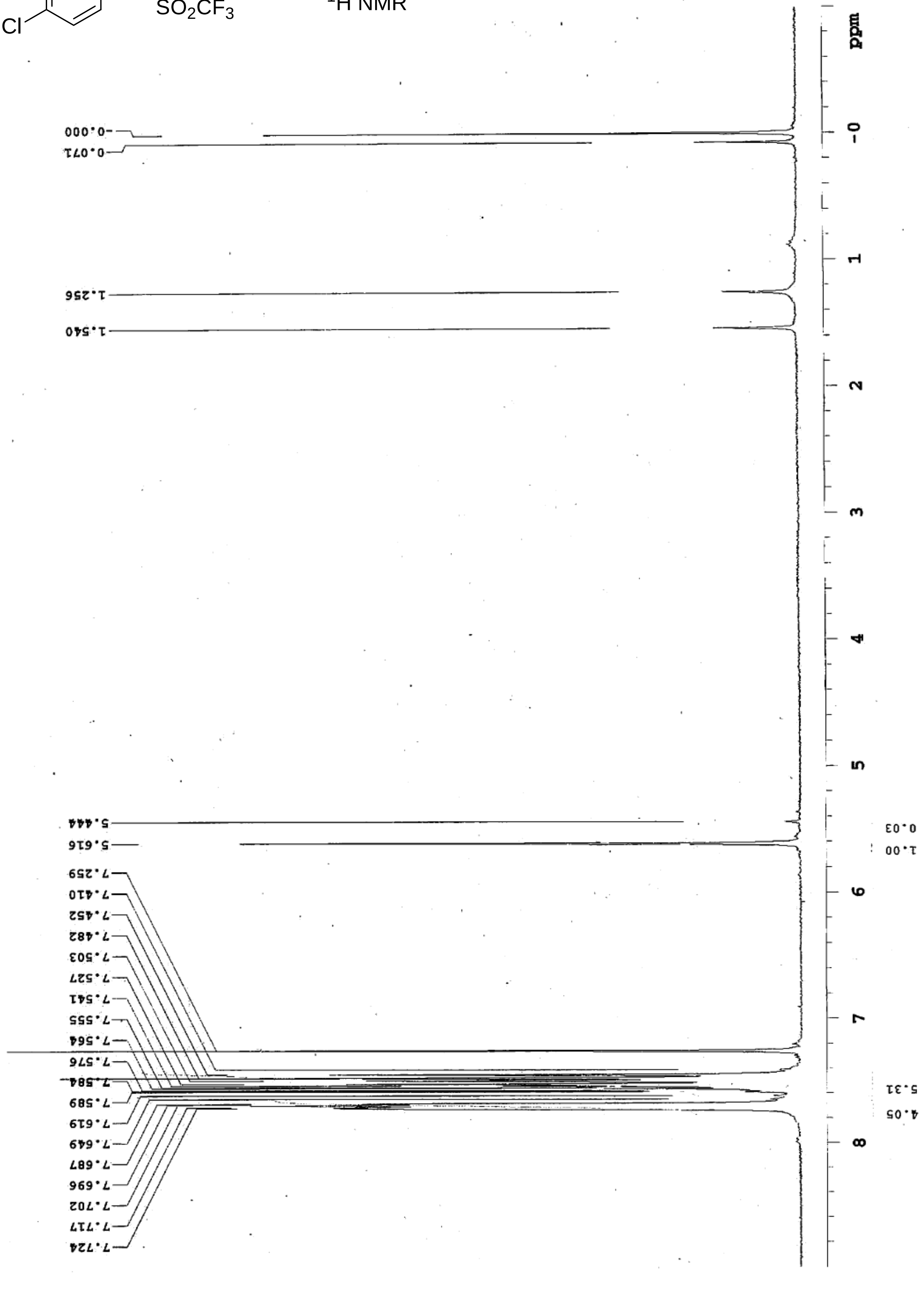

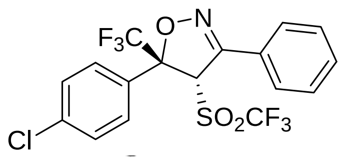

(4S*,5R*)-**1d**
(dr = 97:3)
^{19}F NMR

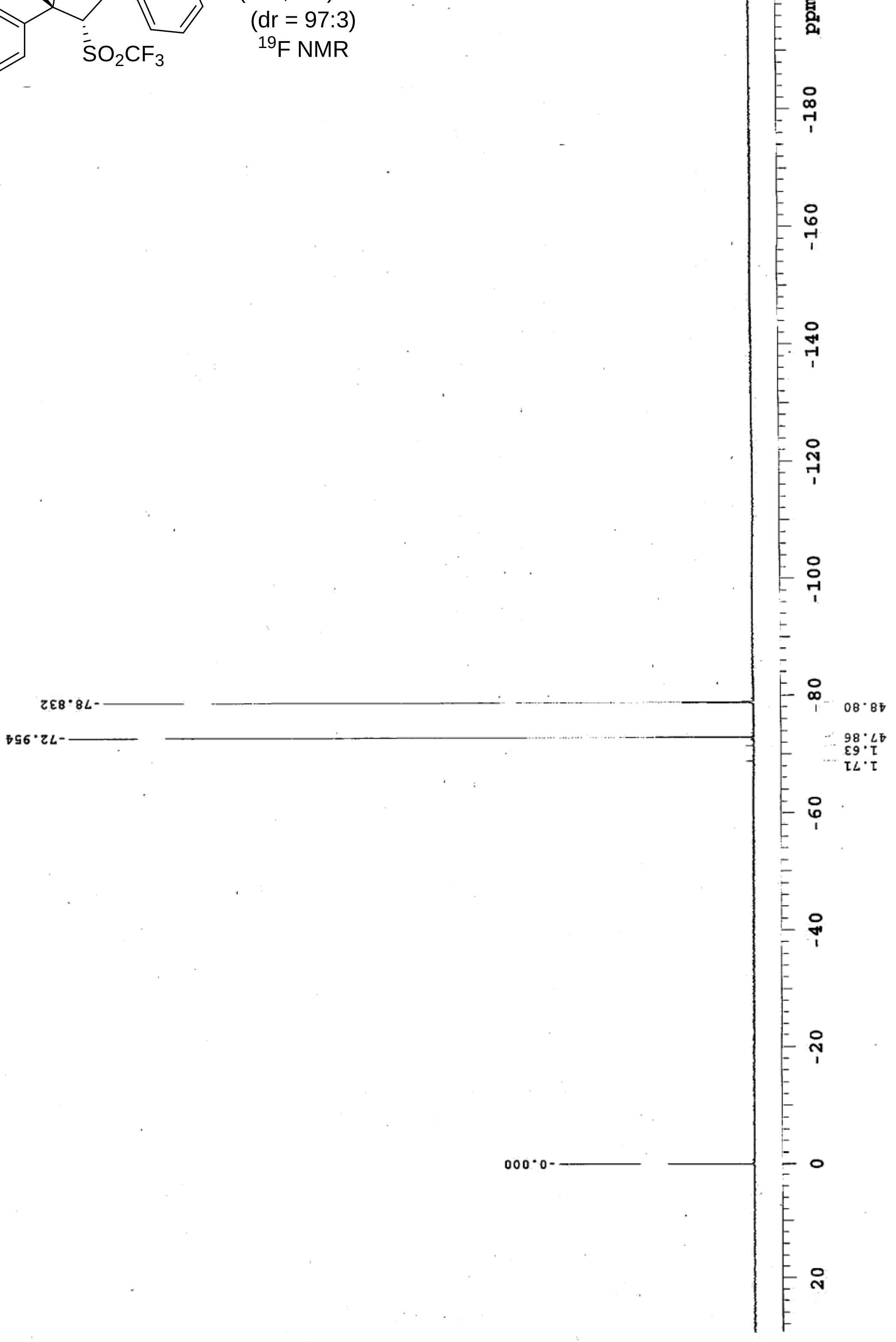

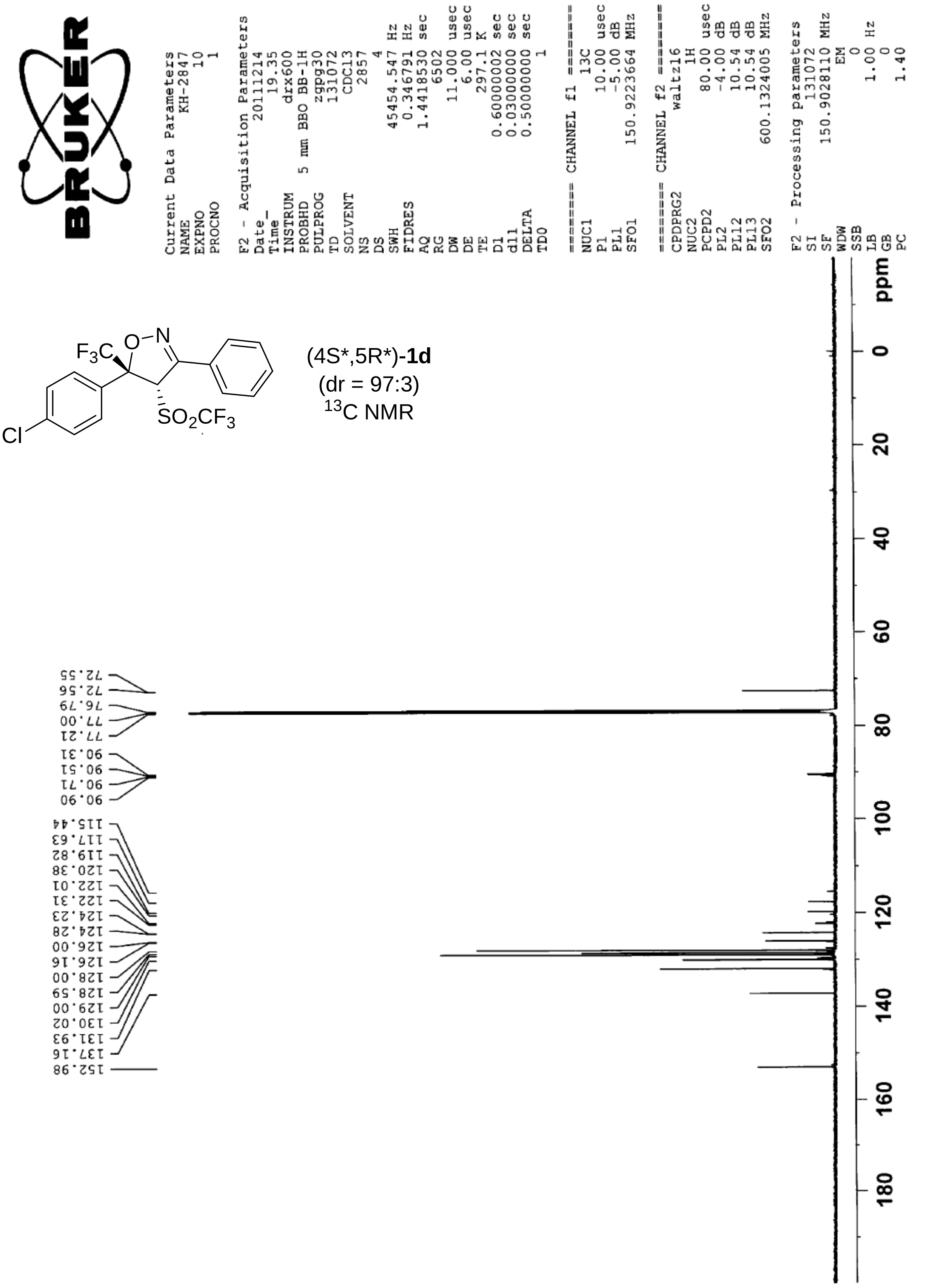
(4S*,5R*)-1d
(dr = 97:3)
13C NMR
F3C
O–N
Cl
SO2CF3
BRUKER
Current Data Parameters
NAME KH-2847
EXPNO 10
PROCNO 1
F2 - Acquisition Parameters
Date_ 20111214
Time_ 19.35
INSTRUM drx600
PROBHD 5 mm BBO BB-1H
PULPROG zgpg30
TD 131072
SOLVENT CDCl3
NS 2857
DS 4
SWH 45454.547 Hz
FIDRES 0.346791 Hz
AQ 1.4418530 sec
RG 6502
DW 11.000 usec
DE 6.00 usec
TE 297.1 K
D1 0.60000002 sec
d11 0.03000000 sec
DELTA 0.50000000 sec
TD0 1
======== CHANNEL f1 ========
NUC1 13C
P1 10.00 usec
PL1 -5.00 dB
SFO1 150.9223664 MHz
======== CHANNEL f2 ========
CPDPRG2 waltz16
NUC2 1H
PCPD2 80.00 usec
PL2 -4.00 dB
PL12 10.54 dB
PL13 10.54 dB
SFO2 600.1324005 MHz
F2 - Processing parameters
SI 131072
SF 150.9028110 MHz
WDW EM
SSB 0
LB 1.00 Hz
GB 0
PC 1.40
72.55
72.56
76.79
77.00
77.21
90.31
90.51
90.71
90.90
115.44
117.63
119.82
120.38
122.01
122.31
124.23
124.28
126.00
126.16
128.00
128.59
129.00
130.02
131.93
137.16
152.98
ppm
0
20
40
60
80
100
120
140
160
180

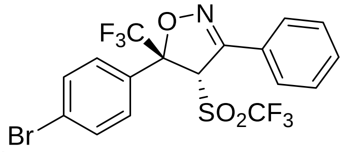

(4S*,5R*)-**1e**
(dr = 96:4)
^{1}H NMR

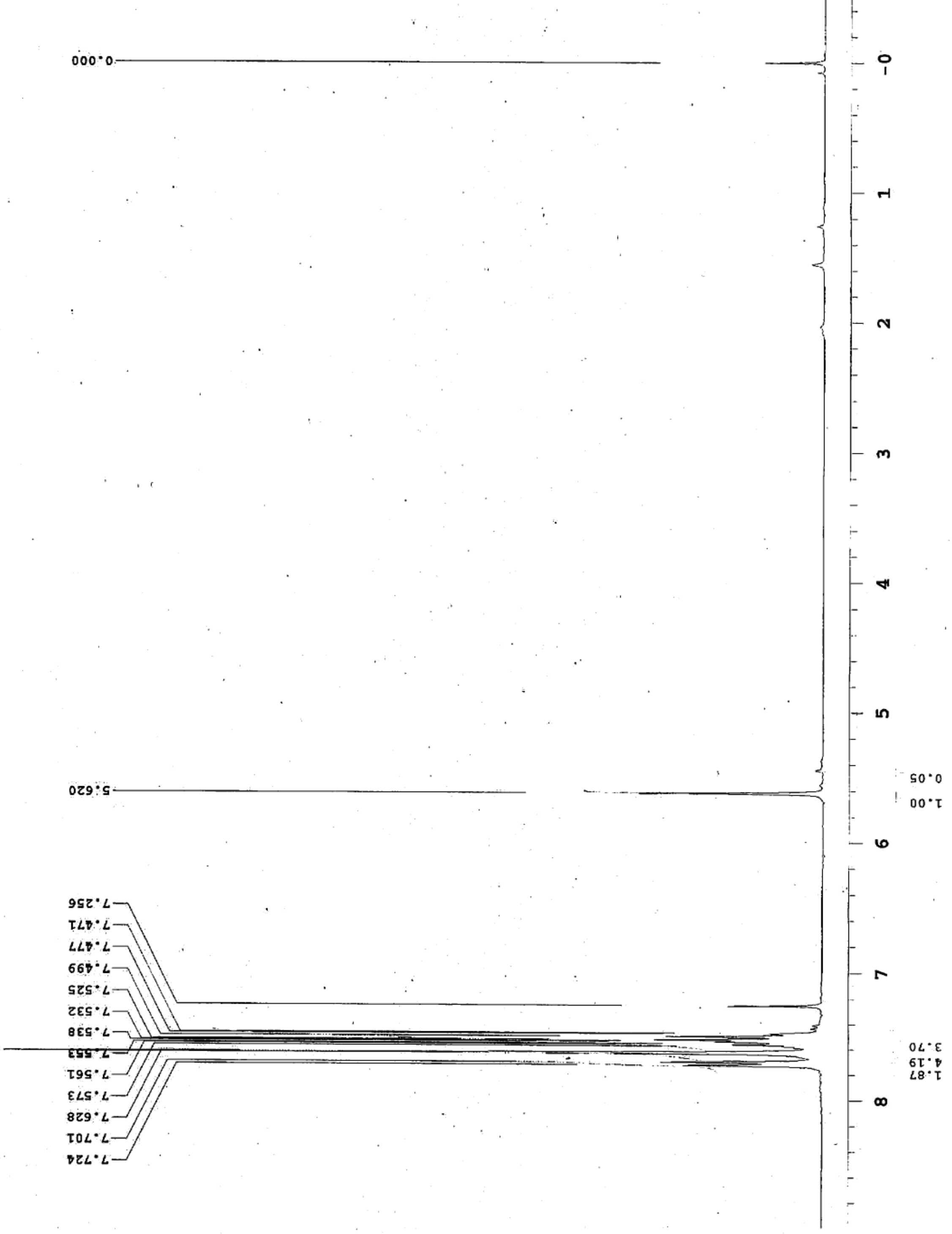

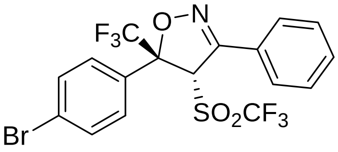

(4S*,5R*)-**1e**
(dr = 96:4)
^{19}F NMR

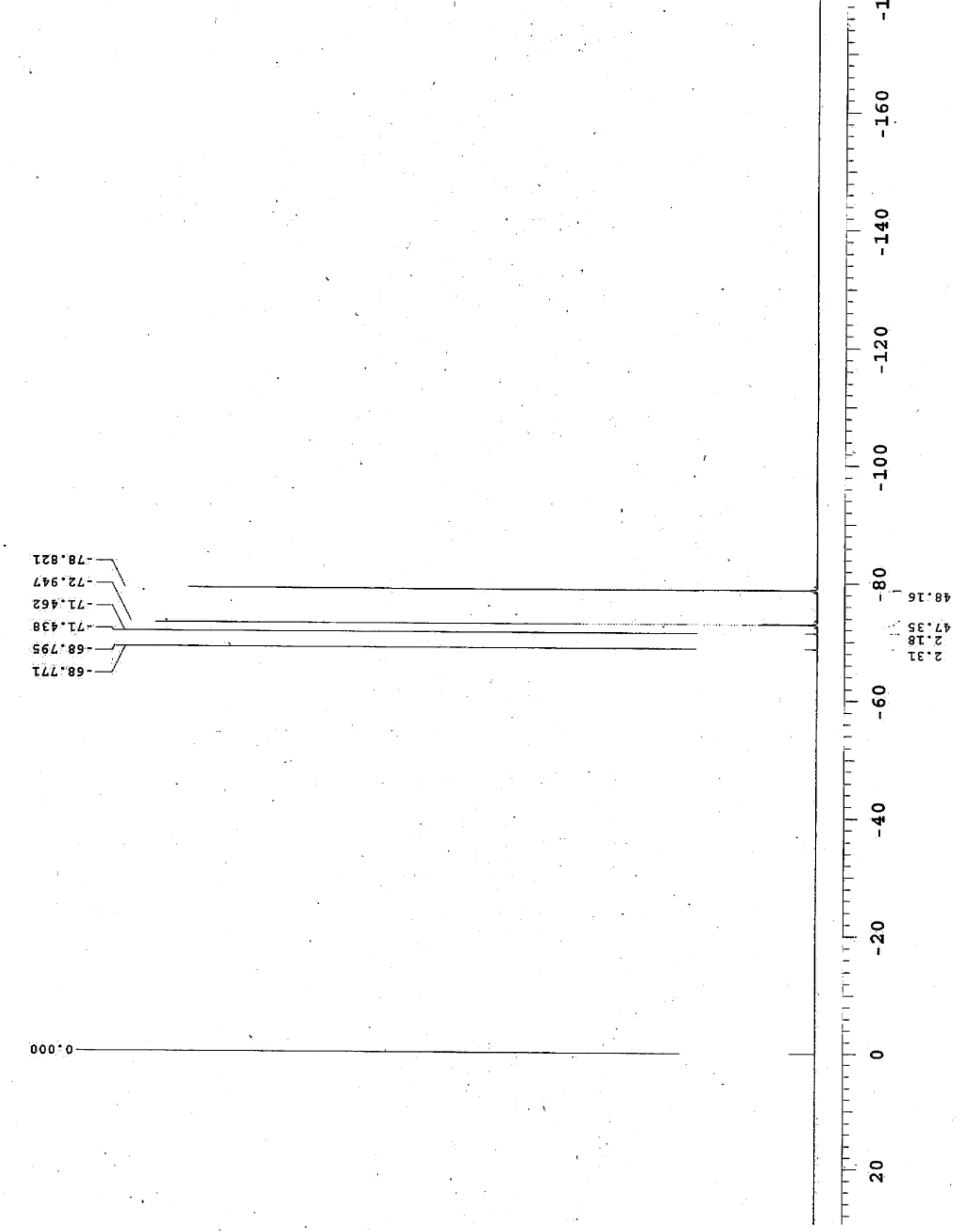

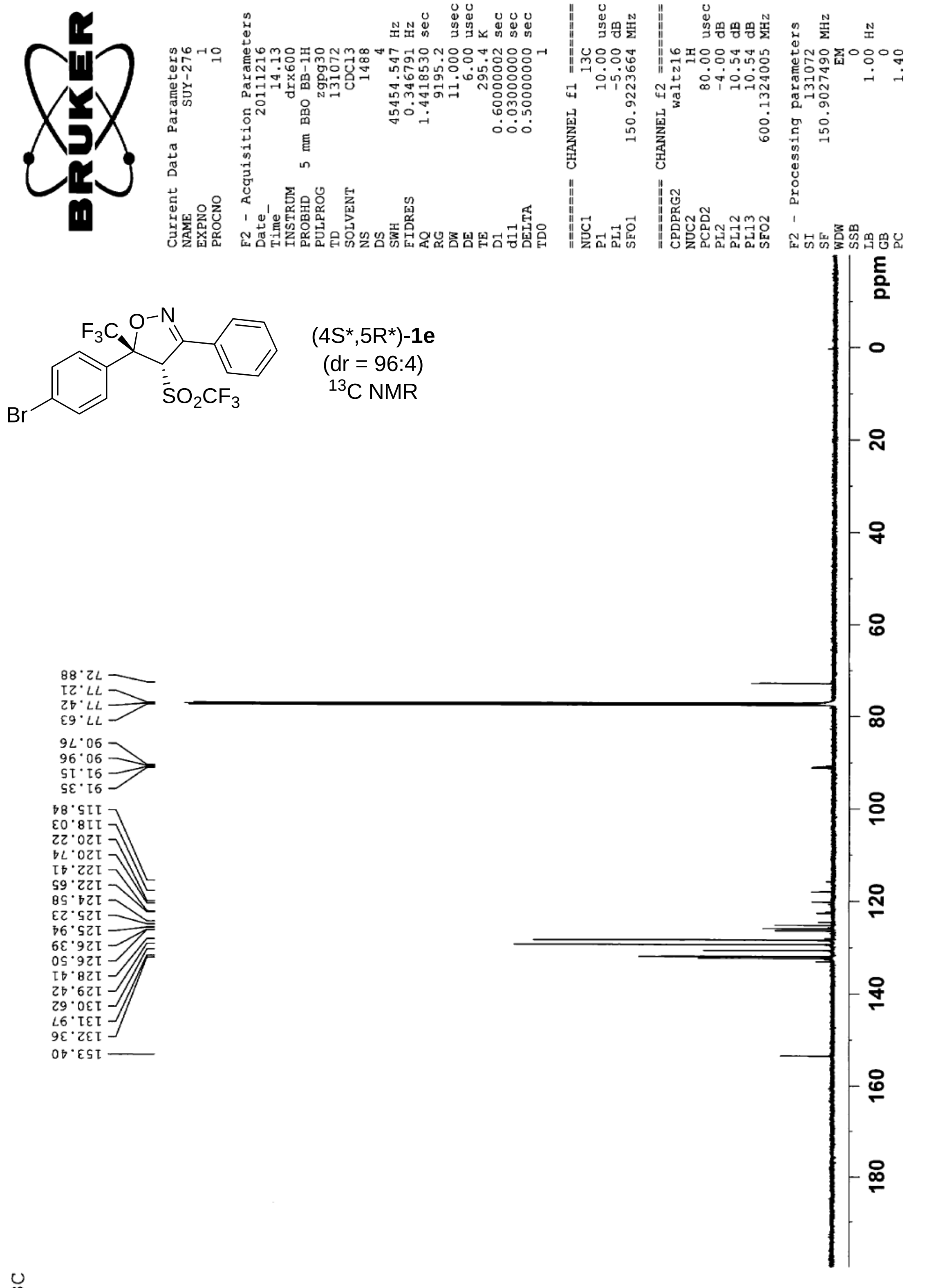
(4S*,5R*)-1e
(dr = 96:4)
13C NMR
Br
F3C
O–N
SO2CF3
72.88
77.21
77.42
77.63
90.76
90.96
91.15
91.35
115.84
118.03
120.22
120.74
122.41
122.65
124.58
125.23
125.94
126.39
126.50
128.41
129.42
130.62
131.97
132.36
153.40
ppm
BRUKER
Current Data Parameters
NAME SUY-276
EXPNO 1
PROCNO 10
F2 - Acquisition Parameters
Date_ 20111216
Time_ 14.13
INSTRUM drx600
PROBHD 5 mm BBO BB-1H
PULPROG zgpg30
TD 131072
SOLVENT CDCl3
NS 1488
DS 4
SWH 45454.547 Hz
FIDRES 0.346791 Hz
AQ 1.4418530 sec
RG 9195.2
DW 11.000 usec
DE 6.00 usec
TE 295.4 K
D1 0.60000002 sec
d11 0.03000000 sec
DELTA 0.50000000 sec
TD0 1
======== CHANNEL f1 ========
NUC1 13C
P1 10.00 usec
PL1 -5.00 dB
SFO1 150.9223664 MHz
======== CHANNEL f2 ========
CPDPRG2 waltz16
NUC2 1H
PCPD2 80.00 usec
PL2 -4.00 dB
PL12 10.54 dB
PL13 10.54 dB
SFO2 600.1324005 MHz
F2 - Processing parameters
SI 131072
SF 150.9027490 MHz
WDW EM
SSB 0
LB 1.00 Hz
GB 0
PC 1.40

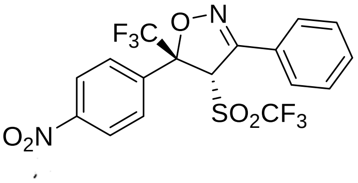

(4S*,5R*)-**1f**
(dr = 97:3)
^{1}H NMR

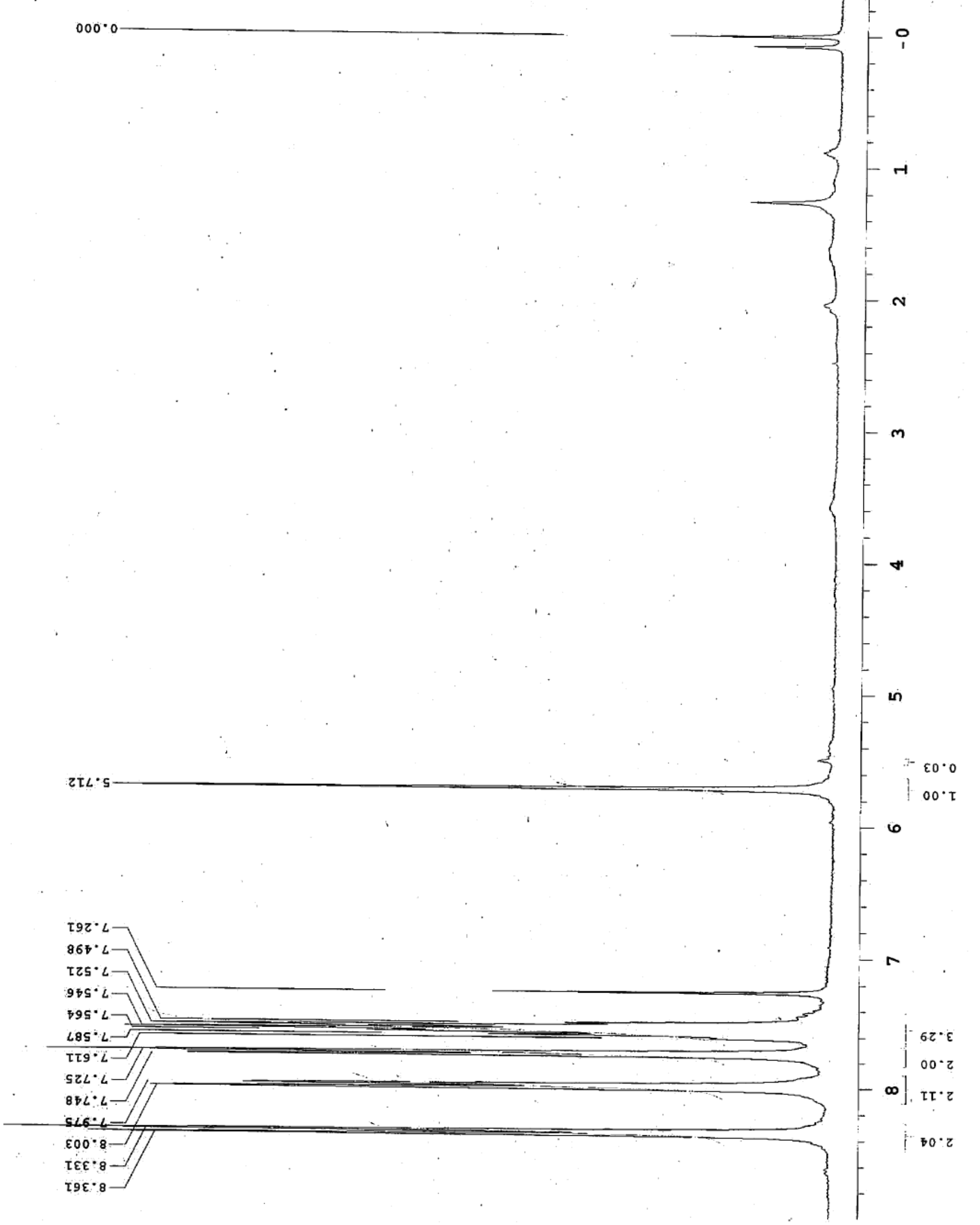

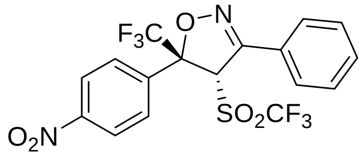

(4S*,5R*)-**1f**
(dr = 97:3)
^{19}F NMR

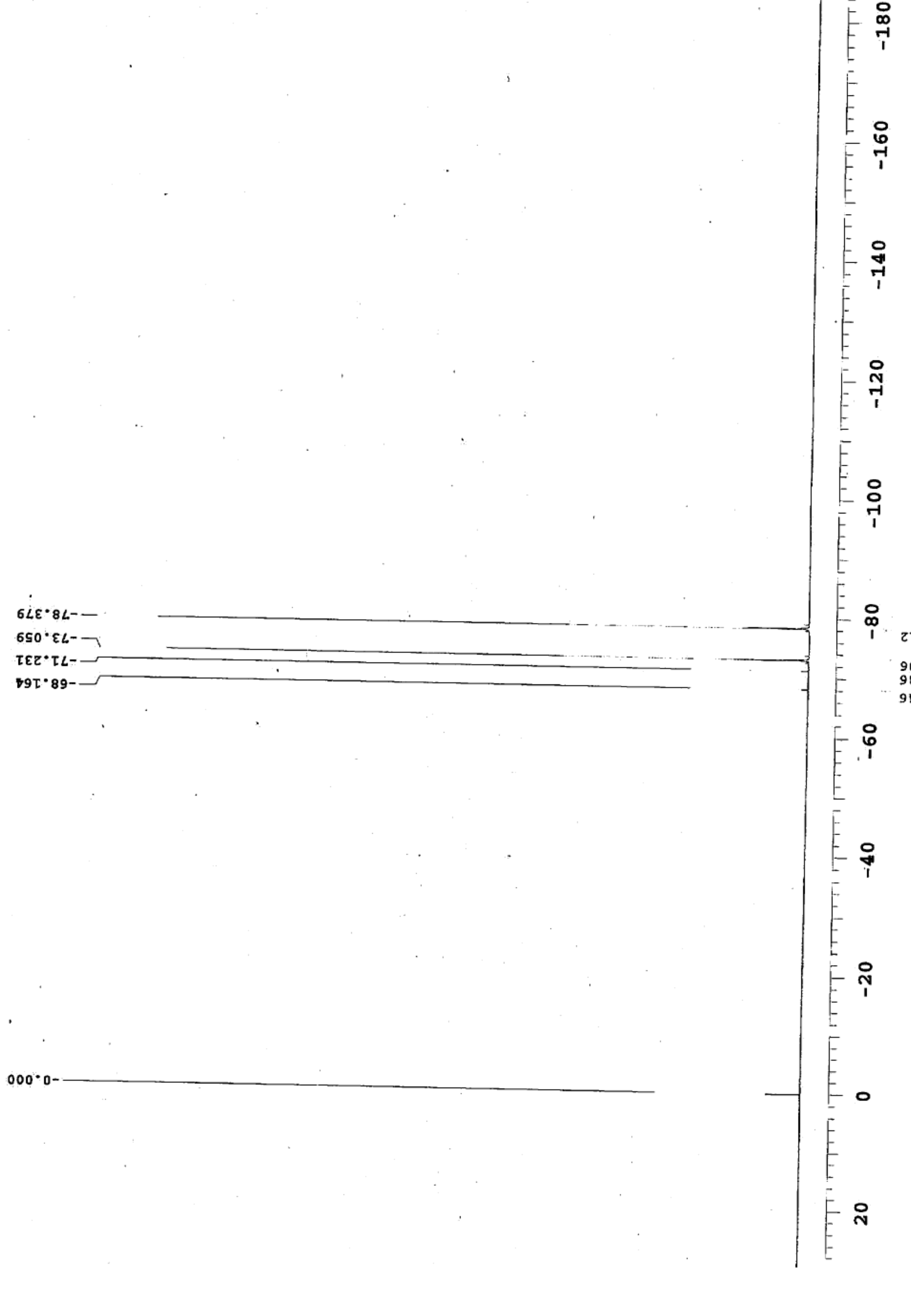

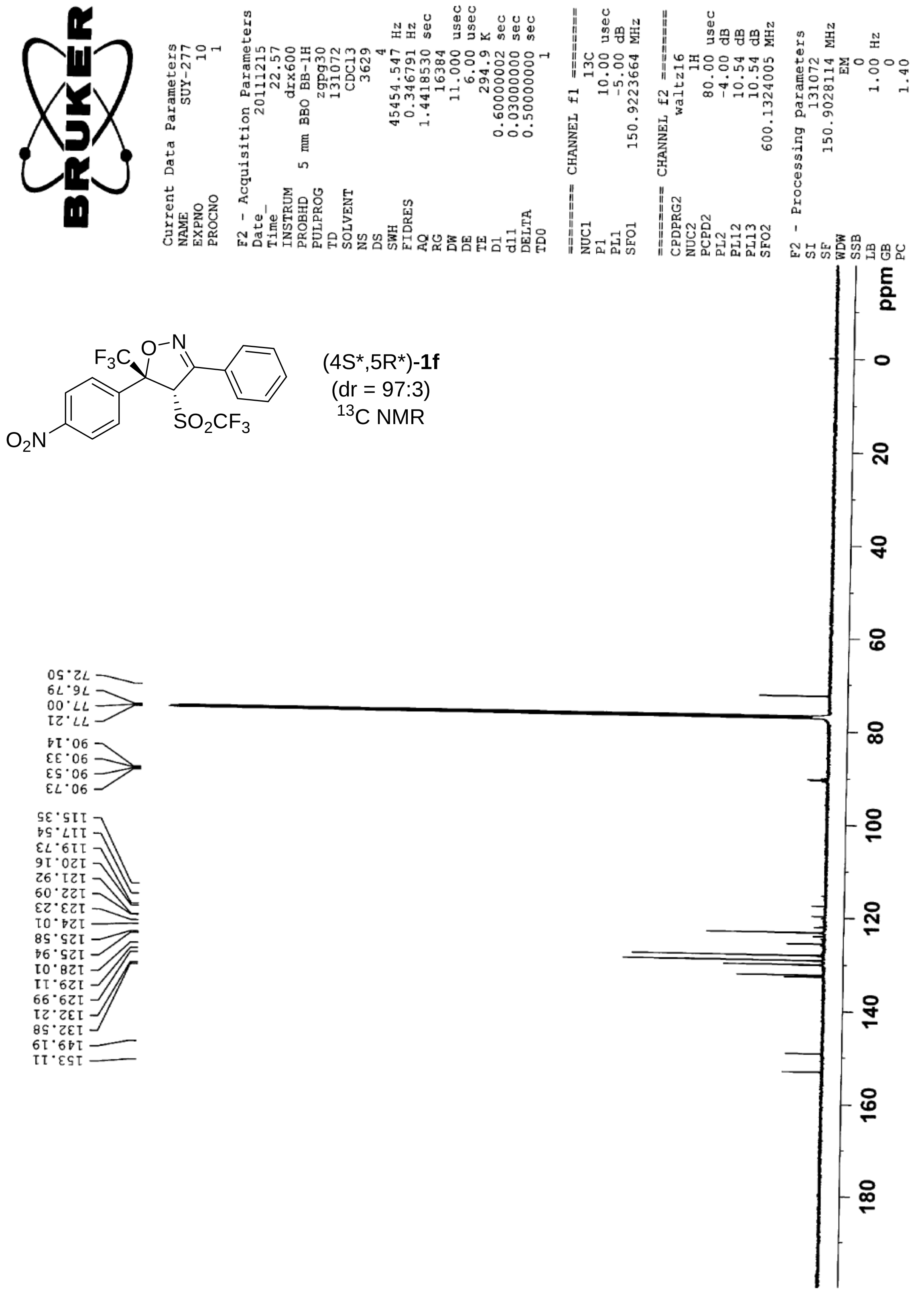
(4S*,5R*)-1f
(dr = 97:3)
13C NMR
F3C
O
N
SO2CF3
O2N
153.11
149.19
132.58
132.21
129.99
129.11
128.01
125.94
125.58
124.01
123.23
122.09
121.92
120.16
119.73
117.54
115.35
90.73
90.53
90.33
90.14
77.21
77.00
76.79
72.50
BRUKER
Current Data Parameters
NAME SUY-277
EXPNO 10
PROCNO 1
F2 - Acquisition Parameters
Date_ 20111215
Time 22.57
INSTRUM drx600
PROBHD 5 mm BBO BB-1H
PULPROG zgpg30
TD 131072
SOLVENT CDCl3
NS 3629
DS 4
SWH 45454.547 Hz
FIDRES 0.346791 Hz
AQ 1.4418530 sec
RG 16384
DW 11.000 usec
DE 6.00 usec
TE 294.9 K
D1 0.60000002 sec
d11 0.03000000 sec
DELTA 0.50000000 sec
TD0 1
======== CHANNEL f1 ========
NUC1 13C
P1 10.00 usec
PL1 -5.00 dB
SFO1 150.9223664 MHz
======== CHANNEL f2 ========
CPDPRG2 waltz16
NUC2 1H
PCPD2 80.00 usec
PL2 -4.00 dB
PL12 10.54 dB
PL13 10.54 dB
SFO2 600.1324005 MHz
F2 - Processing parameters
SI 131072
SF 150.9028114 MHz
WDW EM
SSB 0
LB 1.00 Hz
GB 0
PC 1.40
180
160
140
120
100
80
60
40
20
0
ppm

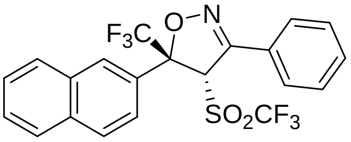

(4S*,5R*)-**1g**
(dr = 96:4)
^{1}H NMR

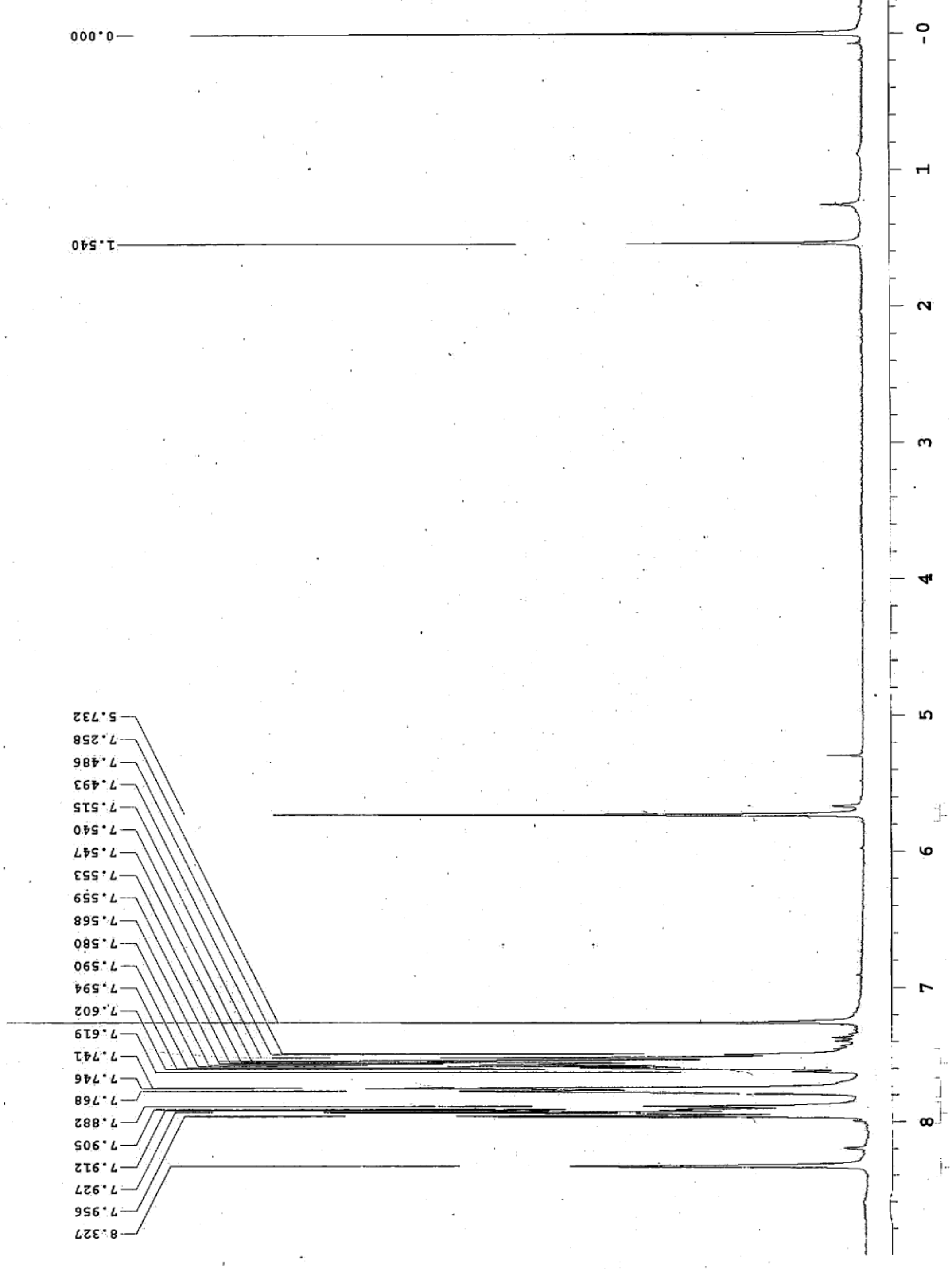

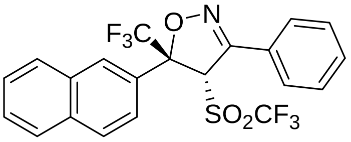

(4S*,5R*)-**1g**
(dr = 96:4)
^{19}F NMR

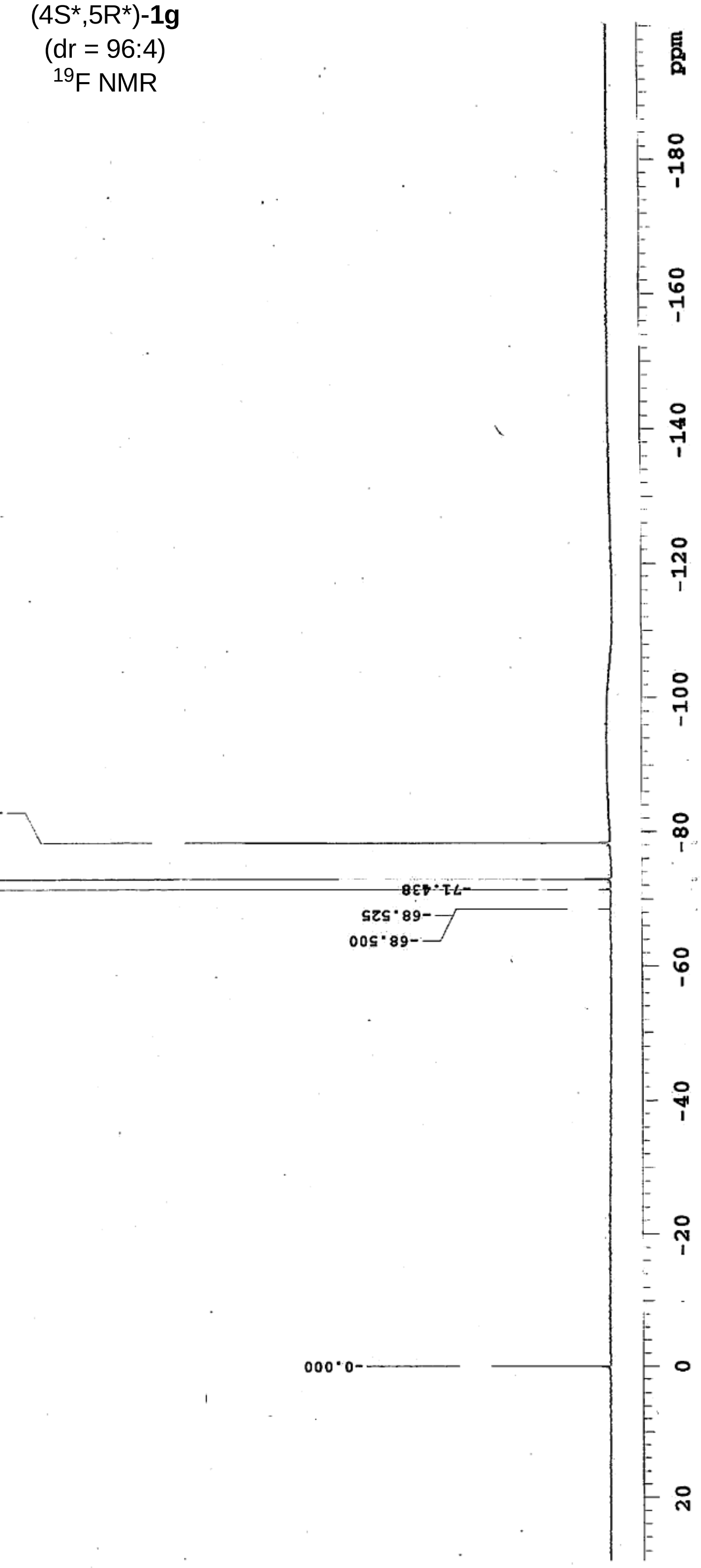

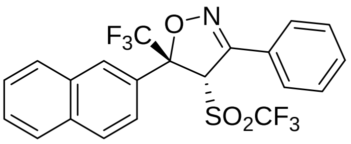

(4S*,5R*)-**1g**
(dr = 96:4)
^{13}C NMR

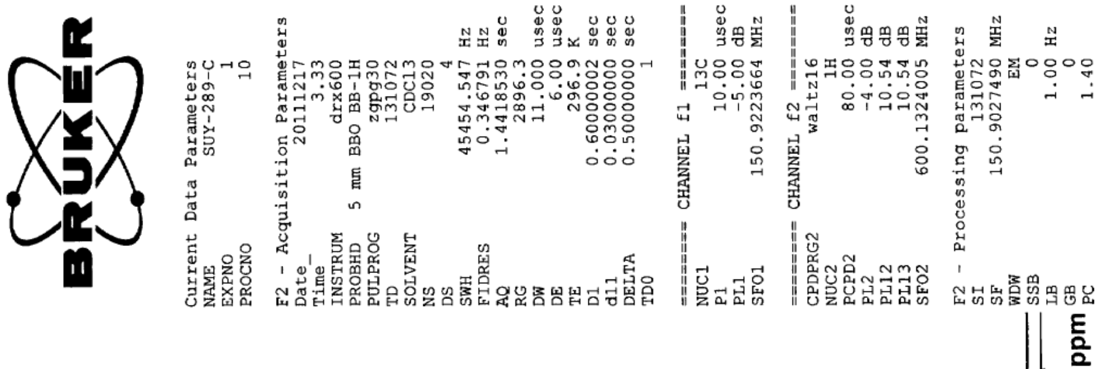
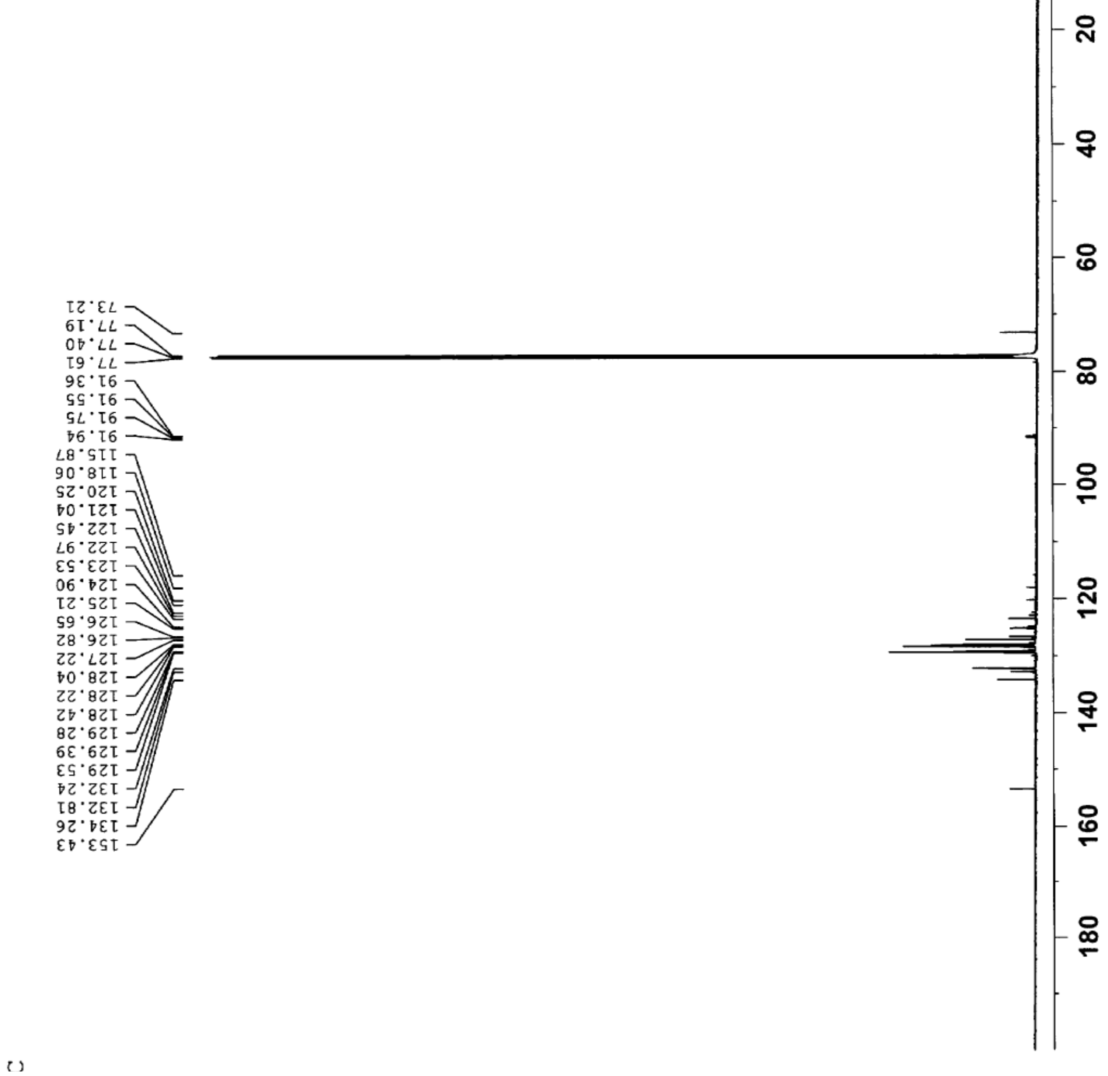

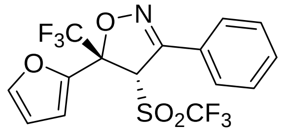

(4S*,5R*)-**1h**
(dr = 99:1)
^{1}H NMR

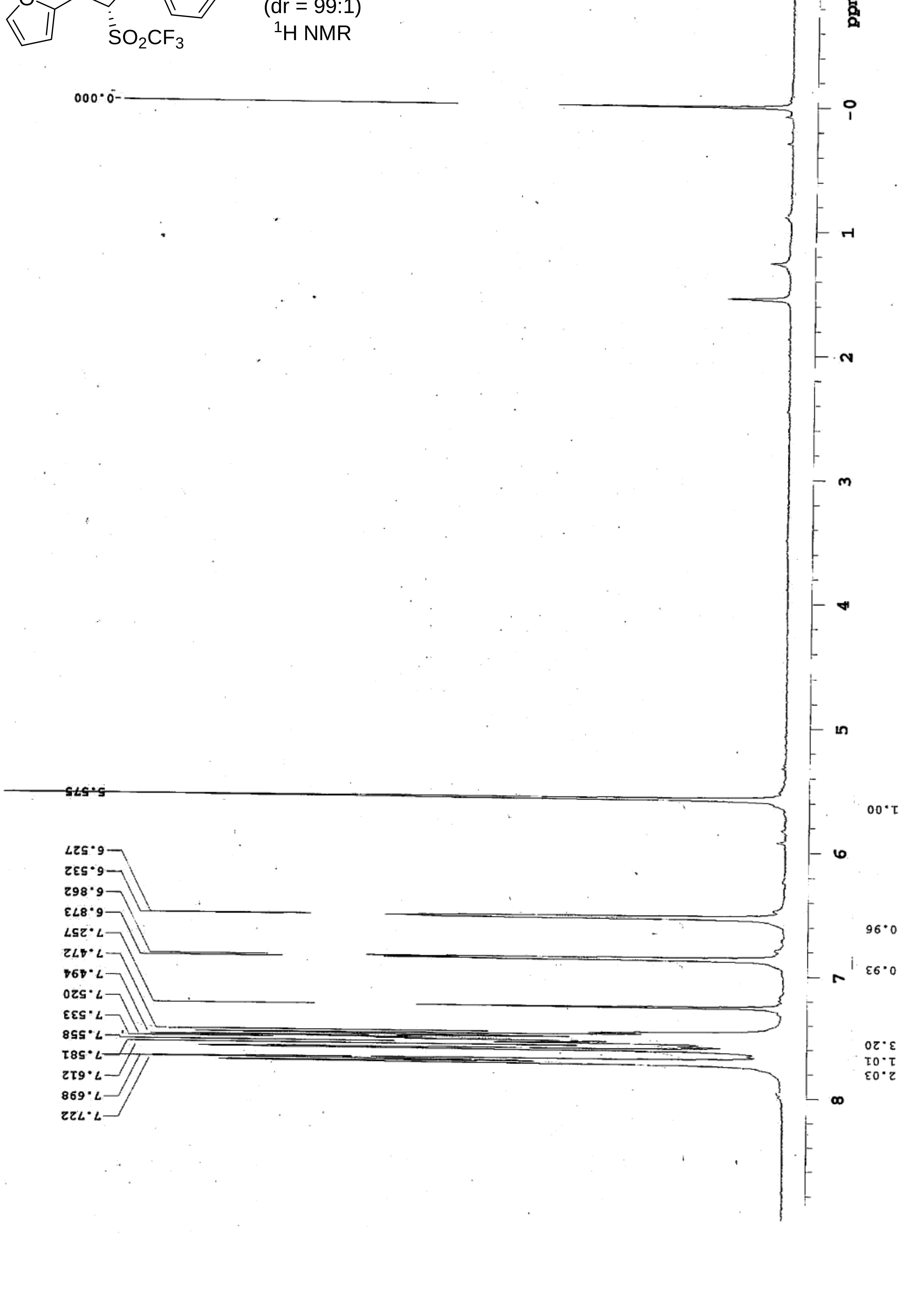

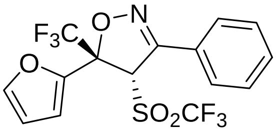

(4S*,5R*)-**1h**
(dr = 99:1)
^{19}F NMR

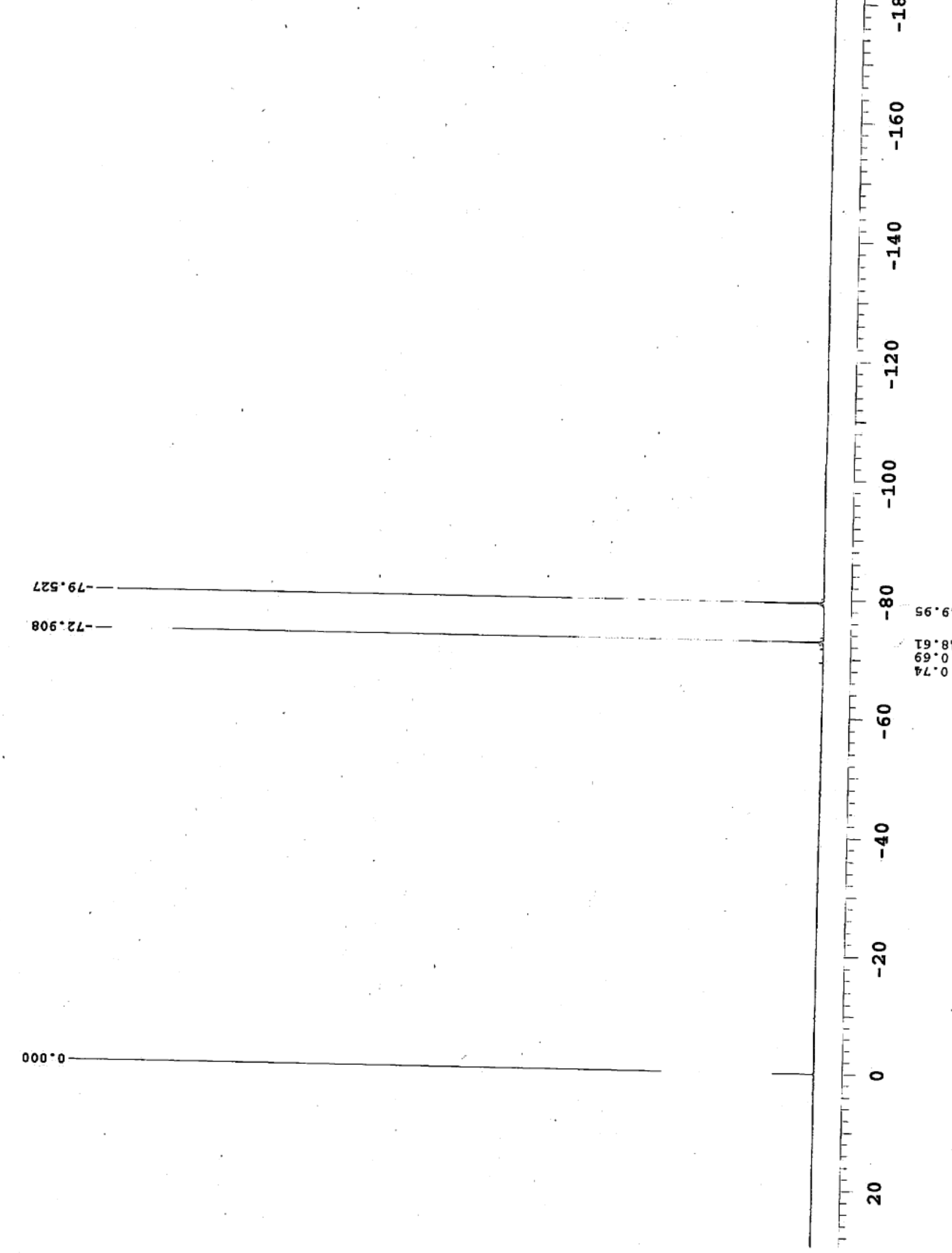

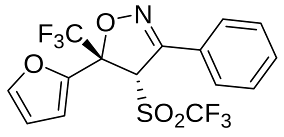

(4S*,5R*)-**1h**
(dr = 99:1)
^{13}C NMR

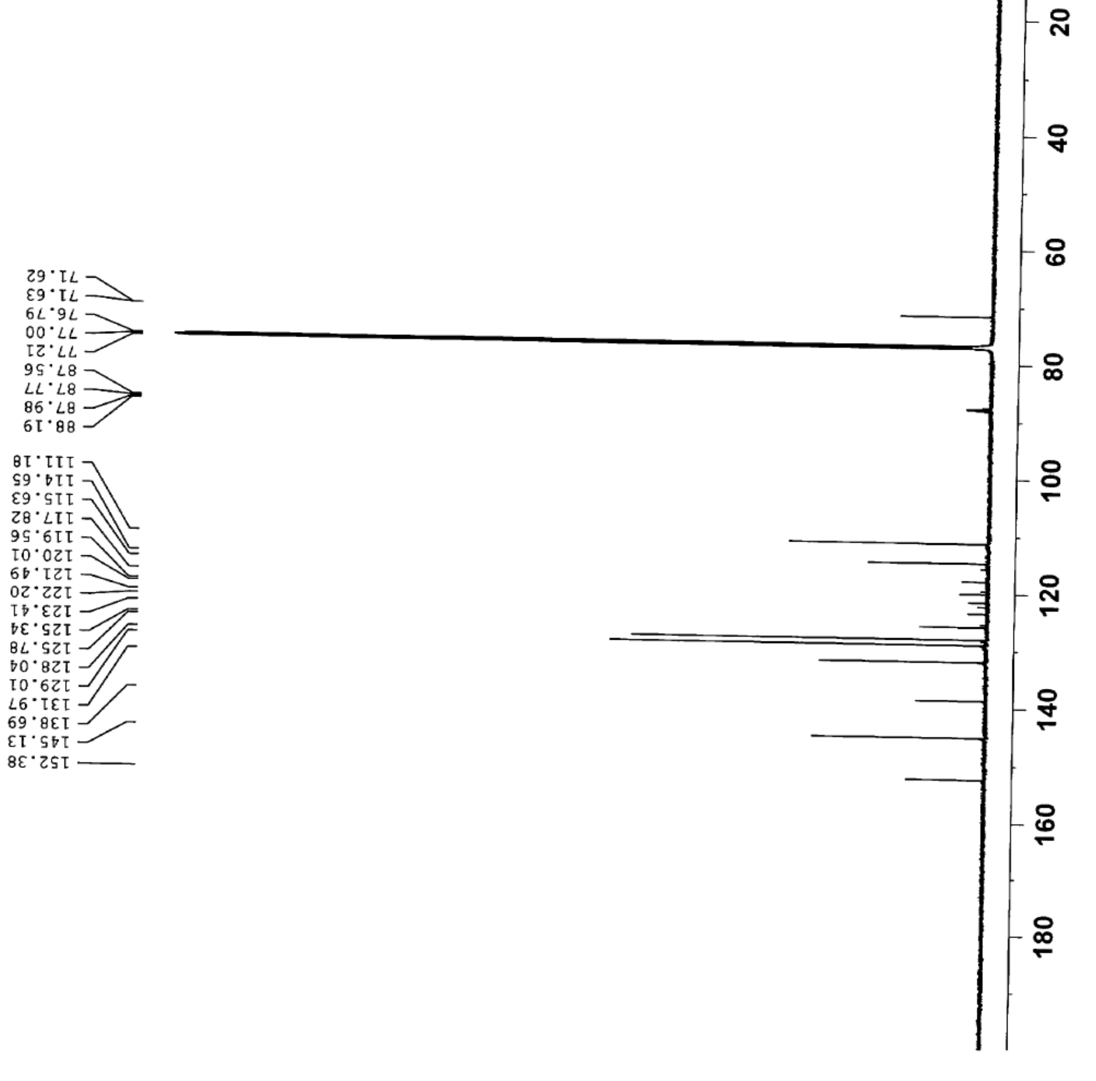

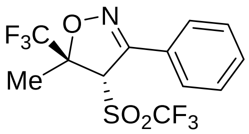

(4S*,5R*)-**1i**
(dr = 100:0)
^{1}H NMR

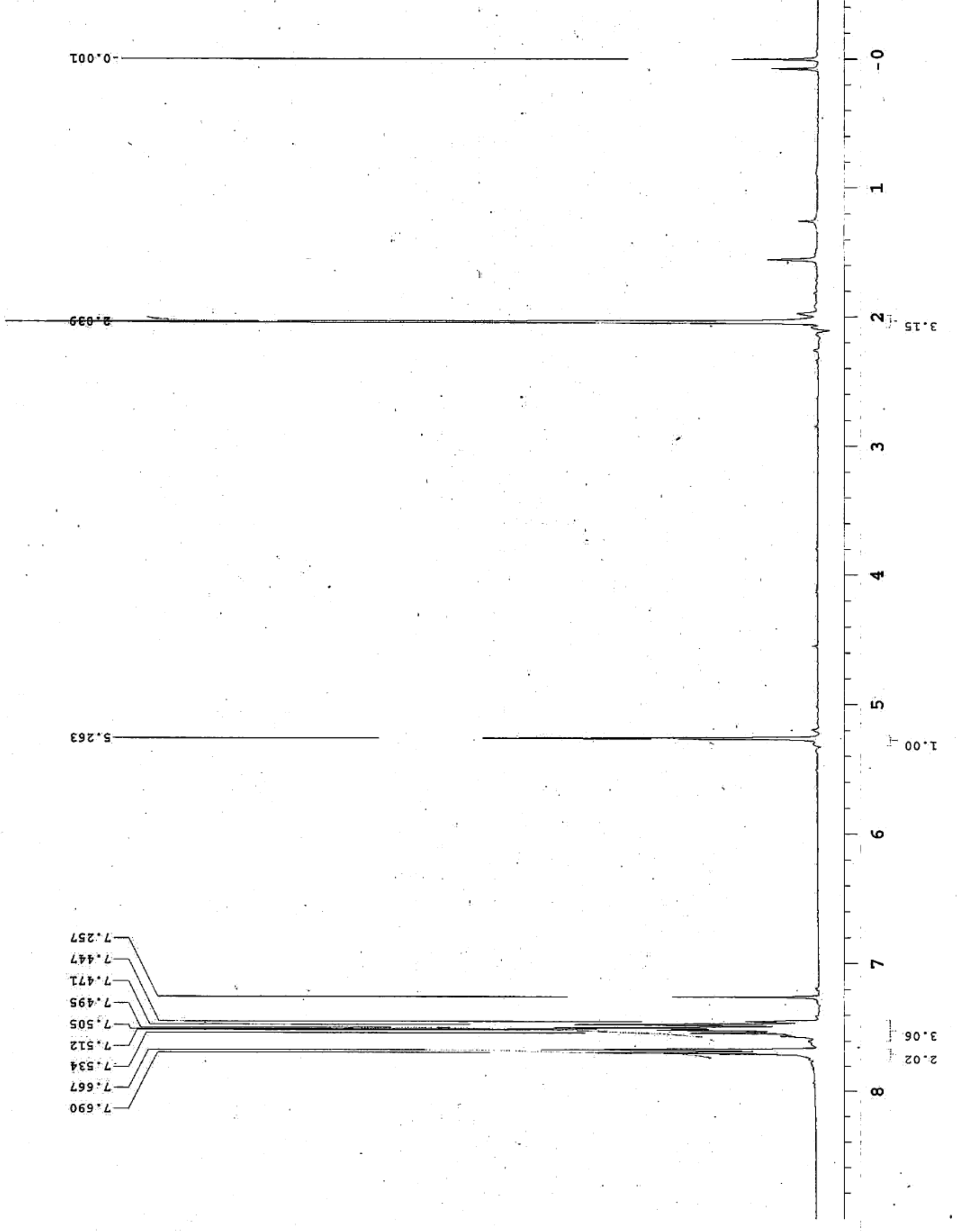

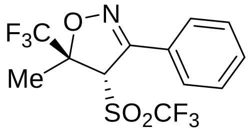

(4S*,5R*)-**1i**
(dr = 100:0)
^{19}F NMR

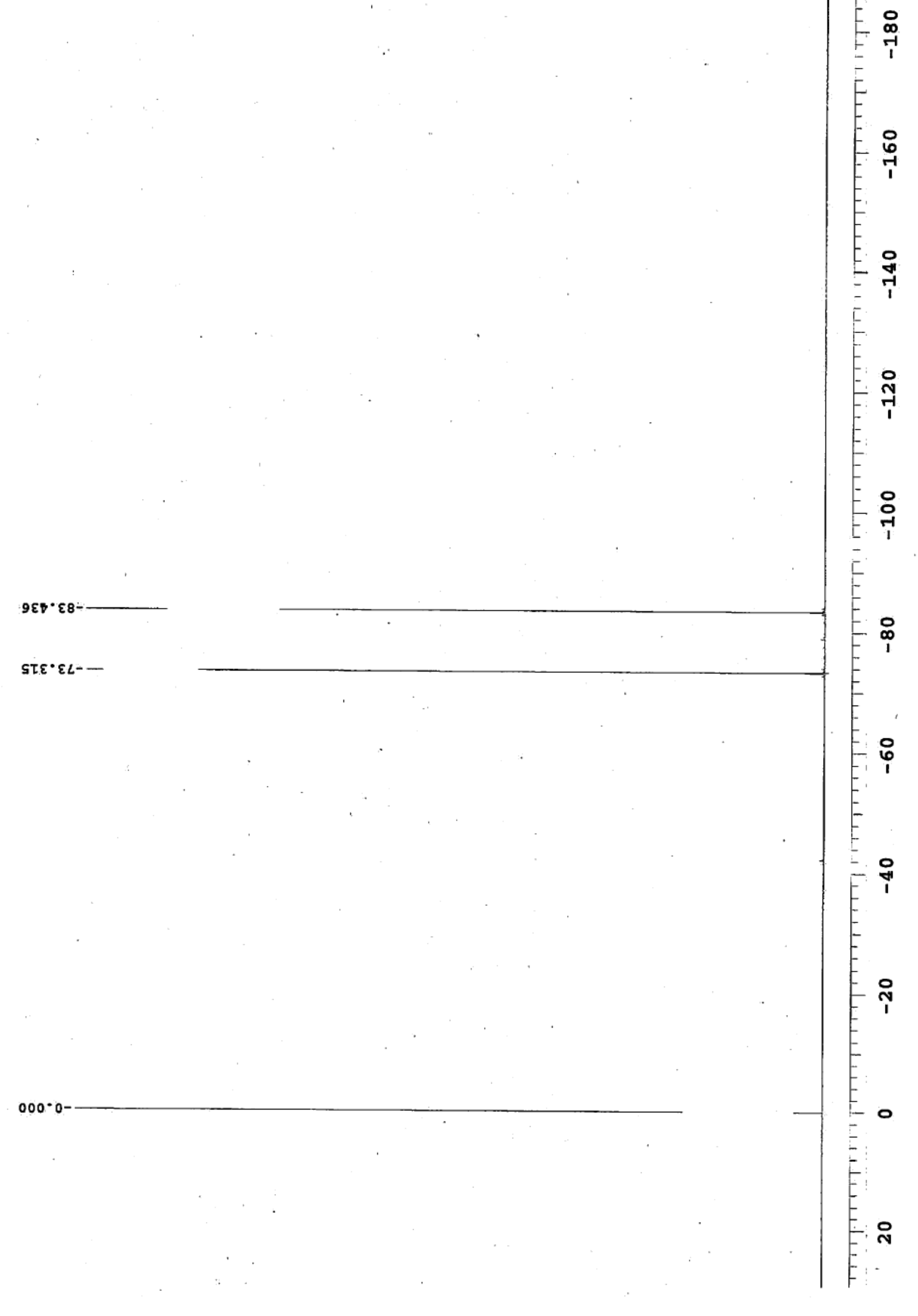

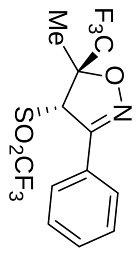

(4S*,5R*)-**1i**
(dr =100:0)
^{13}C NMR

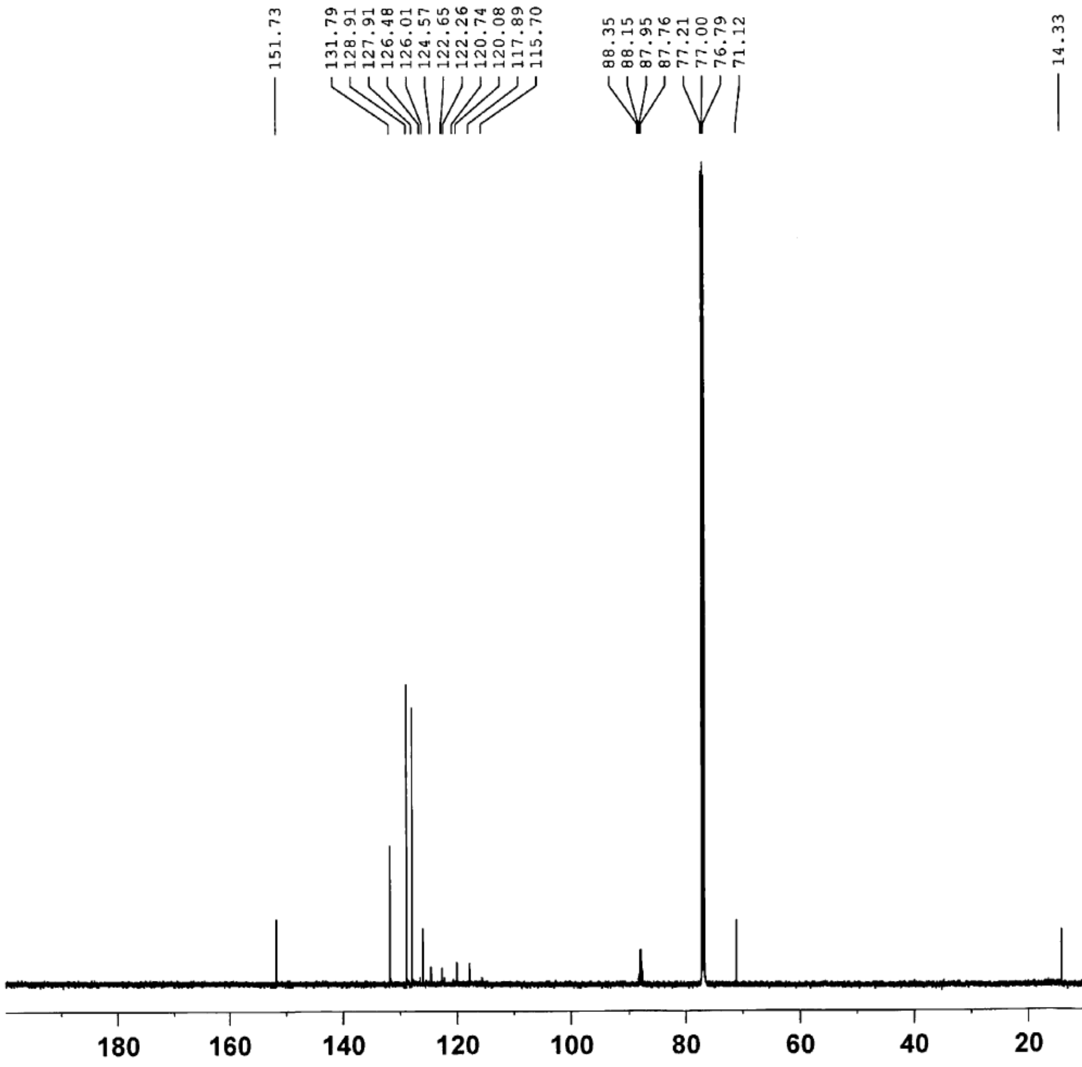


```
Current Data Parameters
NAME          KH-2864
EXPNO               1
PROCNO             10

F2 - Acquisition Parameters
Date_         20111216
Time             13.11
INSTRUM         drx600
PROBHD   5 mm BBO BB-1H
PULPROG        zgpg30
TD             131072
SOLVENT          CDCl3
NS               1587
DS                  4
SWH         45454.547 Hz
FIDRES       0.346791 Hz
AQ          1.4418530 sec
RG             3649.1
DW             11.000 usec
DE               6.00 usec
TE              294.6 K
D1         0.60000002 sec
d11        0.03000000 sec
DELTA      0.50000000 sec
TD0                 1

======== CHANNEL f1 ========
NUC1              13C
P1               8.20 usec
PL1              4.50 dB
SFO1      150.9223664 MHz

======== CHANNEL f2 ========
CPDPRG2       waltz16
NUC2               1H
PCPD2           82.00 usec
PL2             -4.00 dB
PL12            15.00 dB
PL13            15.00 dB
SFO2      600.1324005 MHz

F2 - Processing parameters
SI             131072
SF        150.9028121 MHz
WDW                EM
SSB                 0
LB               1.00 Hz
GB                  0
PC               1.40
```

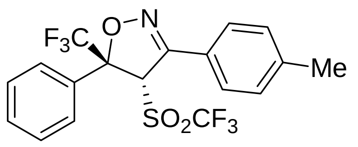

(4S*,5R*)-**1j**
(dr = 93:7)
^{1}H NMR

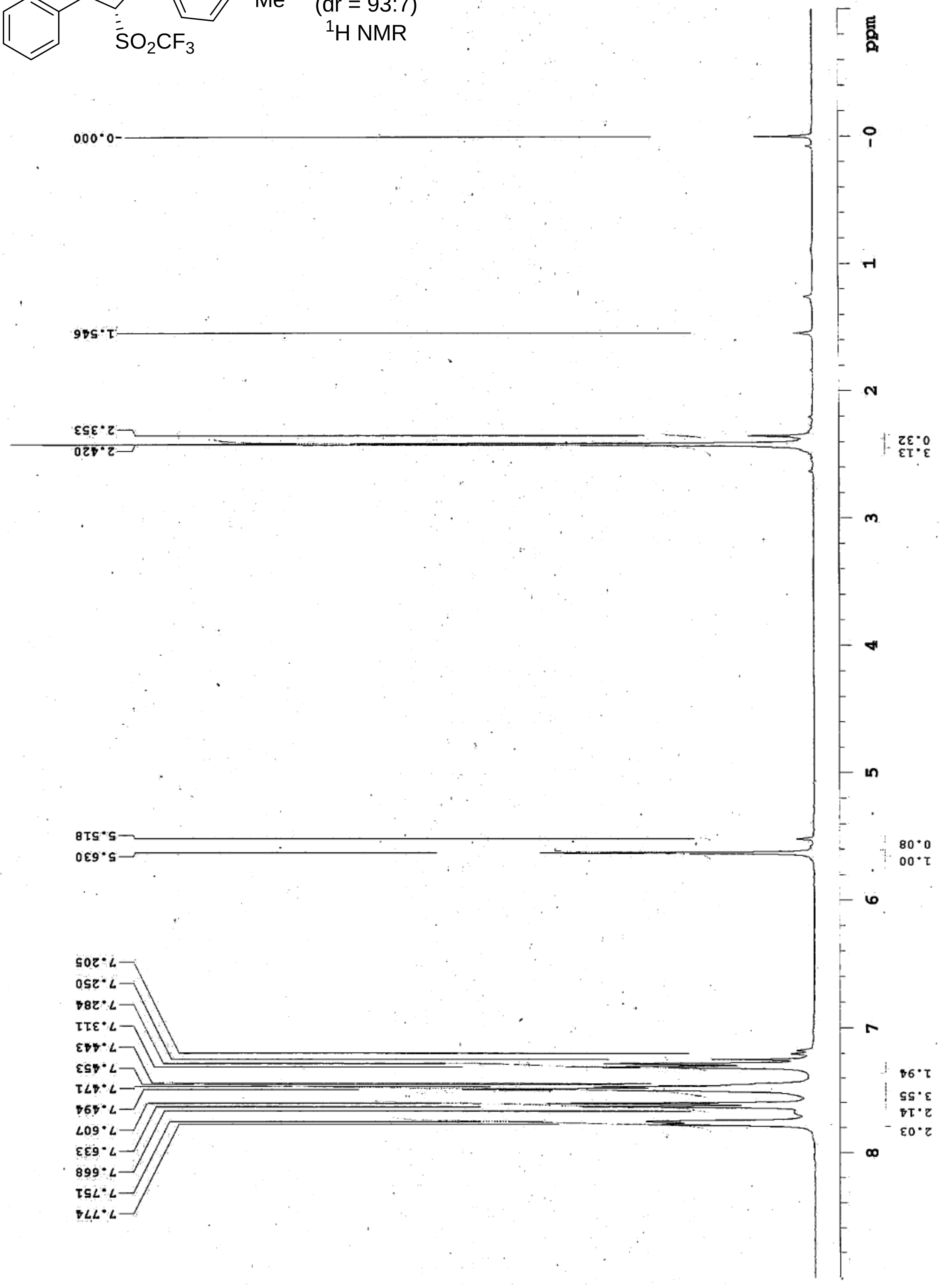

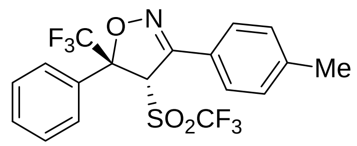

(4S*,5R*)-**1j**
(dr = 93:7)
^{19}F NMR

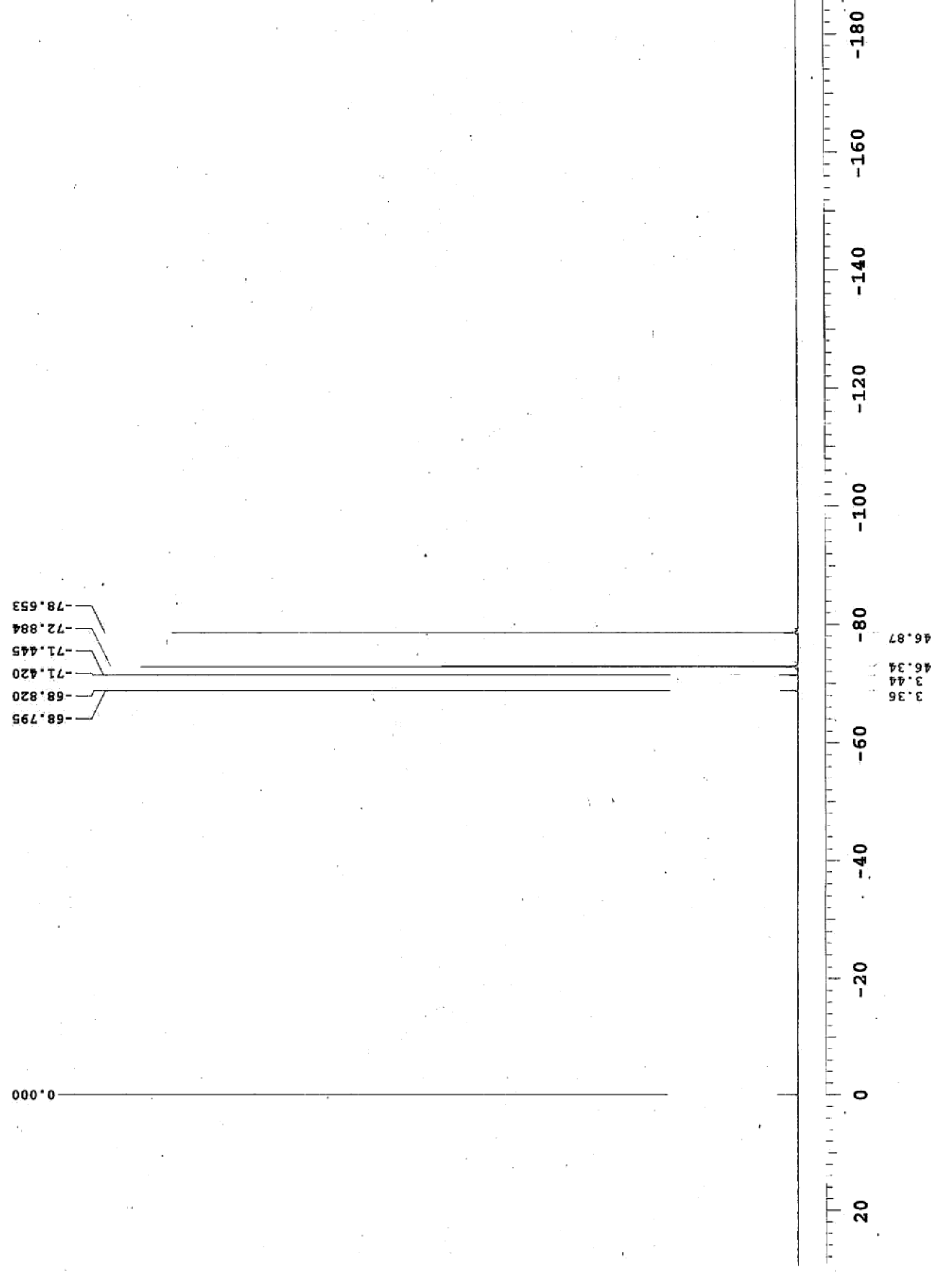

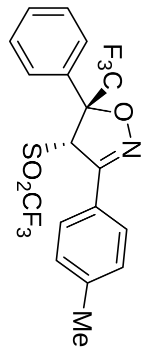

(4S*,5R*)-**1j**
(dr = 93:7)
^{13}C NMR

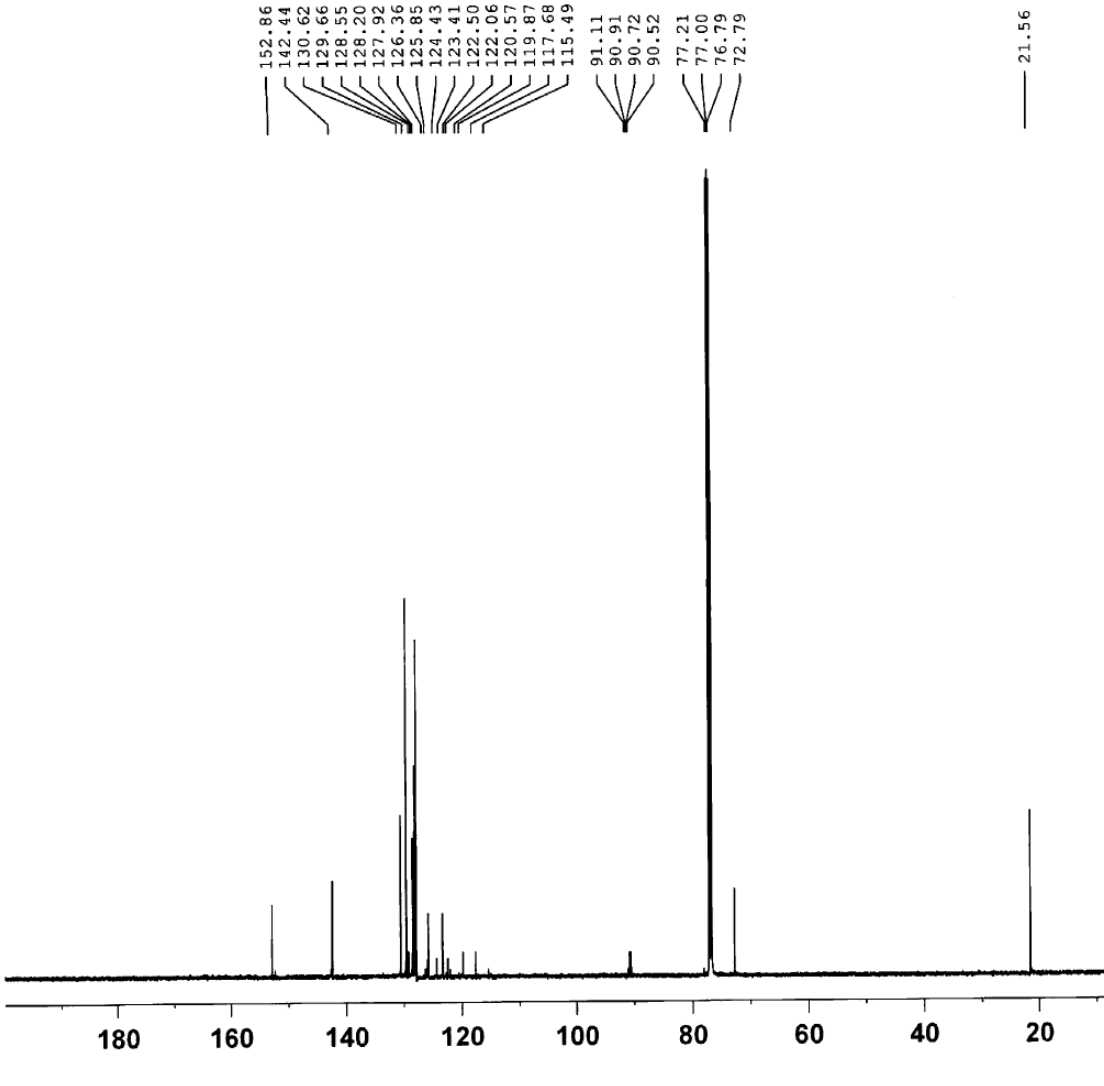


```
Current Data Parameters
NAME              SUY-290
EXPNO                   1
PROCNO                 10

F2 - Acquisition Parameters
Date_            20111216
Time                15.12
INSTRUM            drx600
PROBHD   5 mm BBO BB-1H
PULPROG           zgpg30
TD                 131072
SOLVENT             CDCl3
NS                   2025
DS                      4
SWH             45454.547 Hz
FIDRES           0.346791 Hz
AQ              1.4418530 sec
RG                  16384
DW                 11.000 usec
DE                   6.00 usec
TE                  296.2 K
D1             0.60000002 sec
d11            0.03000000 sec
DELTA          0.50000000 sec
TD0                     1

======== CHANNEL f1 ========
NUC1                  13C
P1                  10.00 usec
PL1                 -5.00 dB
SFO1          150.9223664 MHz

======== CHANNEL f2 ========
CPDPRG2           waltz16
NUC2                   1H
PCPD2               80.00 usec
PL2                 -4.00 dB
PL12                10.54 dB
PL13                10.54 dB
SFO2          600.1324005 MHz

F2 - Processing parameters
SI                 131072
SF            150.9028118 MHz
WDW                    EM
SSB                     0
LB                   1.00 Hz
GB                      0
PC                   1.40
```

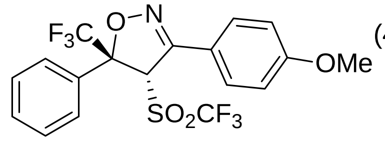

(4S*,5R*)-**1k**
(dr = 93:7)
^{1}H NMR

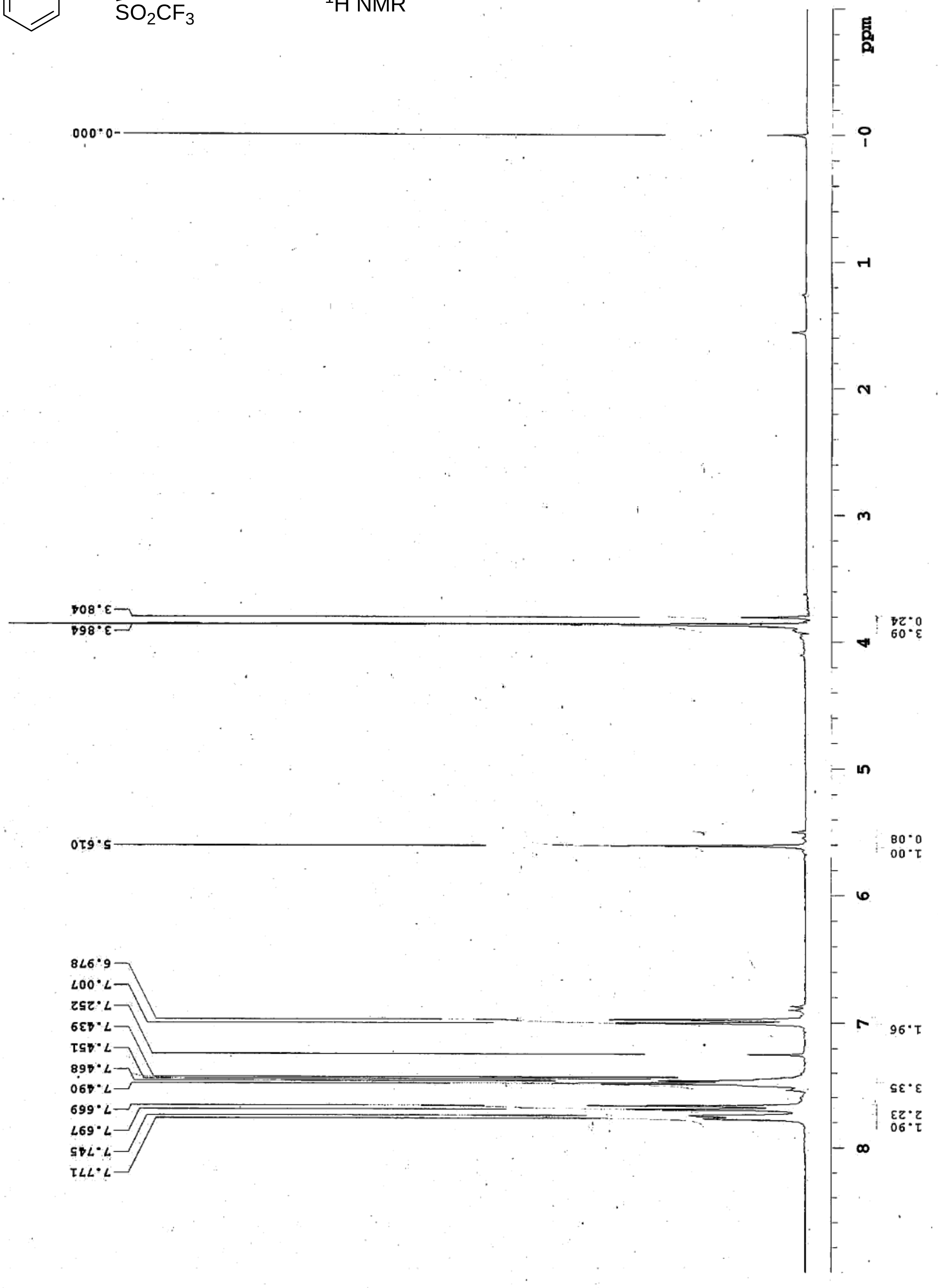

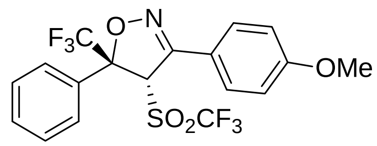

(4S*,5R*)-**1k**
(dr = 93:7)
^{19}F NMR

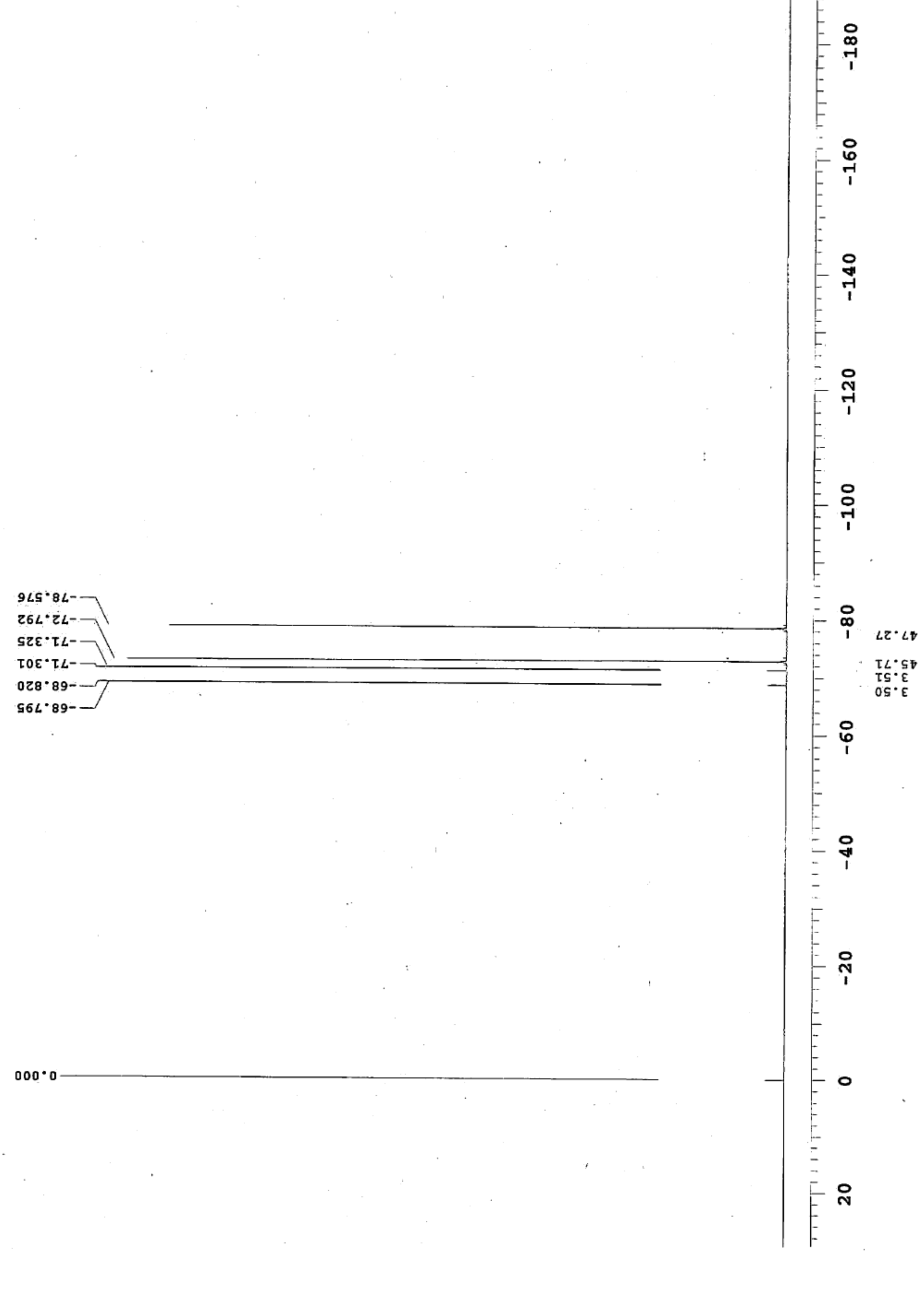

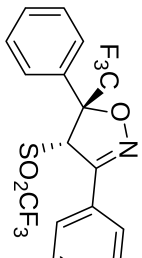

(4S*,5R*)-**1k** (dr = 93:7) ^{13}C NMR

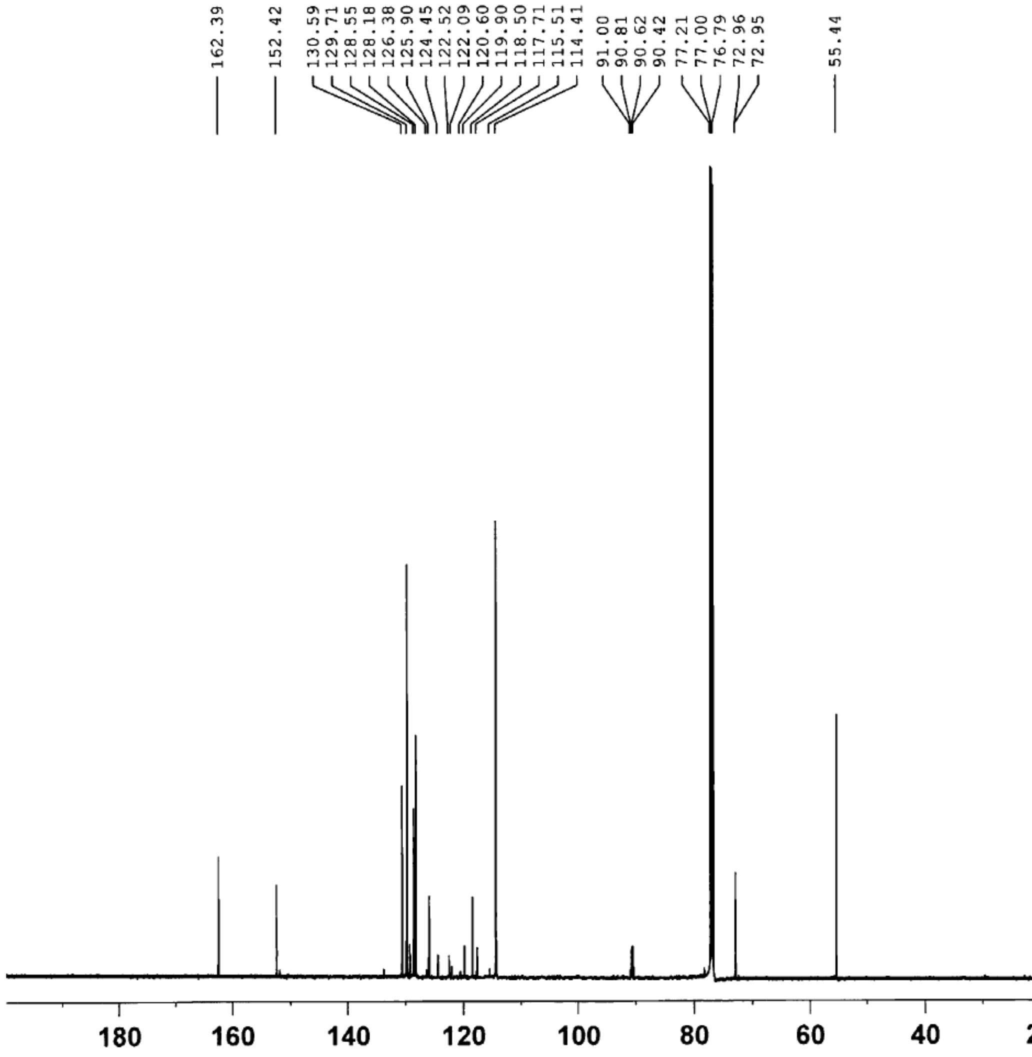


```
Current Data Parameters
NAME              SUY-291
EXPNO                   1
PROCNO                 10

F2 - Acquisition Parameters
Date_            20111216
Time                16.30
INSTRUM            drx600
PROBHD   5 mm BBO BB-1H
PULPROG           zgpg30
TD                 131072
SOLVENT             CDCl3
NS                   4550
DS                      4
SWH           45454.547 Hz
FIDRES         0.346791 Hz
AQ            1.4418530 sec
RG                   8192
DW               11.000 usec
DE                 6.00 usec
TE                296.4 K
D1           0.60000002 sec
d11          0.03000000 sec
DELTA        0.50000000 sec
TD0                     1

======== CHANNEL f1 ========
NUC1                  13C
P1                  10.00 usec
PL1                 -5.00 dB
SFO1          150.9223664 MHz

======== CHANNEL f2 ========
CPDPRG2           waltz16
NUC2                   1H
PCPD2               80.00 usec
PL2                 -4.00 dB
PL12                10.54 dB
PL13                10.54 dB
SFO2          600.1324005 MHz

F2 - Processing parameters
SI                 131072
SF            150.9028111 MHz
WDW                    EM
SSB                     0
LB                   1.00 Hz
GB                      0
PC                   1.40
```

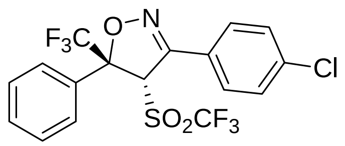

(4S*,5R*)-**1l**
(dr = 94:6)
^{1}H NMR

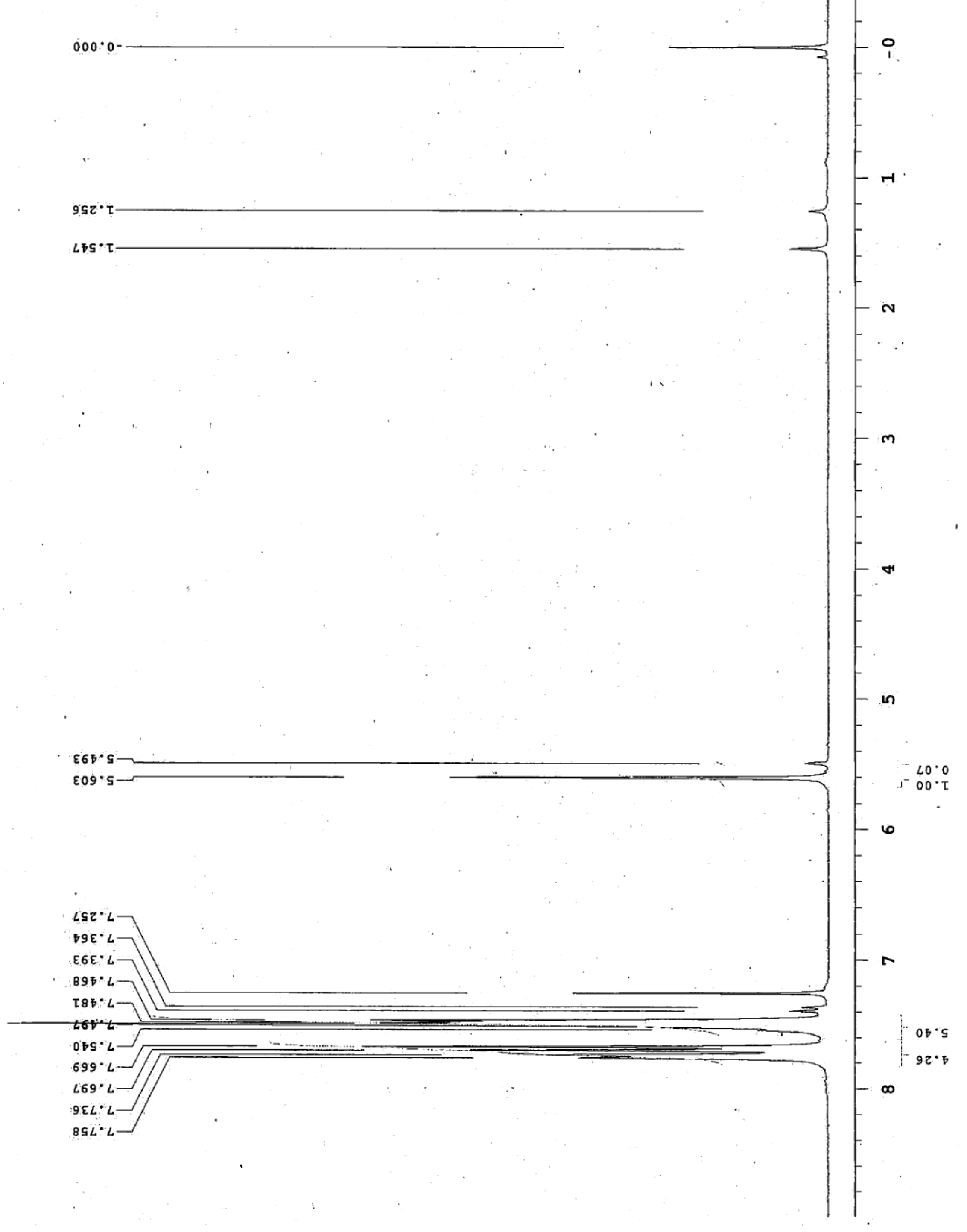

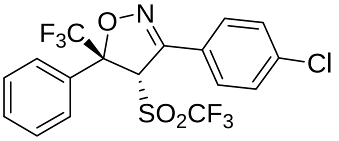

(4S*,5R*)-**1l**
(dr = 94:6)
^{19}F NMR

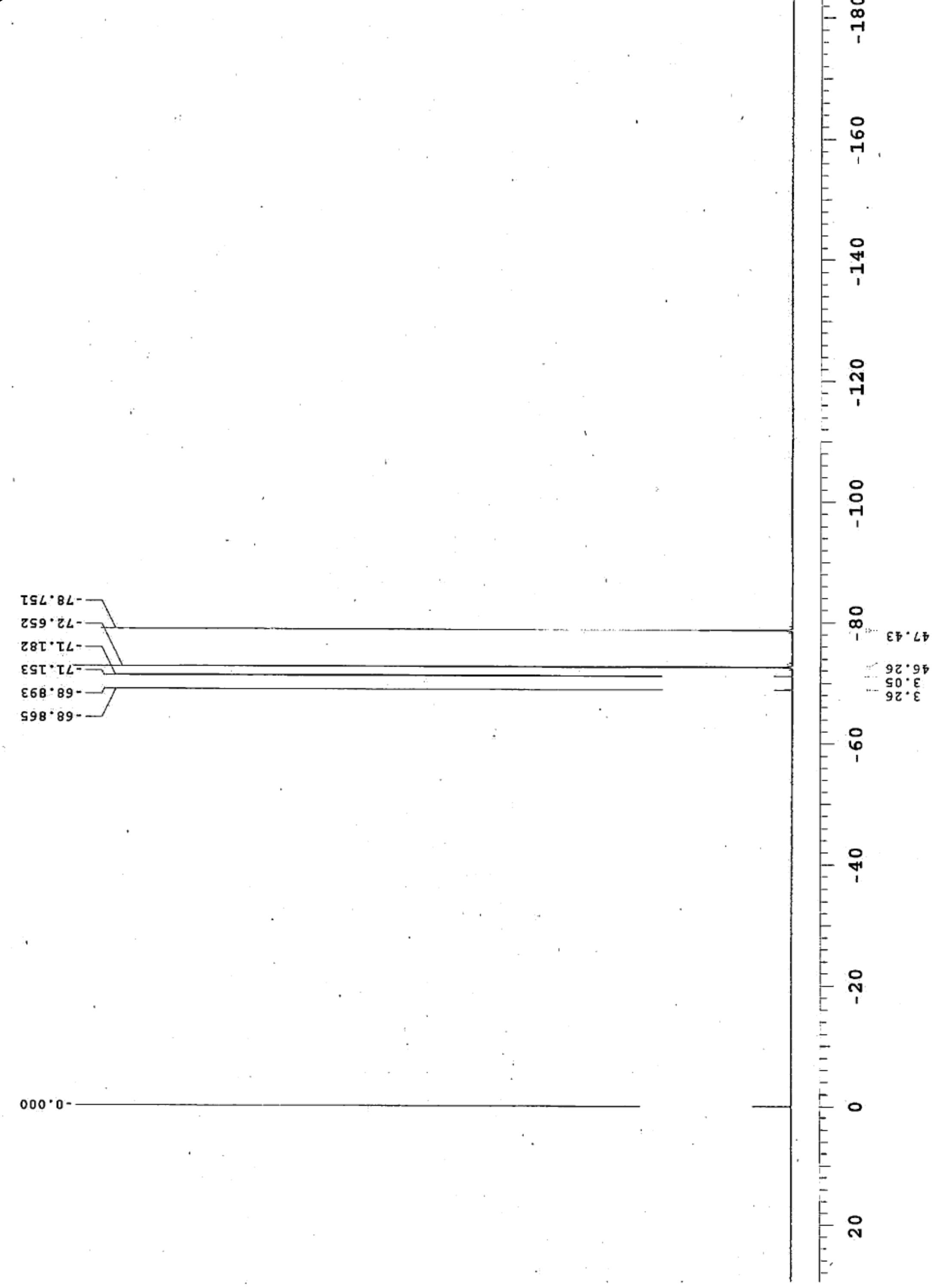

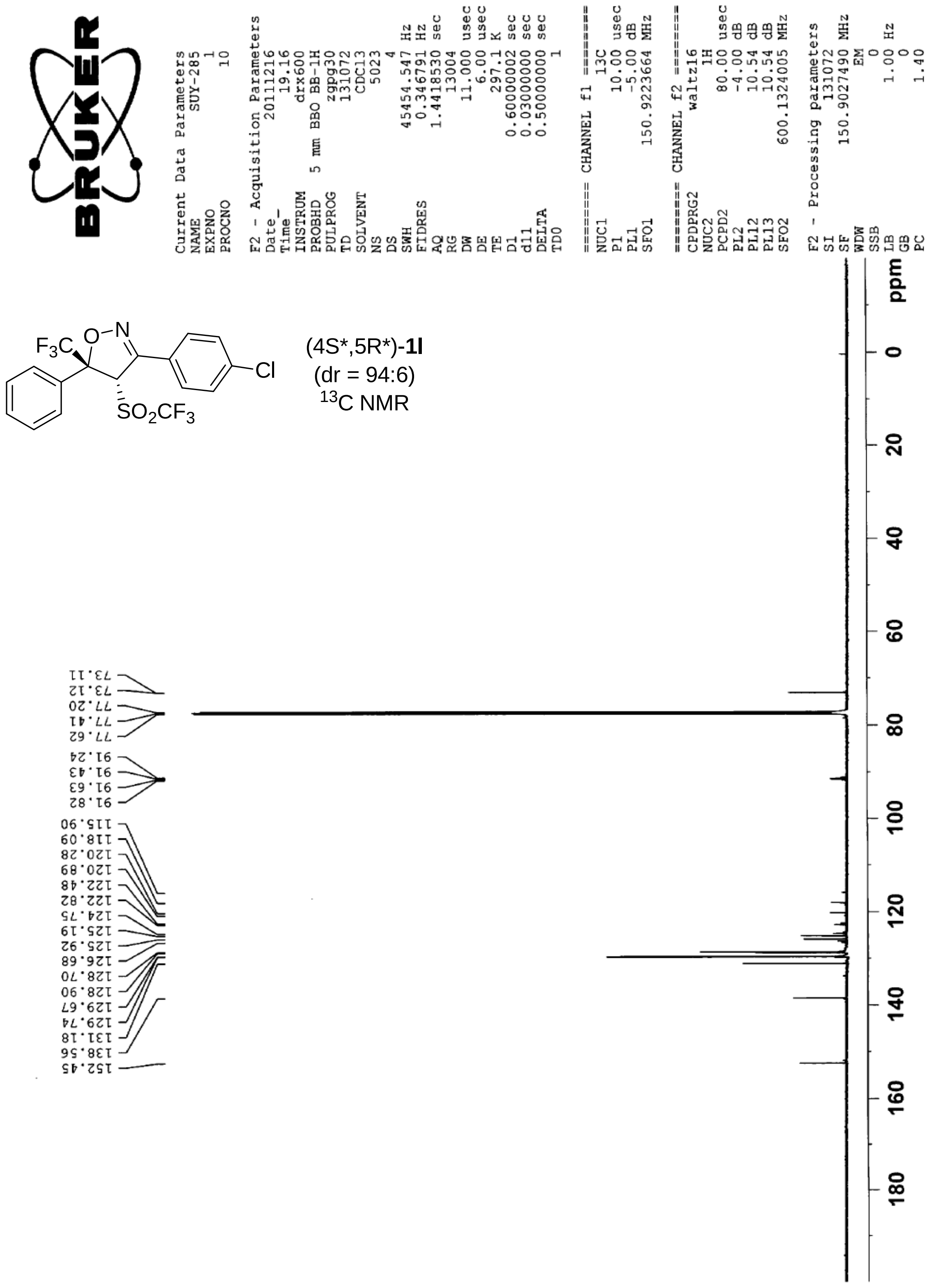

(4S*,5R*)-**1l**
(dr = 94:6)
^{13}C NMR

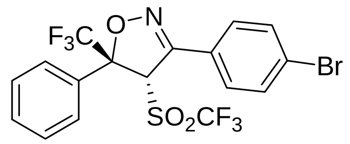

(4S*,5R*)-**1m**
(dr = 94:6)
^{1}H NMR

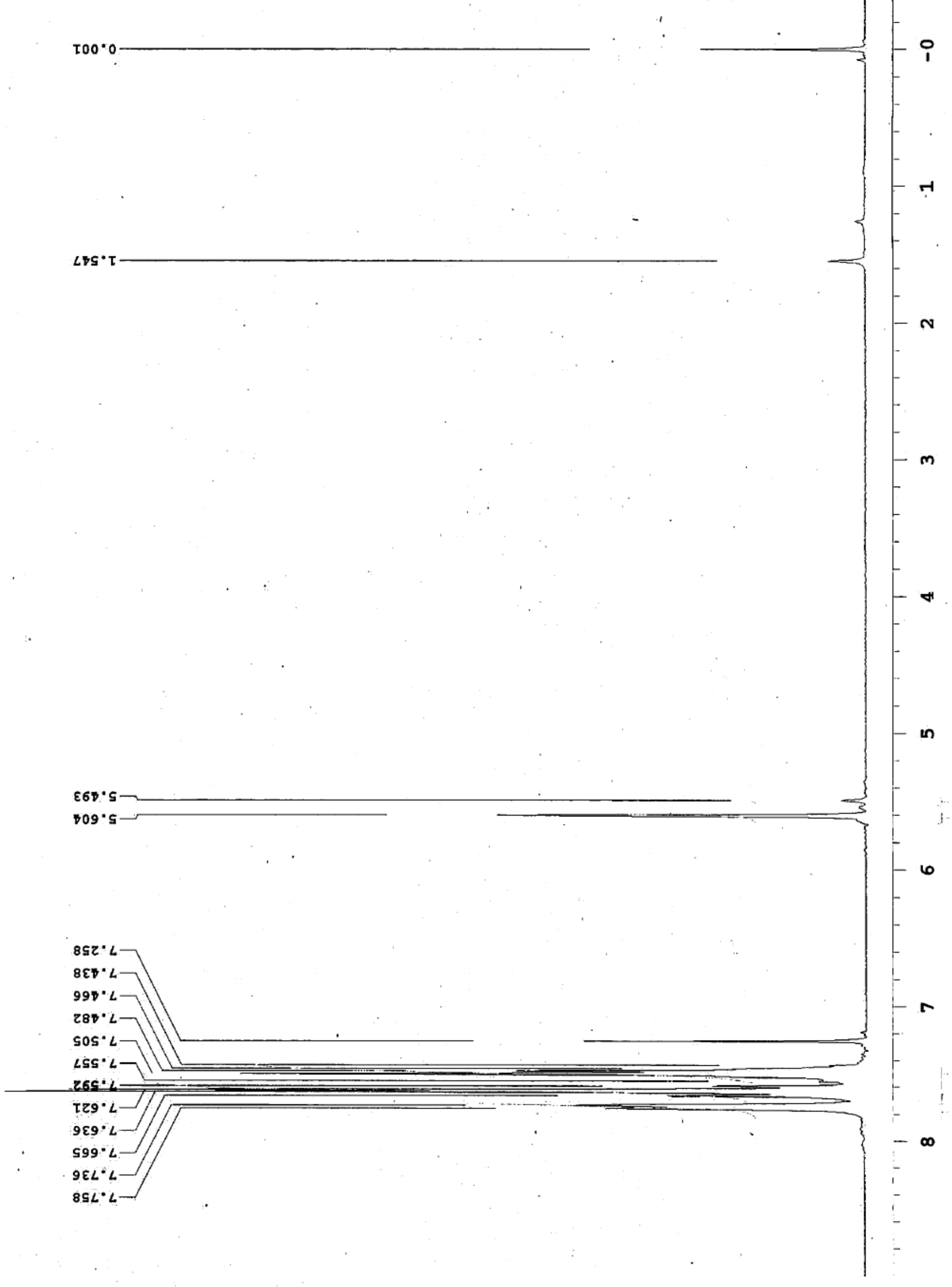

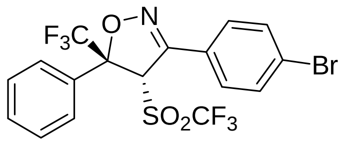

(4S*,5R*)-**1m**
(dr = 94:6)
^{19}F NMR

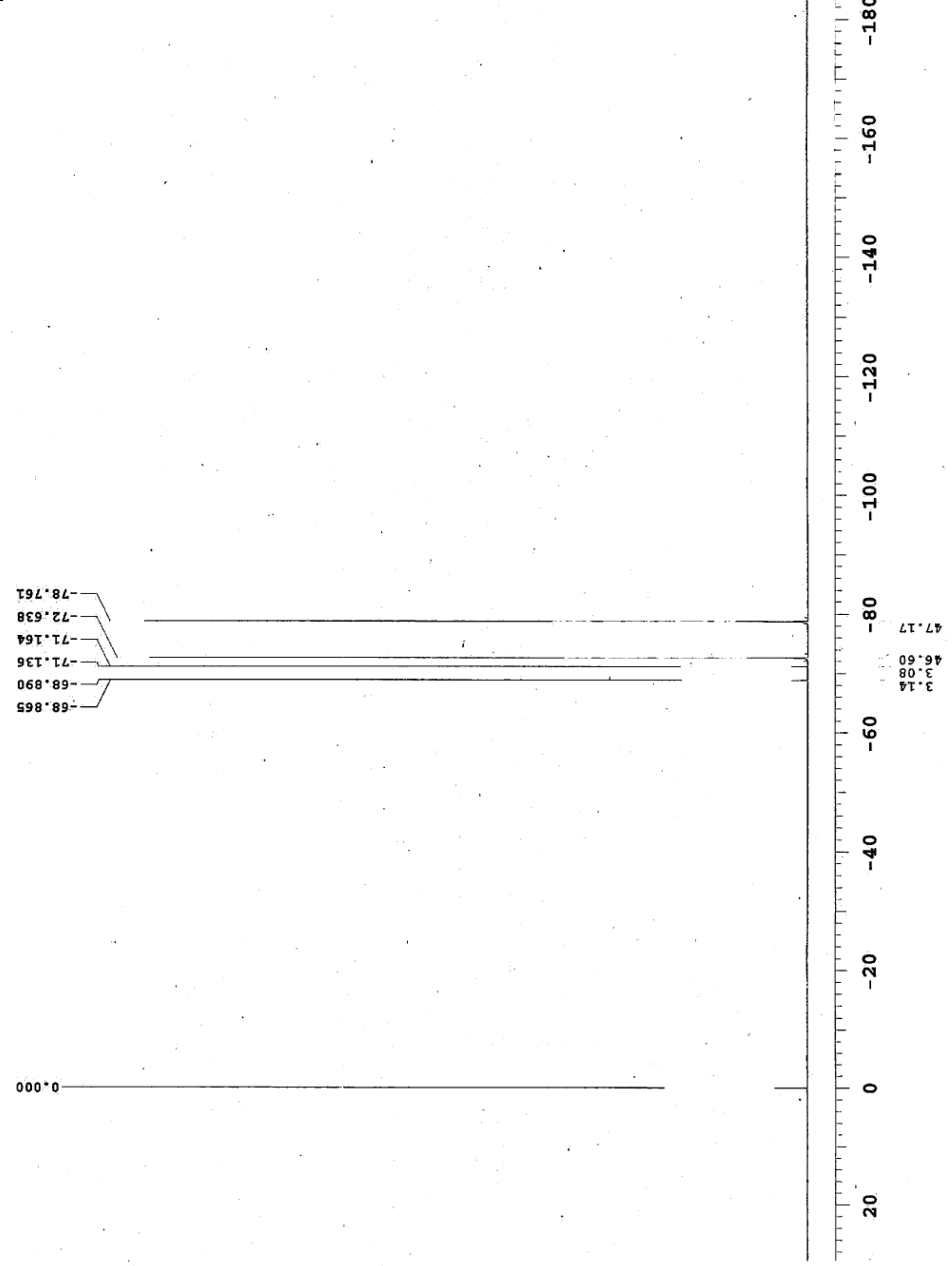

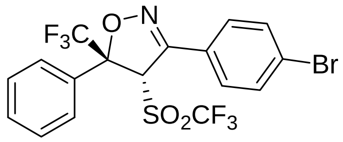

(4S*,5R*)-**1m**
(dr = 94:6)
^{13}C NMR

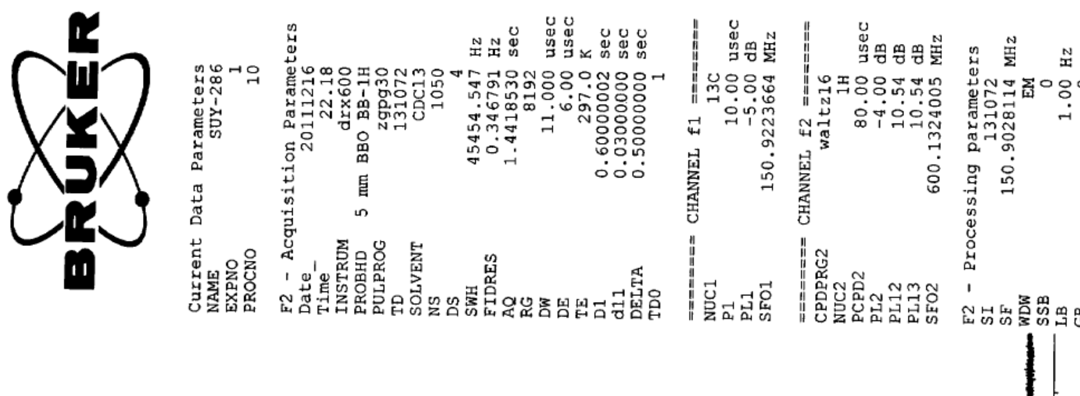

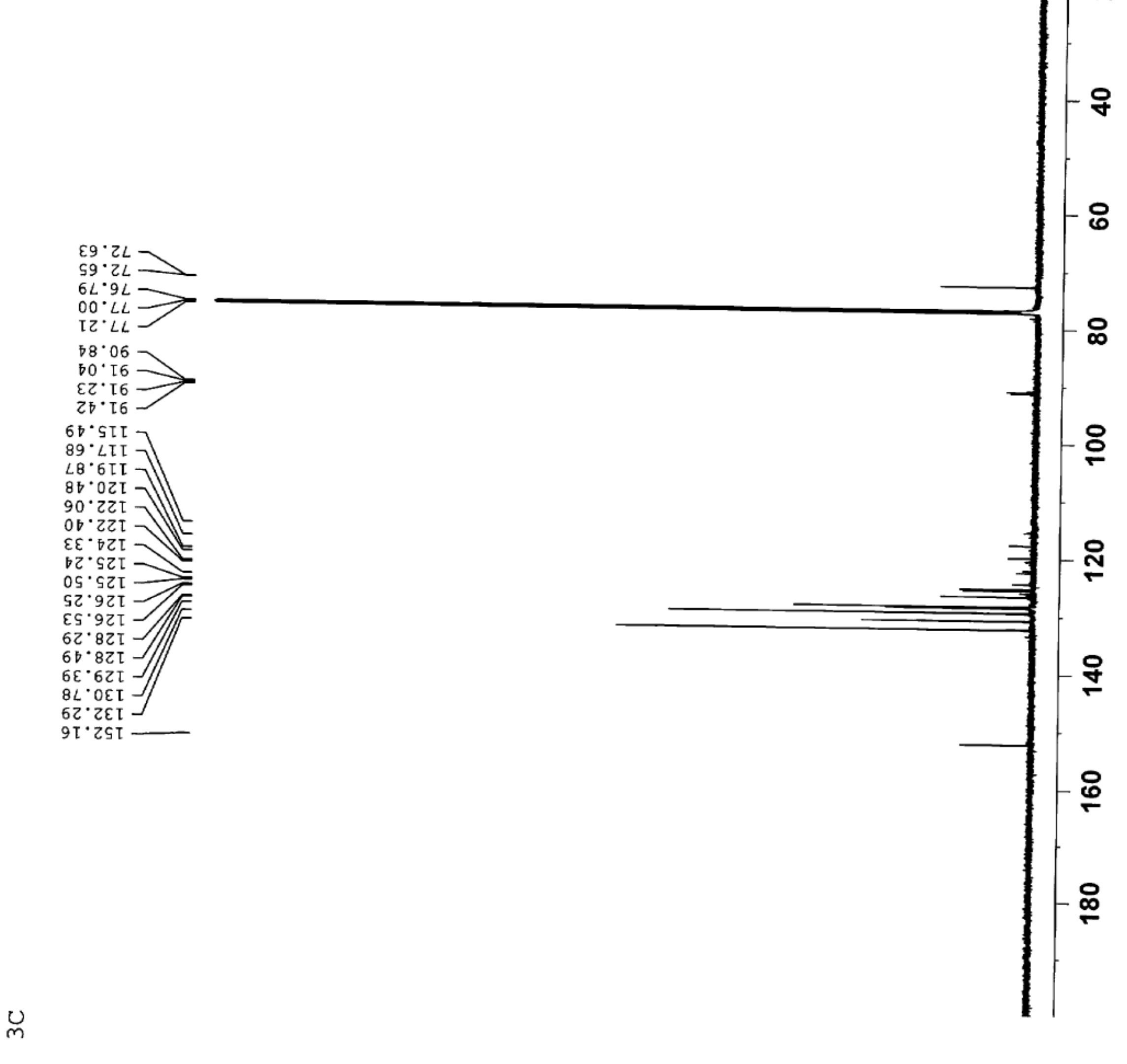

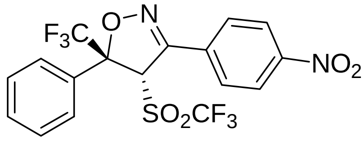

(4S*,5R*)-**1n**
(dr = 95:5)
^{1}H NMR

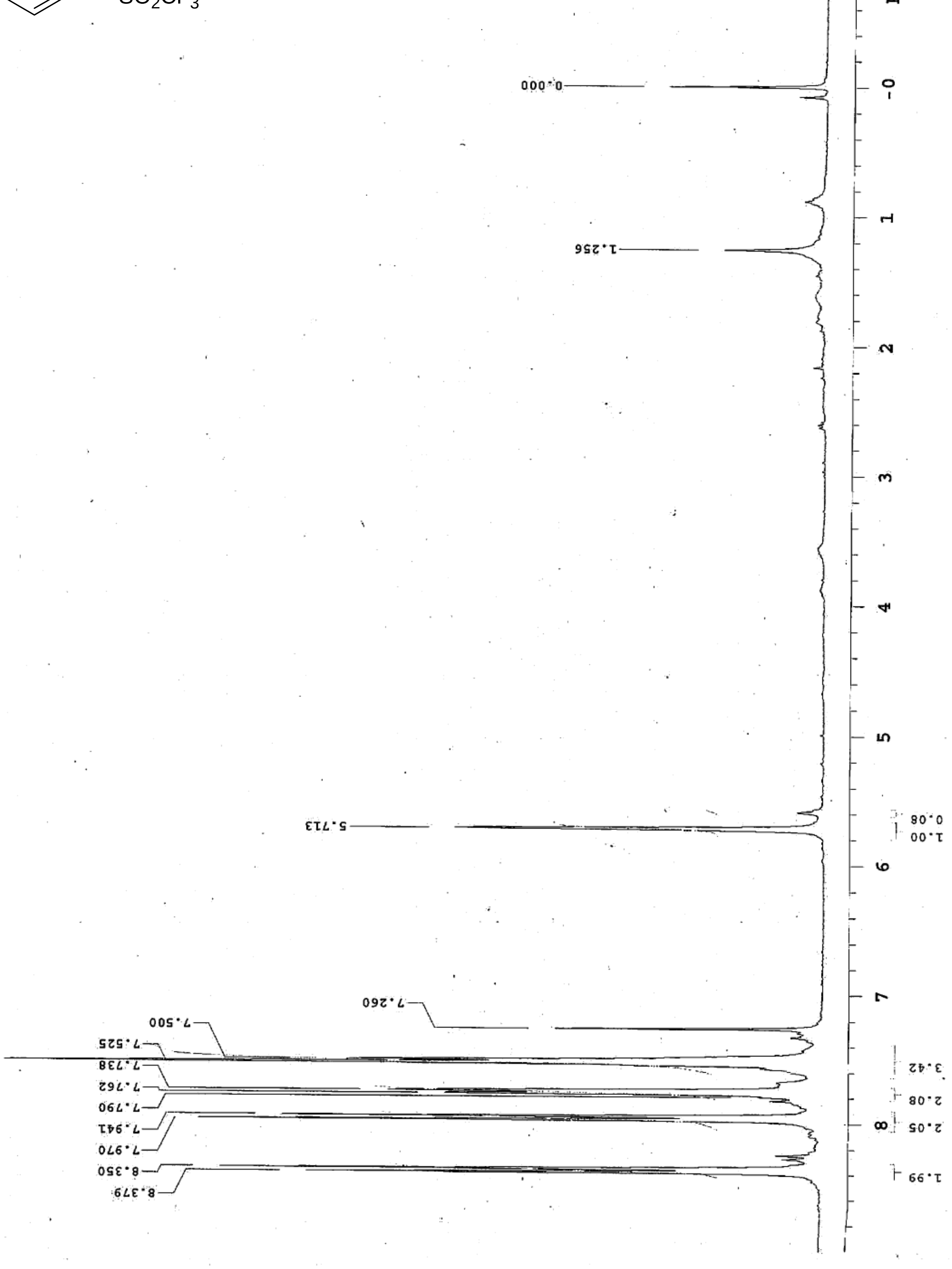

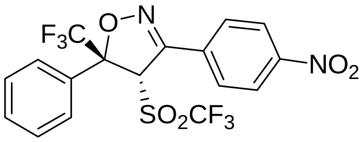

(4S*,5R*)-**1n**
(dr = 95:5)
^{19}F NMR

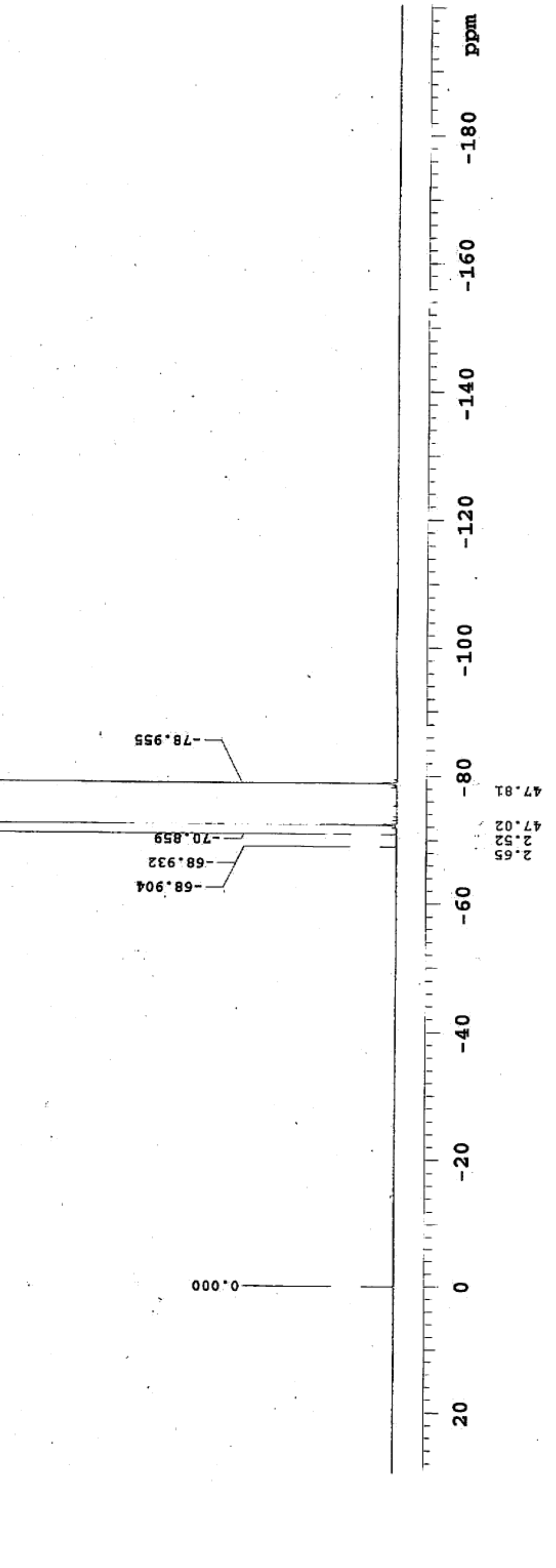

C

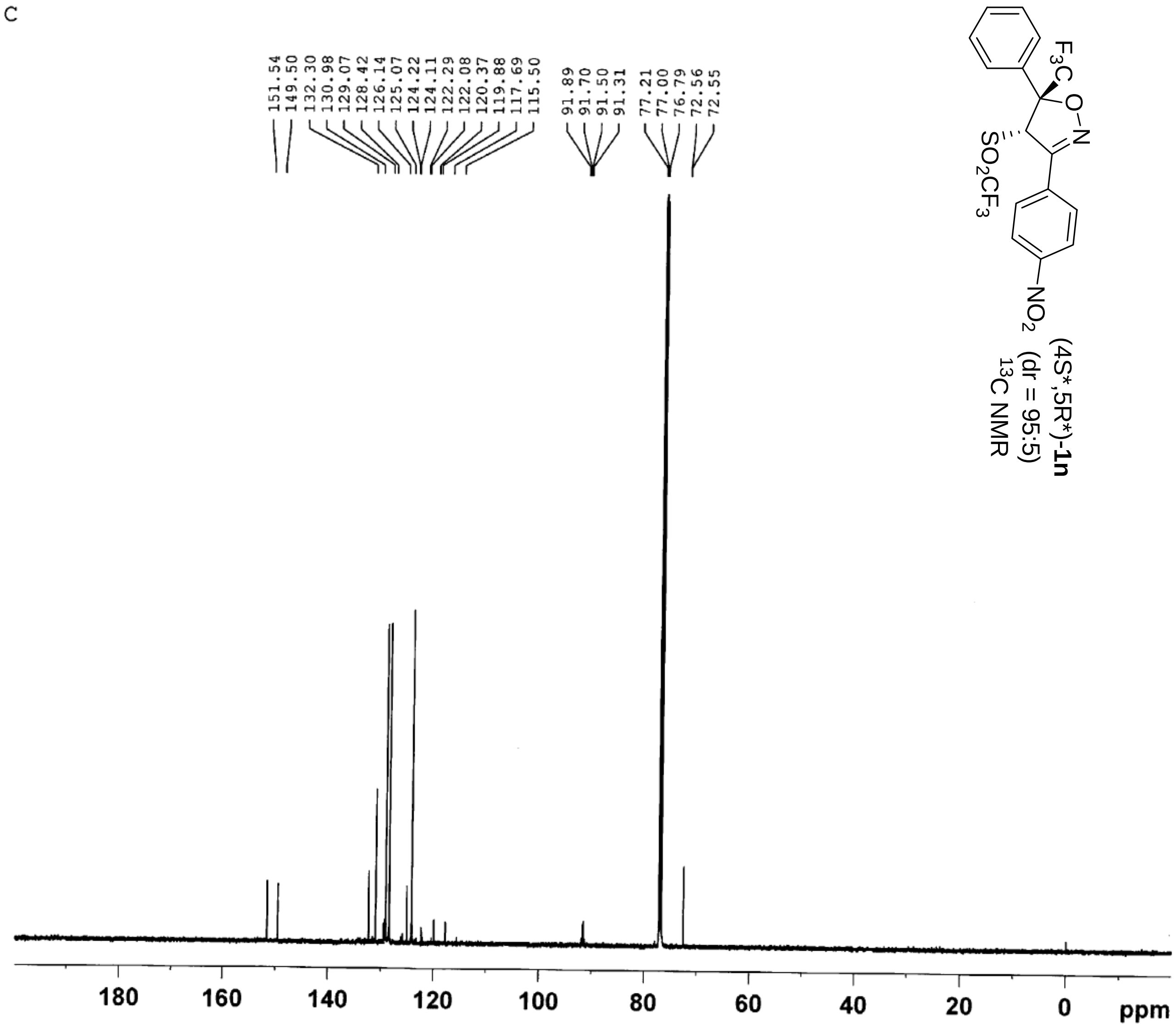

(4S*,5R*)-**1n**
(dr = 95:5)
^{13}C NMR

BRUKER

```
Current Data Parameters
NAME              SUY-292
EXPNO                   1
PROCNO                 10

F2 - Acquisition Parameters
Date_            20111216
Time                23.01
INSTRUM            drx600
PROBHD   5 mm BBO BB-1H
PULPROG           zgpg30
TD                 131072
SOLVENT             CDCl3
NS                   2033
DS                      4
SWH             45454.547 Hz
FIDRES           0.346791 Hz
AQ              1.4418530 sec
RG                   8192
DW                 11.000 usec
DE                   6.00 usec
TE                  296.8 K
D1             0.60000002 sec
d11            0.03000000 sec
DELTA          0.50000000 sec
TD0                     1

======== CHANNEL f1 ========
NUC1                  13C
P1                  10.00 usec
PL1                 -5.00 dB
SFO1          150.9223664 MHz

======== CHANNEL f2 ========
CPDPRG2           waltz16
NUC2                   1H
PCPD2               80.00 usec
PL2                 -4.00 dB
PL12                10.54 dB
PL13                10.54 dB
SFO2          600.1324005 MHz

F2 - Processing parameters
SI                 131072
SF            150.9028117 MHz
WDW                    EM
SSB                     0
LB                   1.00 Hz
GB                      0
PC                   1.40
```

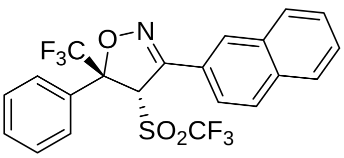

(4S*,5R*)-**1o**
(dr = 96:4)
^{1}H NMR

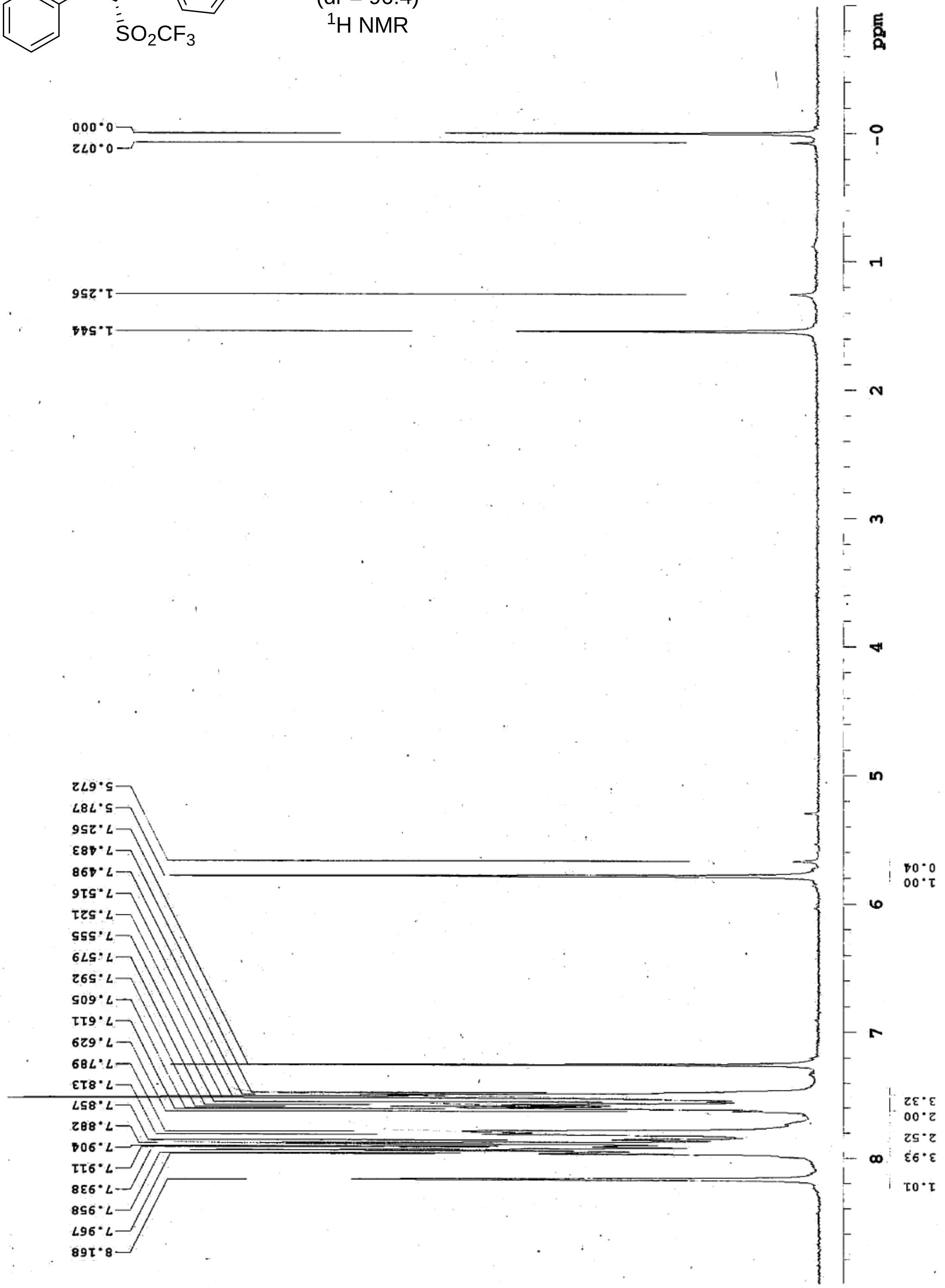

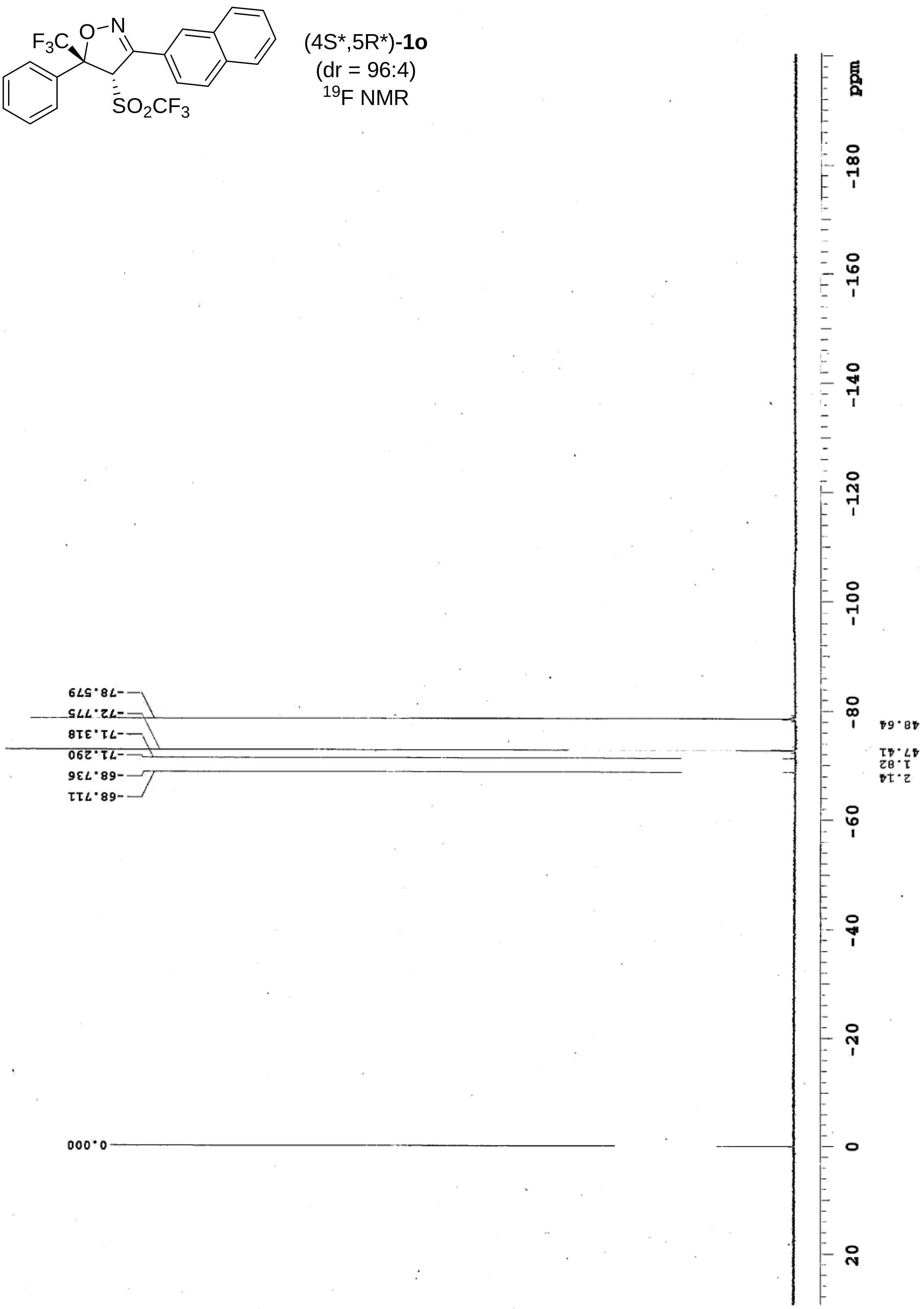

(4S*,5R*)-**1o**
(dr = 96:4)
^{19}F NMR

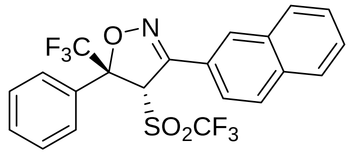

(4S*,5R*)-**1o**
(dr = 96:4)
^{13}C NMR

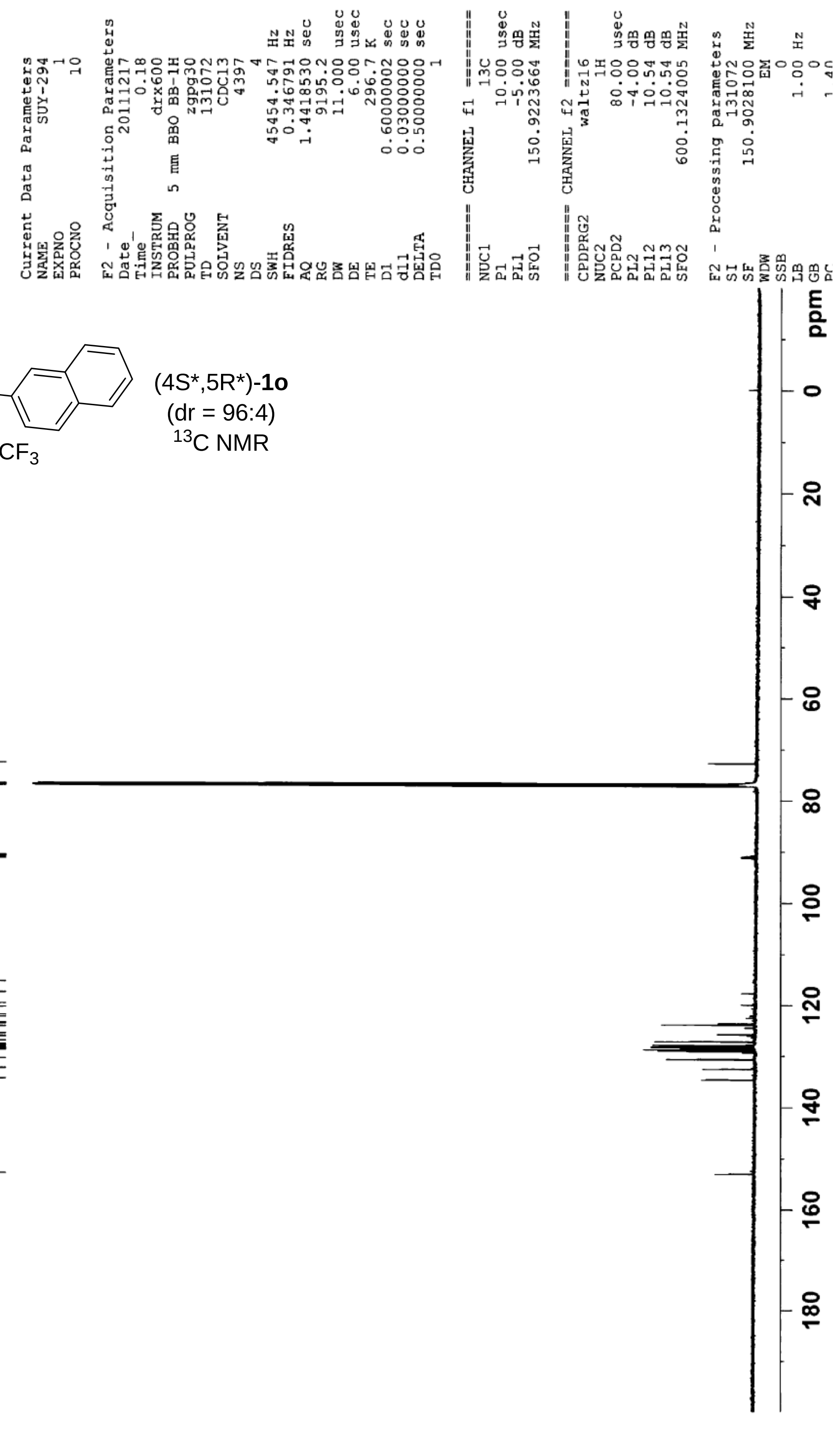

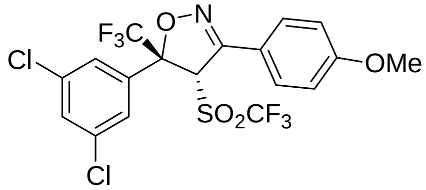

(4S*,5R*)-**1p**
(dr = 95:5)
^{1}H NMR

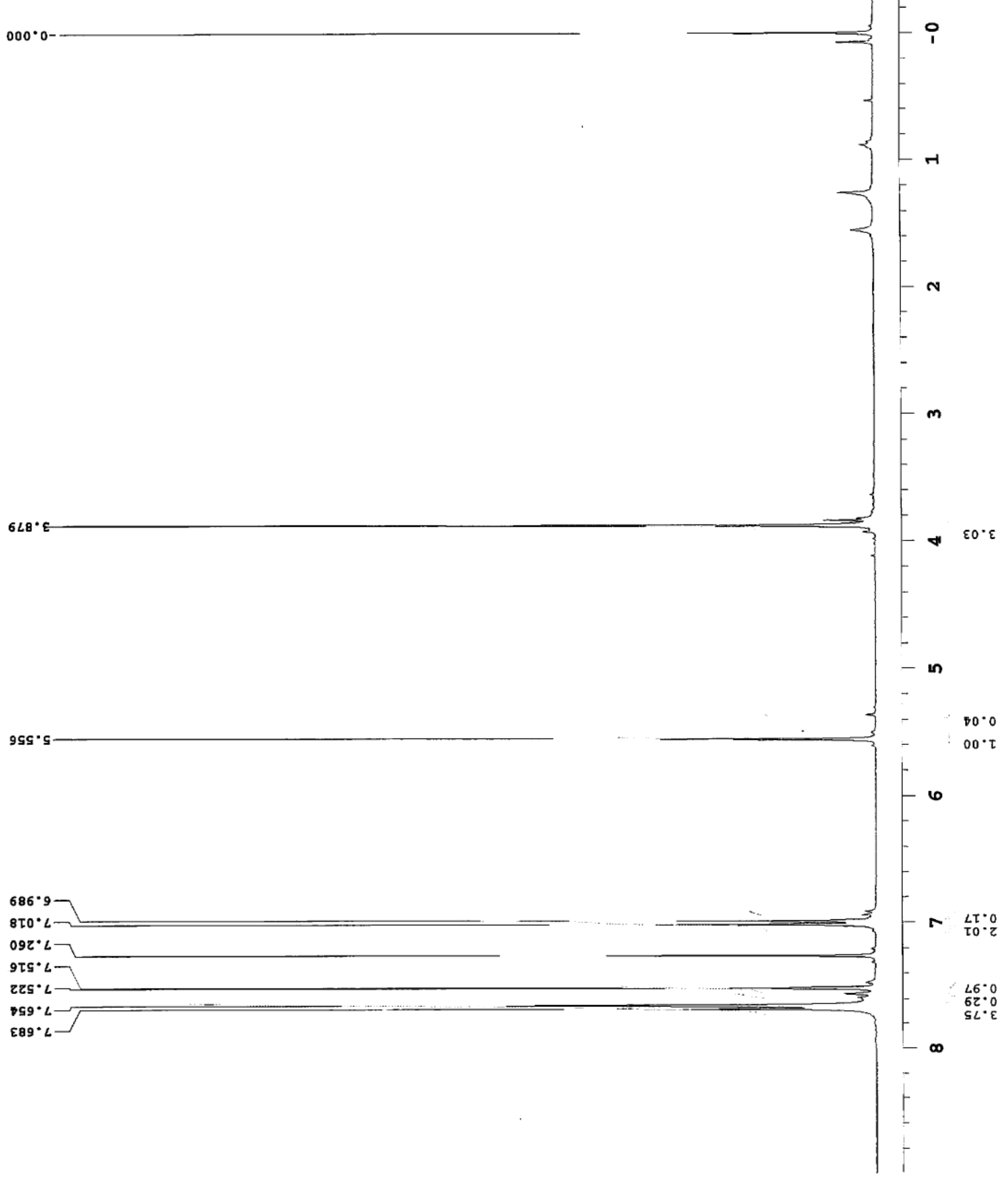

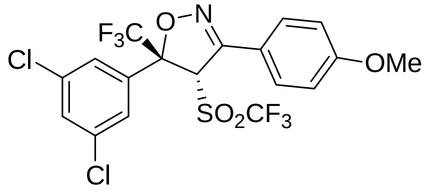

(4S*,5R*)-**1p**
(dr = 95:5)
^{19}F NMR

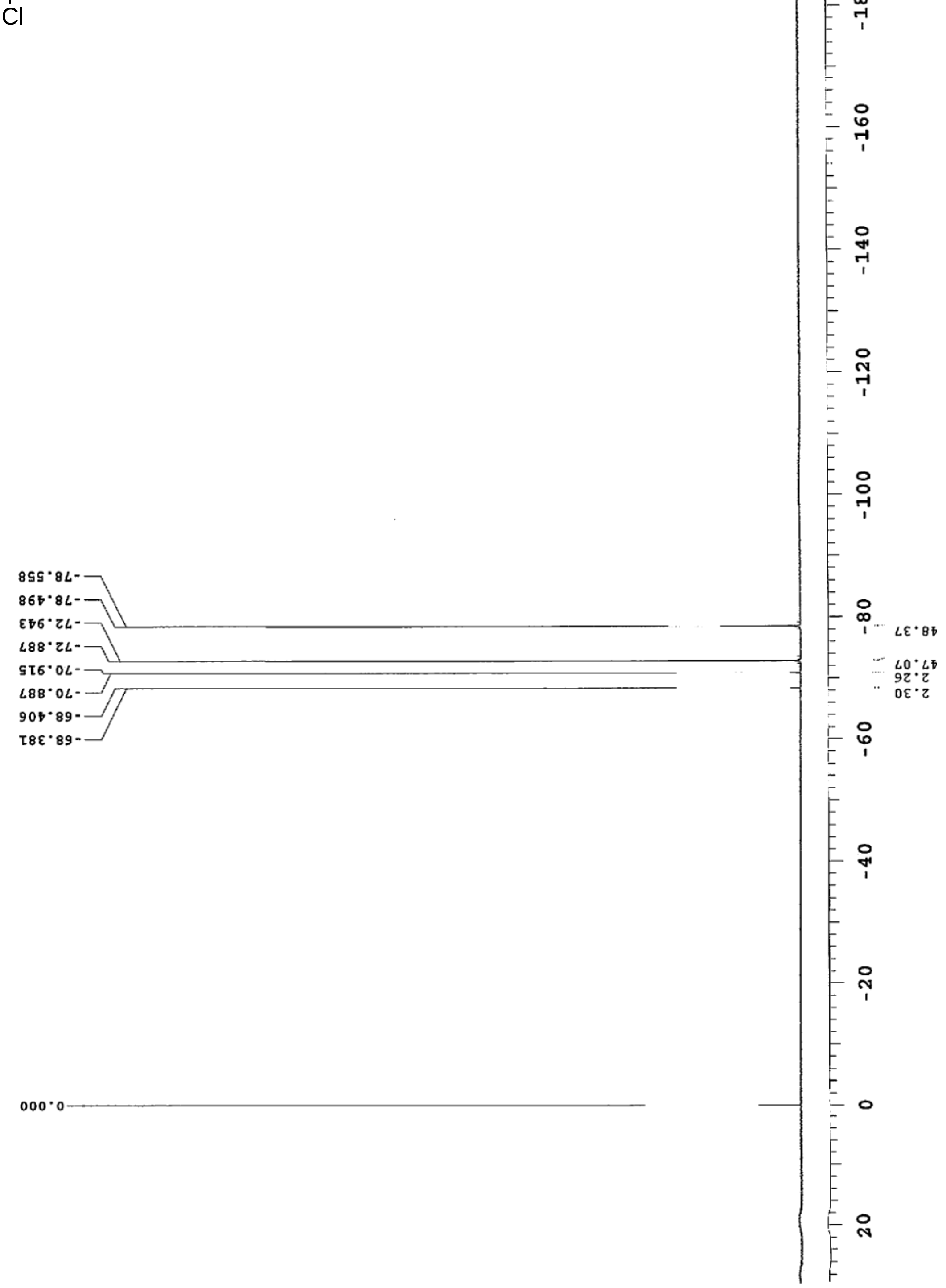

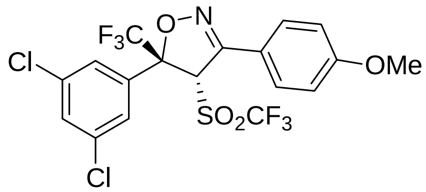

(4S*,5R*)-**1p**
(dr = 95:5)
^{13}C NMR

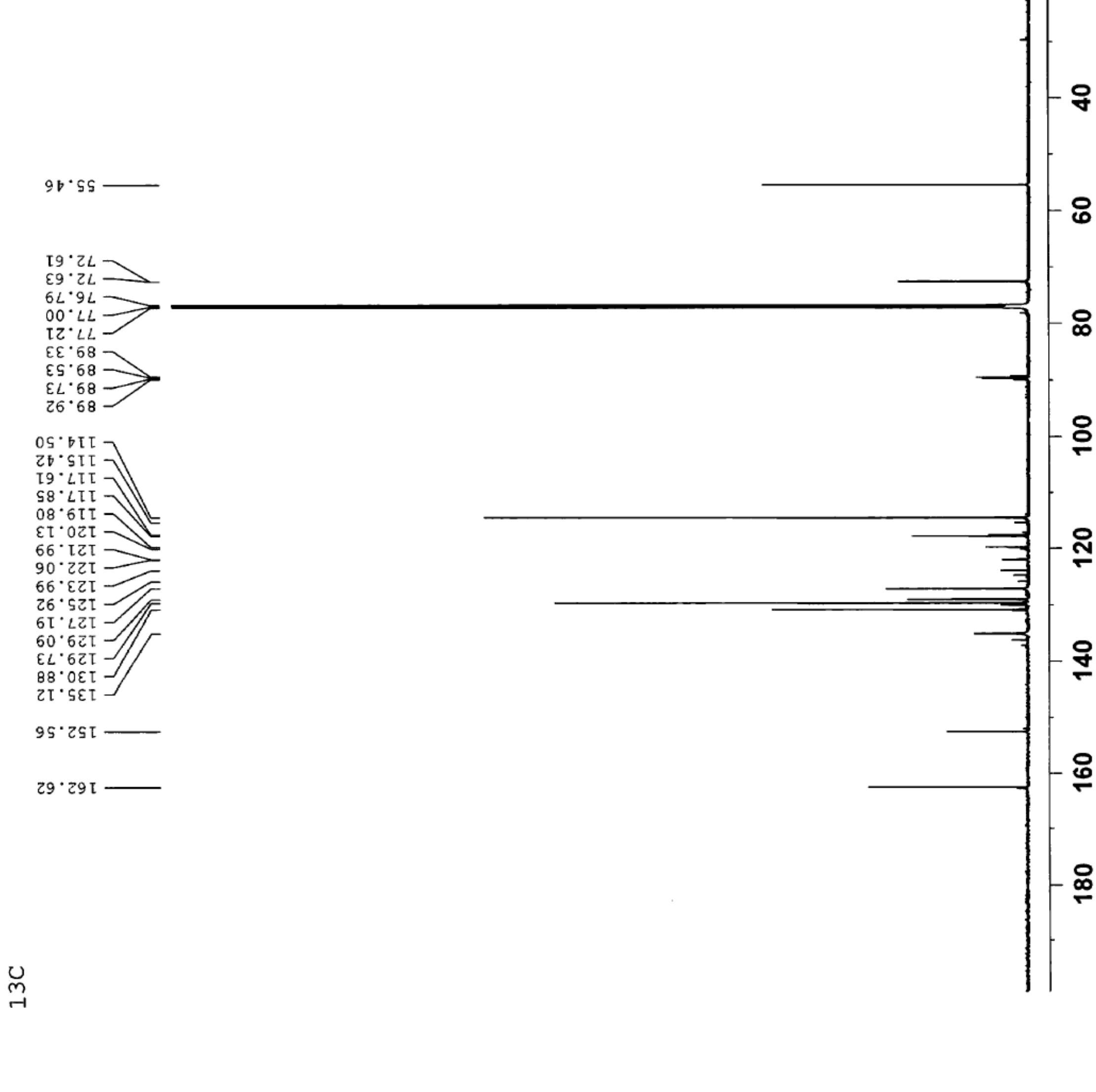

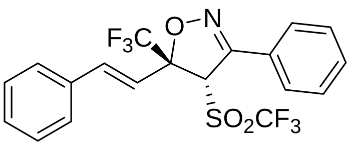

(4S*,5R*)-**1q**
(dr = 100:0)
^{1}H NMR

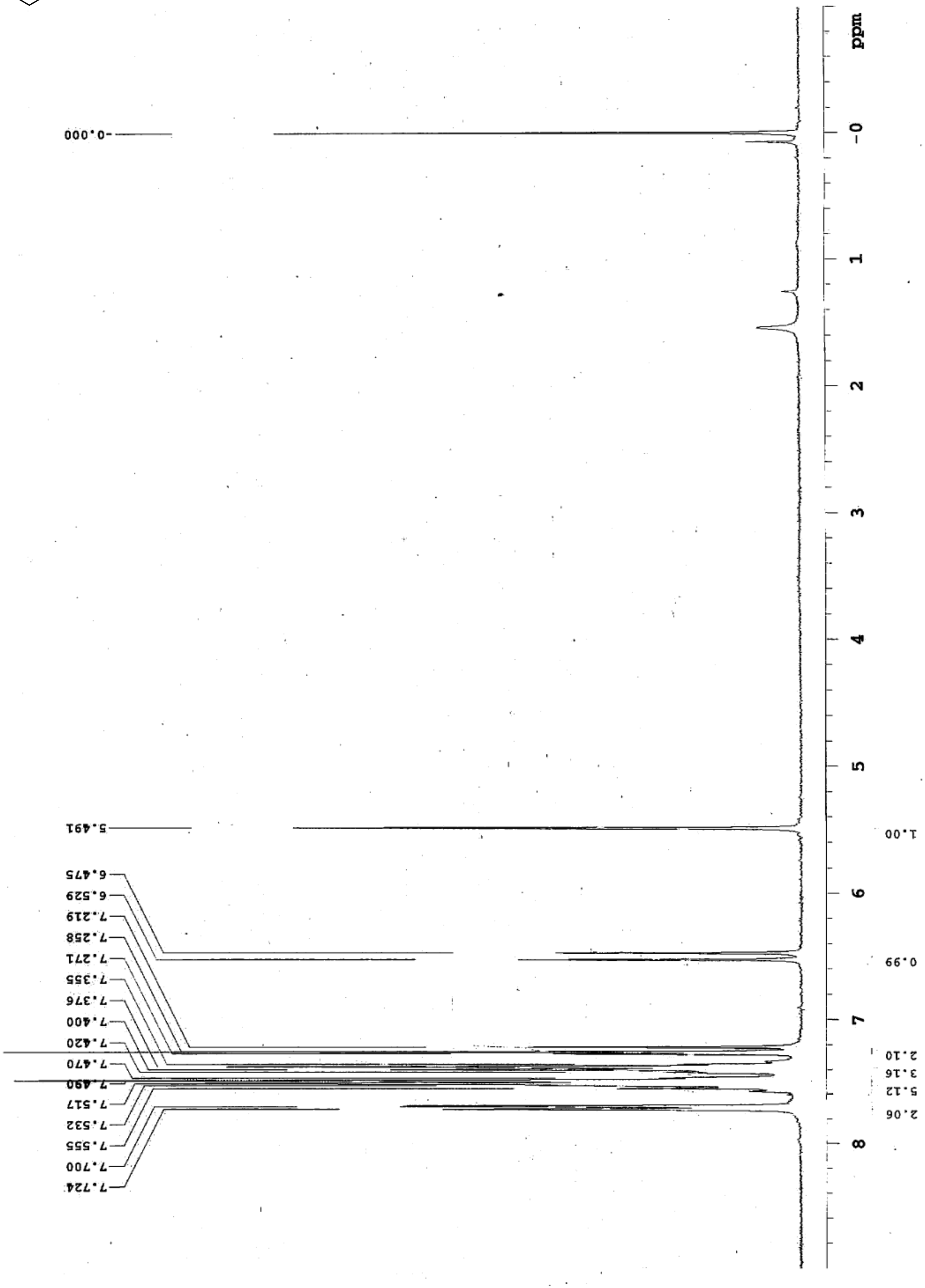

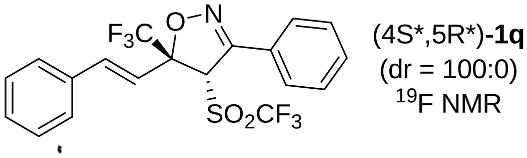

(4S*,5R*)-**1q**
(dr = 100:0)
^{19}F NMR

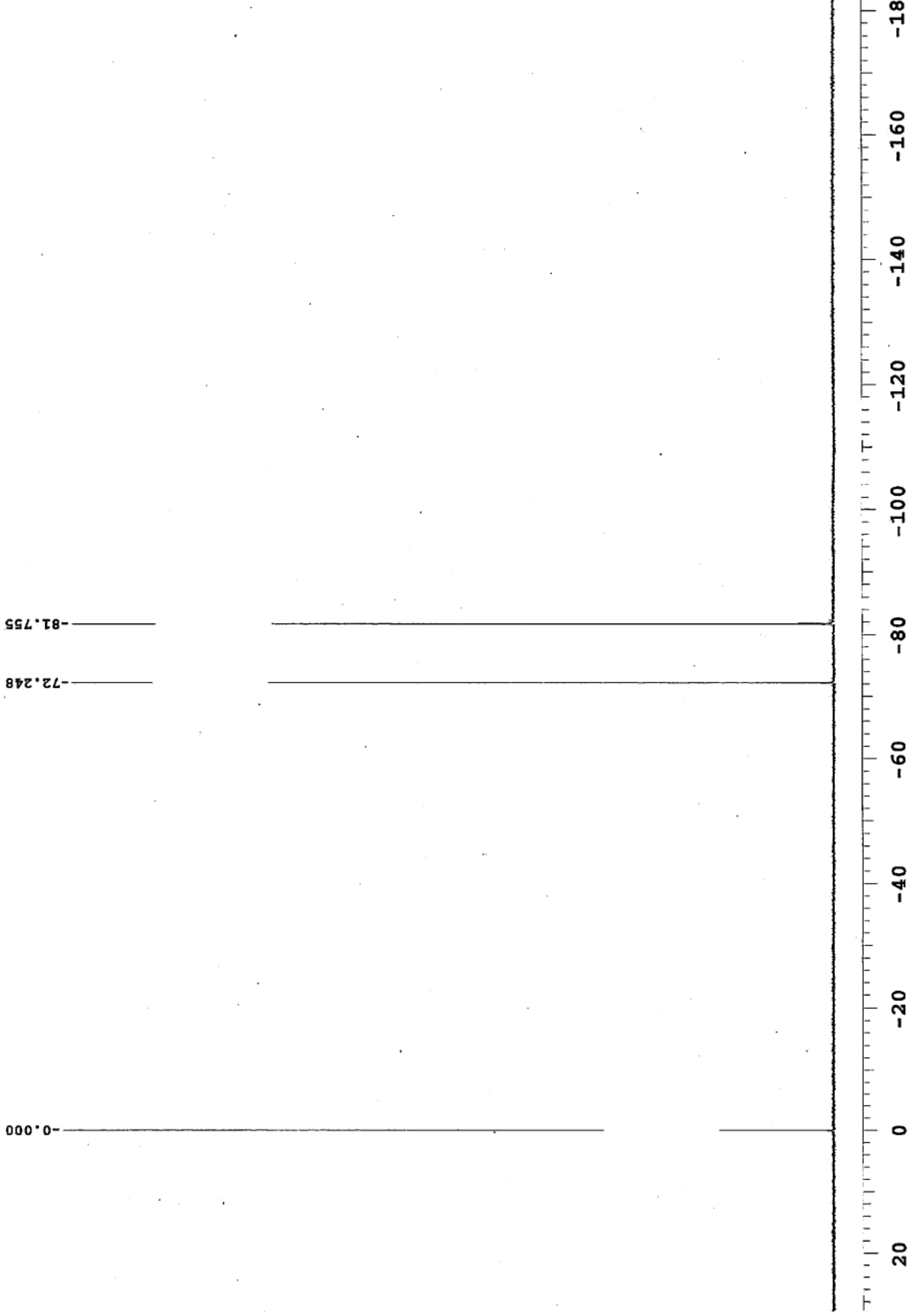

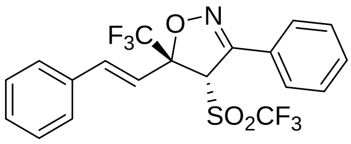

(4S*,5R*)-**1q**
(dr = 100:0)
^{13}C NMR

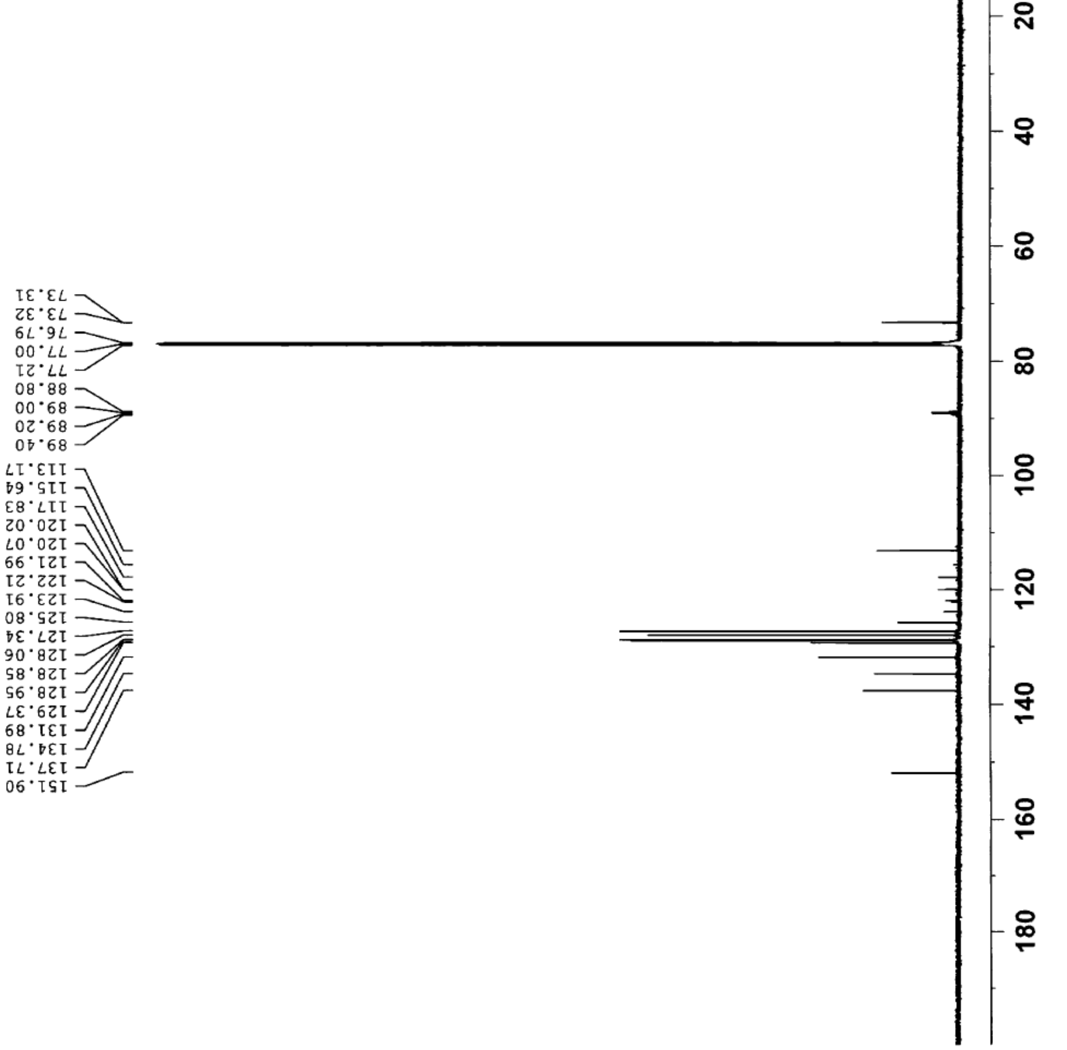

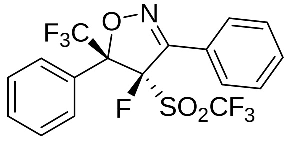

(4S*,5R*)-**1a-F**
(major isomer)
^{1}H NMR

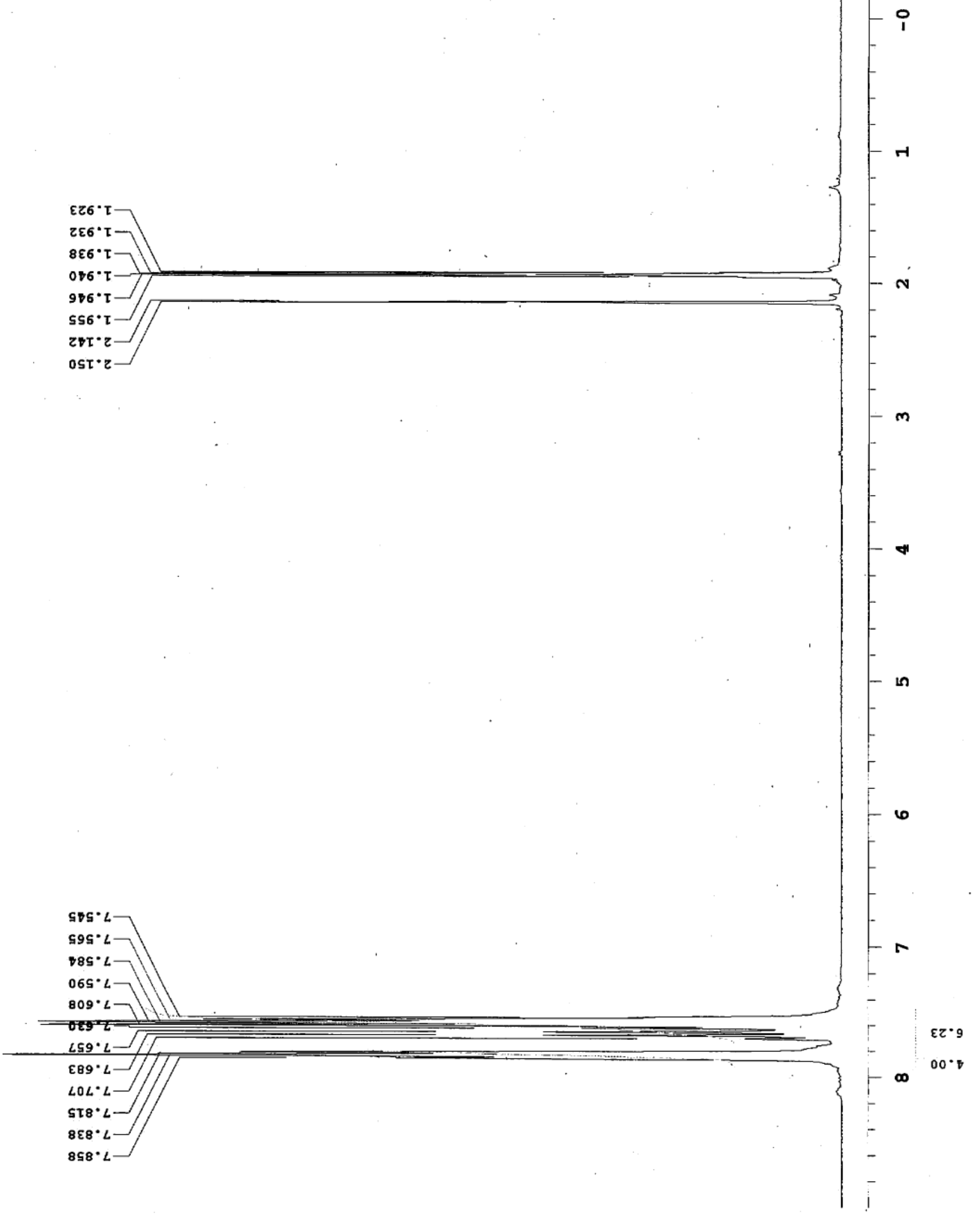

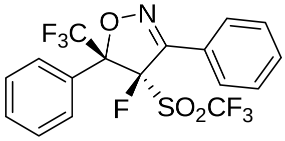

(4S*,5R*)-**1a-F**
(major isomer)
^{19}F NMR

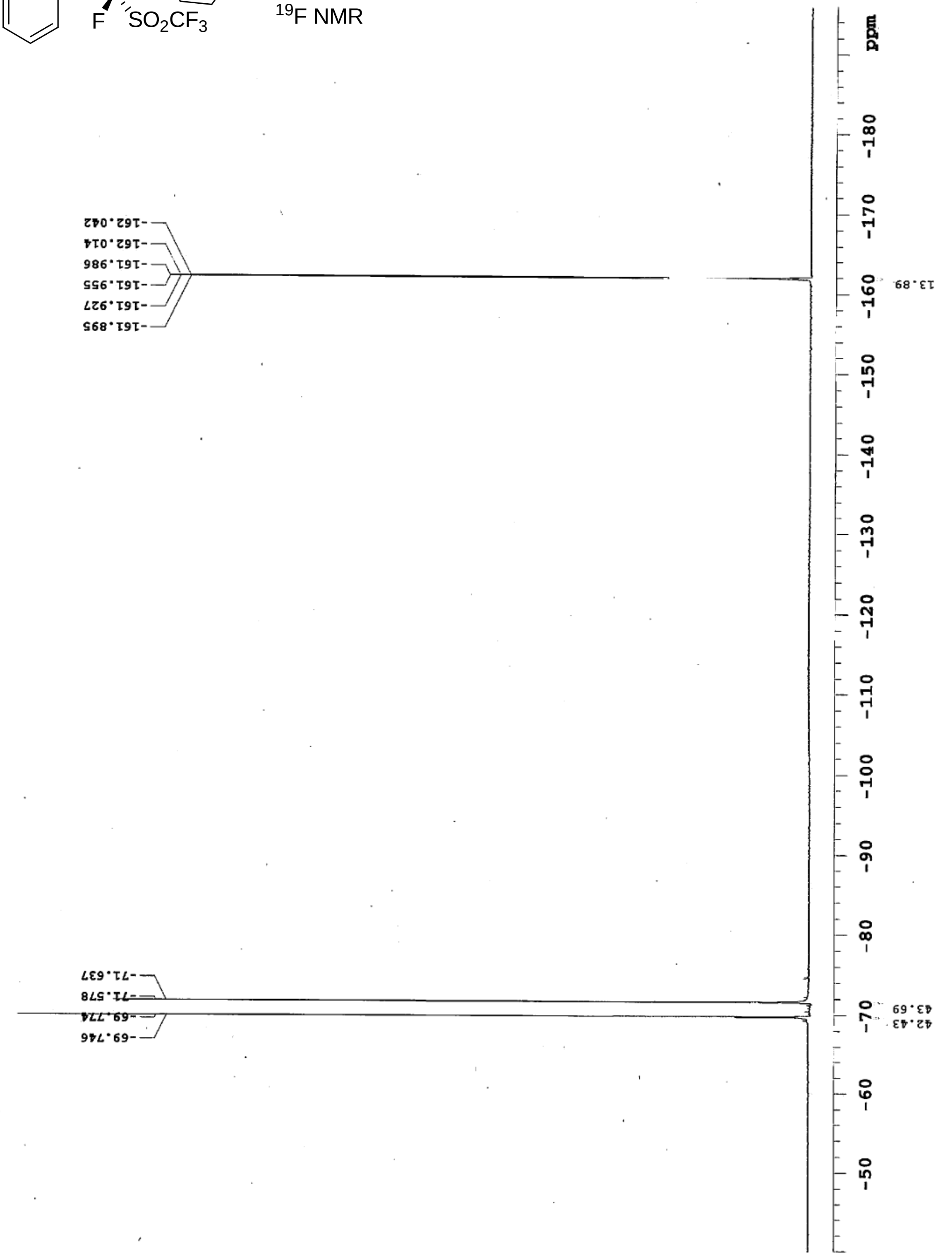

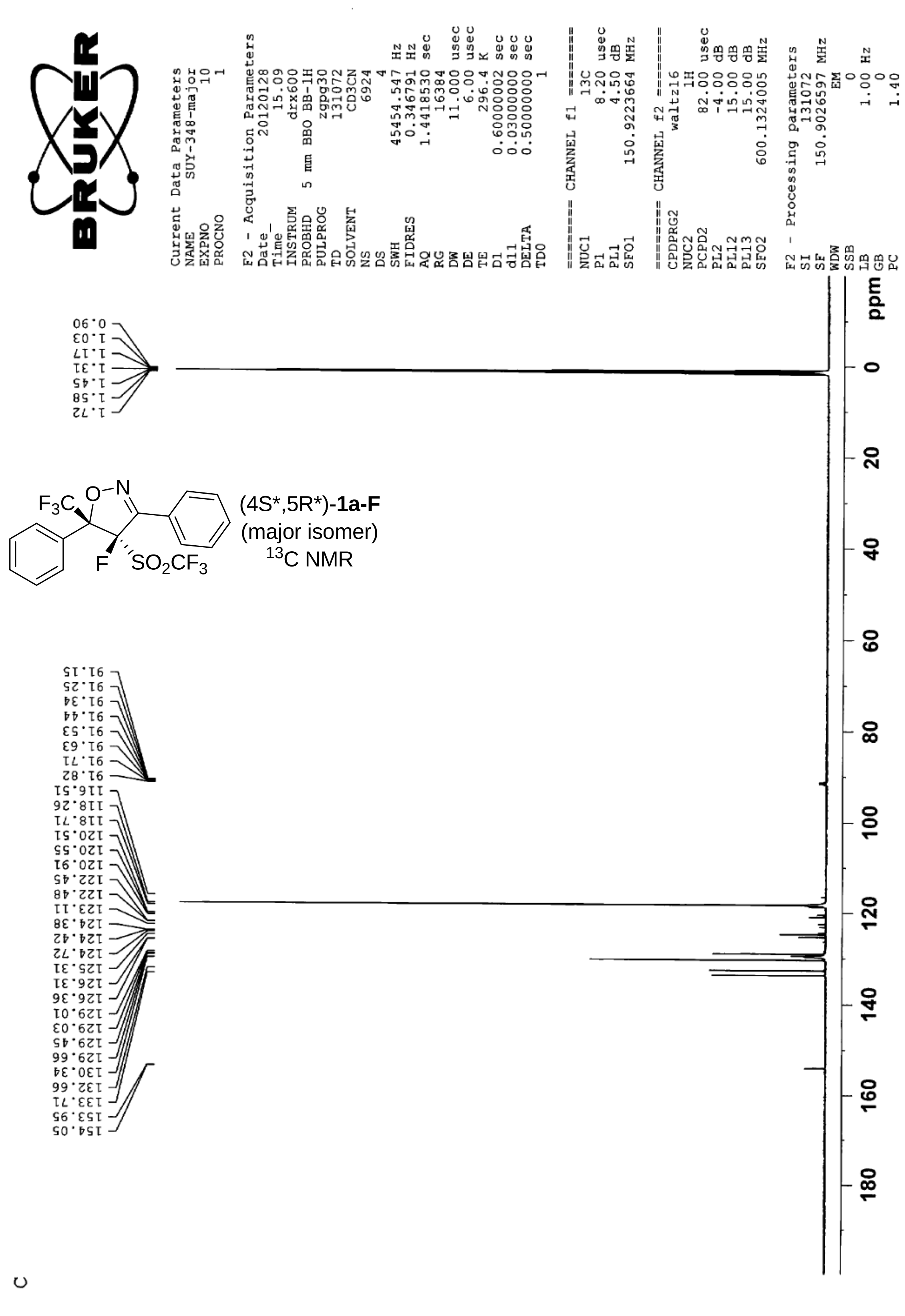

(4S*,5R*)-1a-F
(major isomer)
13C NMR
0.90
1.03
1.17
1.31
1.45
1.58
1.72
91.15
91.25
91.34
91.44
91.53
91.63
91.71
91.82
116.51
118.26
118.71
120.51
120.55
120.91
122.45
122.48
123.11
124.38
124.42
124.72
125.31
126.31
126.36
129.01
129.03
129.45
129.66
130.34
132.66
133.71
153.95
154.05
Current Data Parameters
NAME SUY-348-major
EXPNO 10
PROCNO 1
F2 - Acquisition Parameters
Date_ 20120128
Time 15.09
INSTRUM drx600
PROBHD 5 mm BBO BB-1H
PULPROG zgpg30
TD 131072
SOLVENT CD3CN
NS 6924
DS 4
SWH 45454.547 Hz
FIDRES 0.346791 Hz
AQ 1.4418530 sec
RG 16384
DW 11.000 usec
DE 6.00 usec
TE 296.4 K
D1 0.60000002 sec
d11 0.03000000 sec
DELTA 0.50000000 sec
TD0 1
======== CHANNEL f1 ========
NUC1 13C
P1 8.20 usec
PL1 4.50 dB
SFO1 150.9223664 MHz
======== CHANNEL f2 ========
CPDPRG2 waltz16
NUC2 1H
PCPD2 82.00 usec
PL2 -4.00 dB
PL12 15.00 dB
PL13 15.00 dB
SFO2 600.1324005 MHz
F2 - Processing parameters
SI 131072
SF 150.9026597 MHz
WDW EM
SSB 0
LB 1.00 Hz
GB 0
PC 1.40
ppm
0
20
40
60
80
100
120
140
160
180

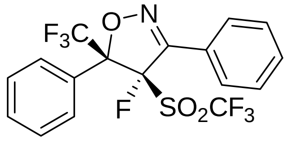

(4S*,5S*)-**1a-F**
(minor isomer)
^{1}H NMR

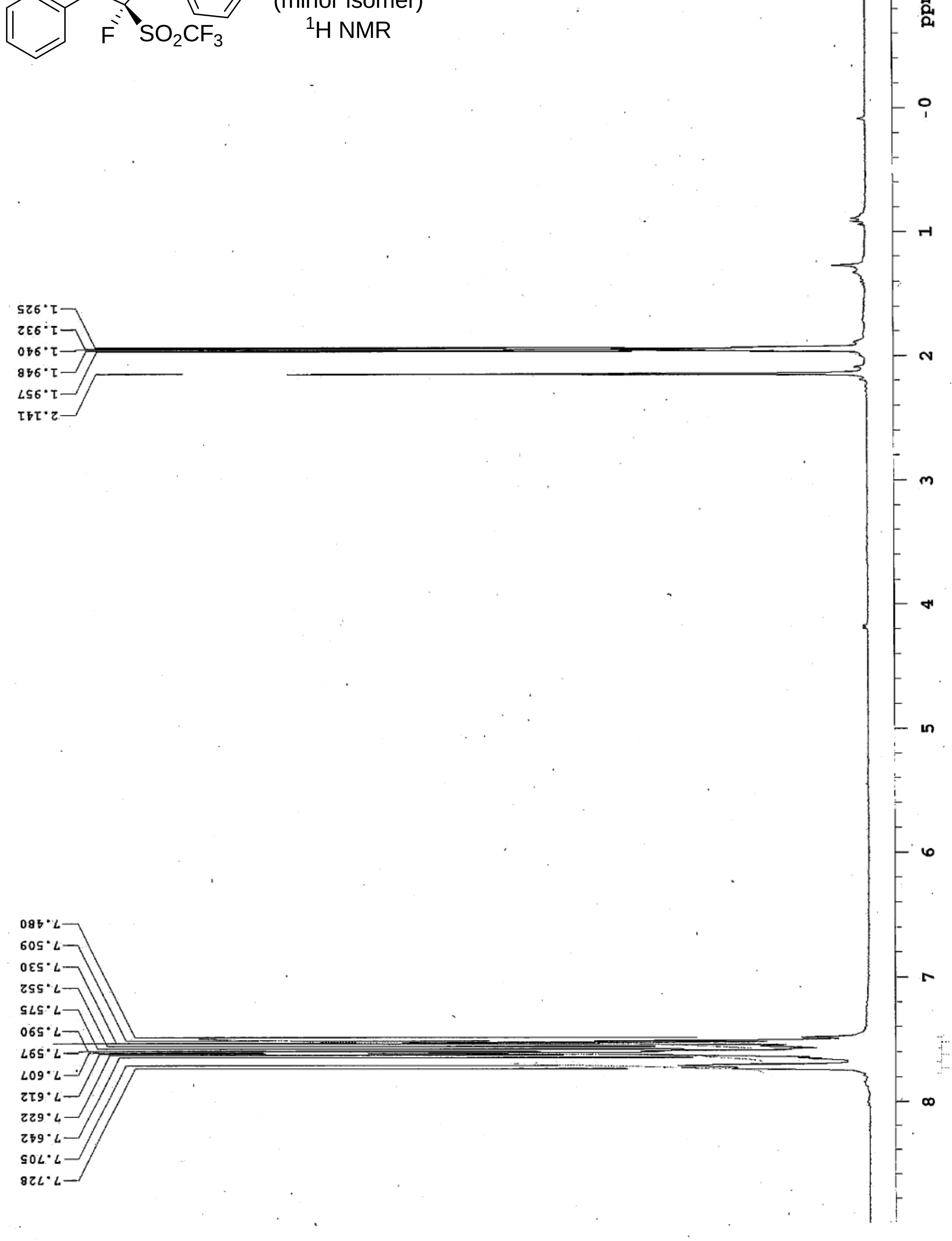

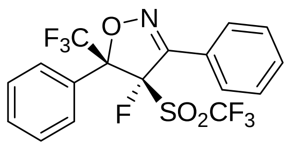

(4S*,5S*)-**1a-F**
(minor isomer)
^{19}F NMR

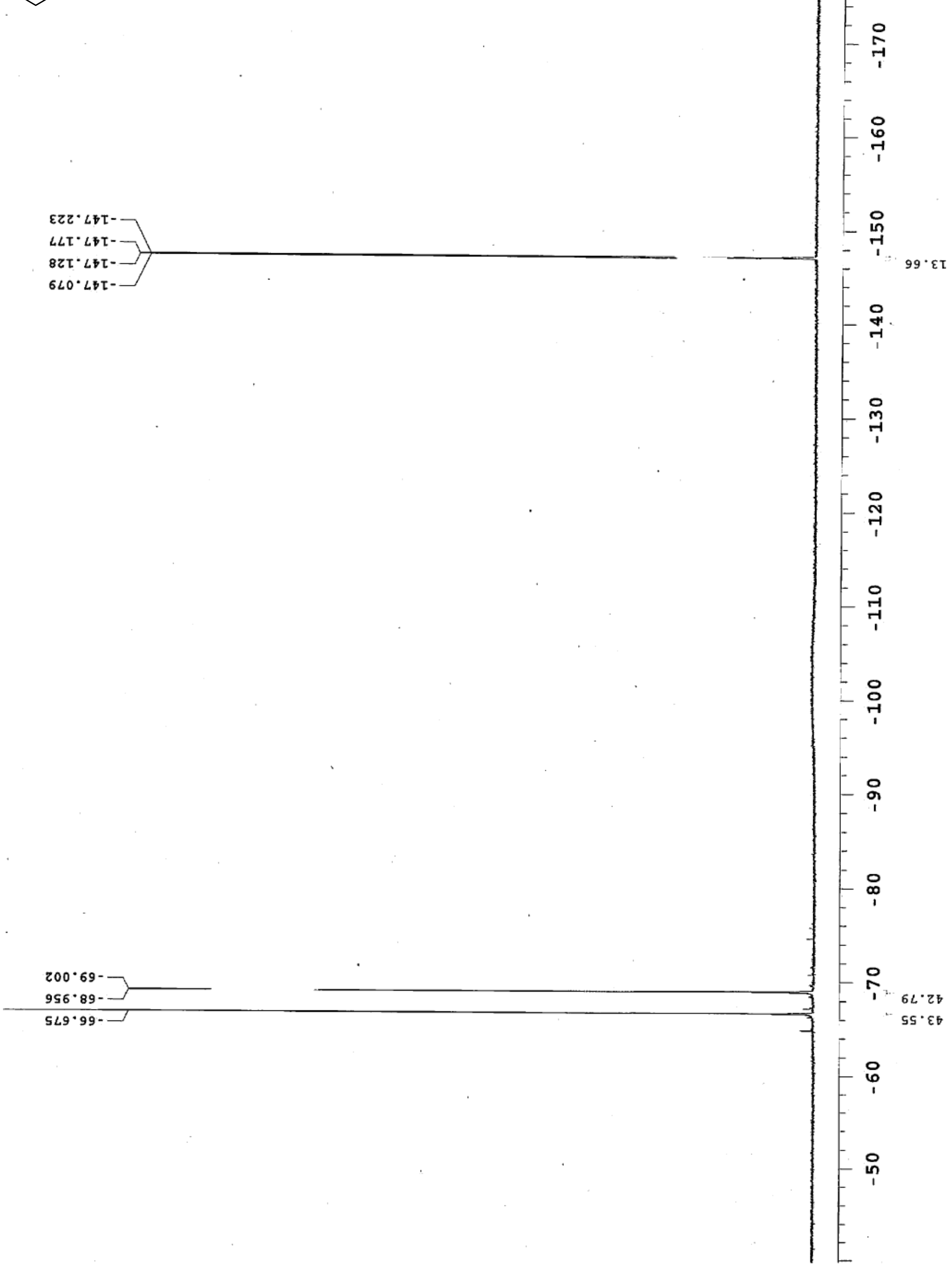

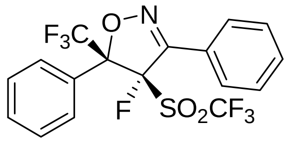

(4S*,5S*)-**1a-F**
(minor isomer)
^{13}C NMR

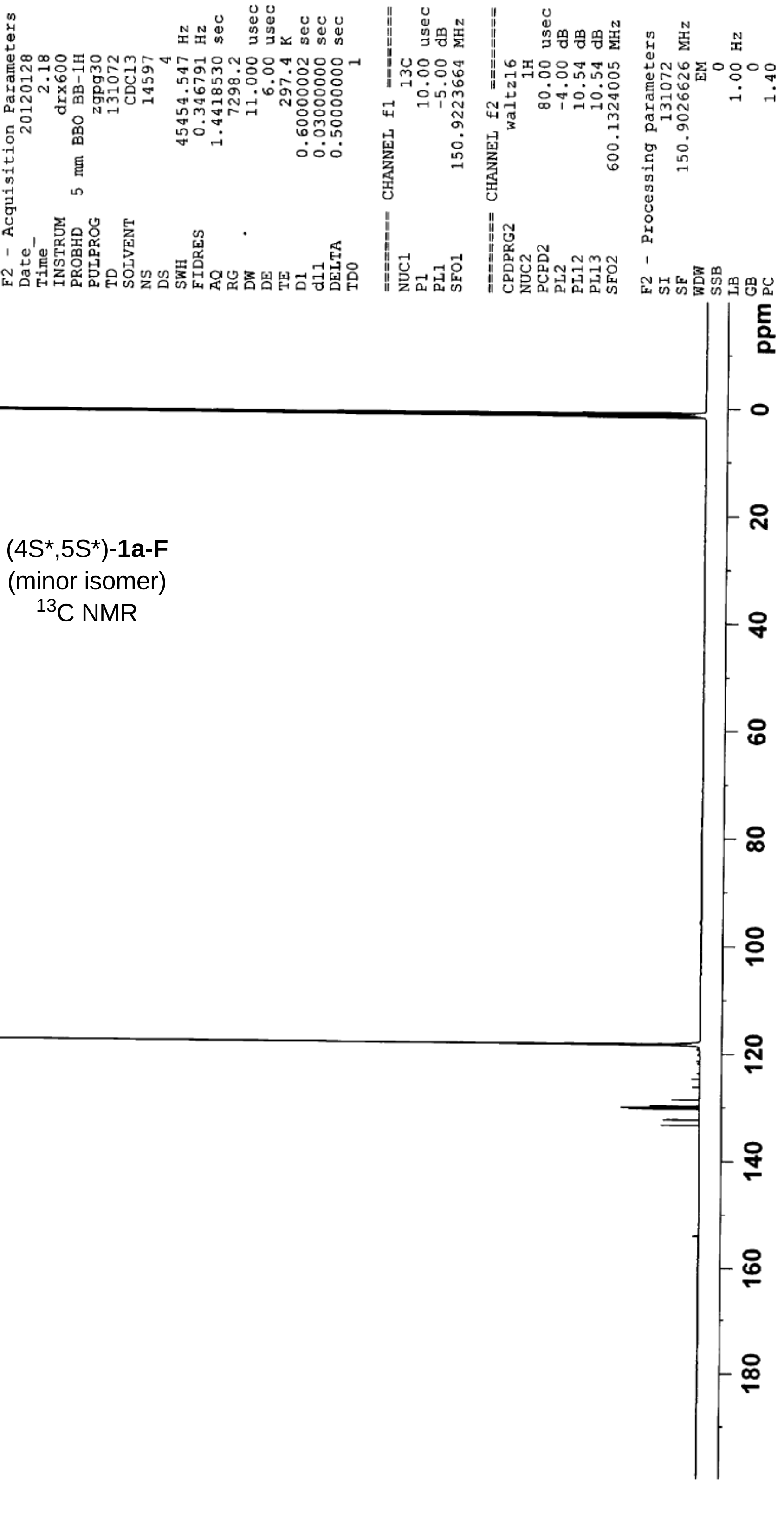

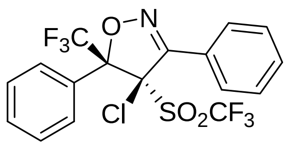

(4R*,5R*)-**1a-Cl**
(major isomer)
^{1}H NMR

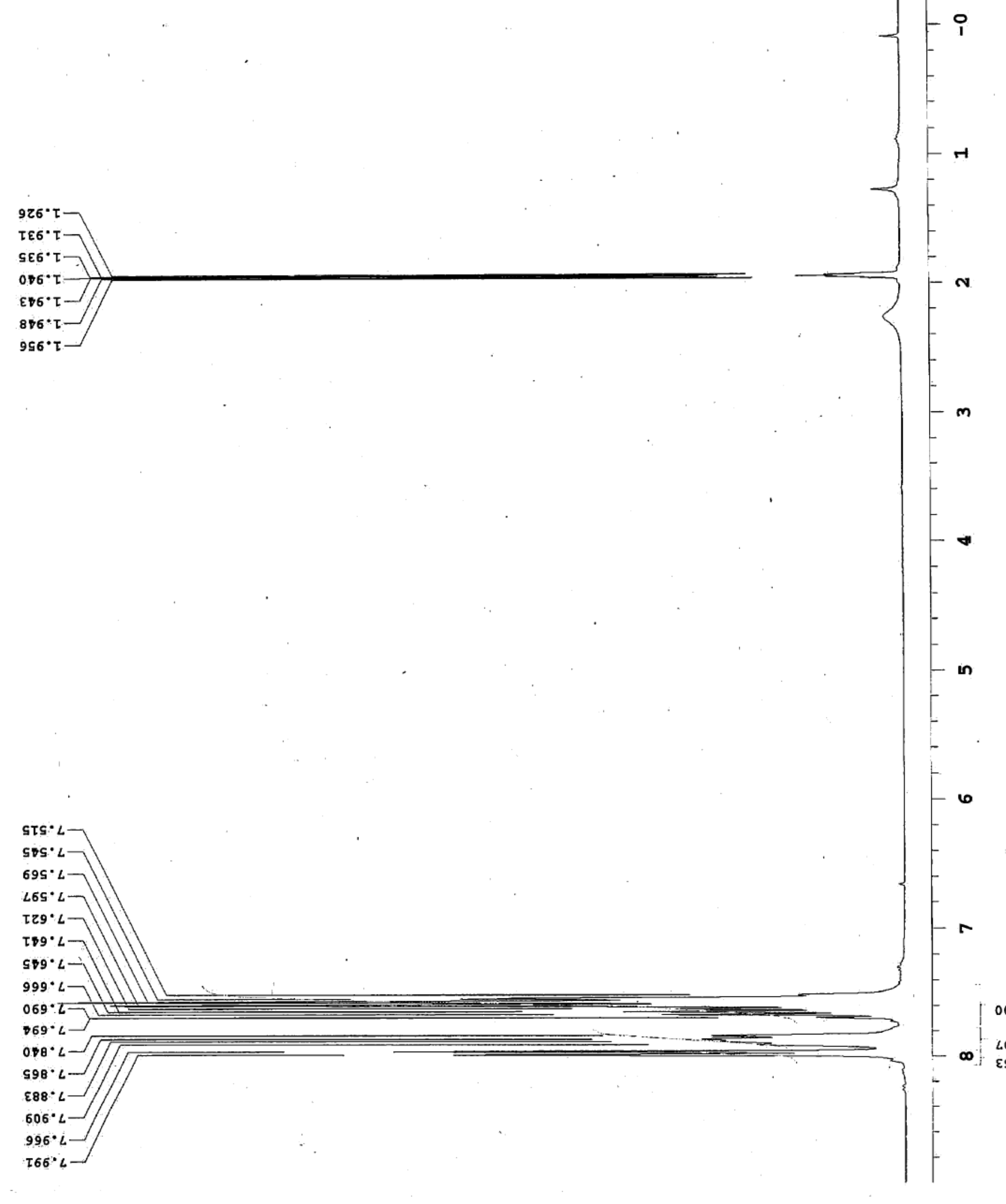

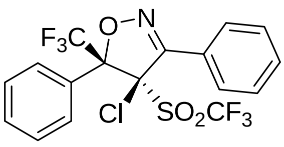

(4R*,5R*)-**1a-Cl**
(major isomer)
^{19}F NMR

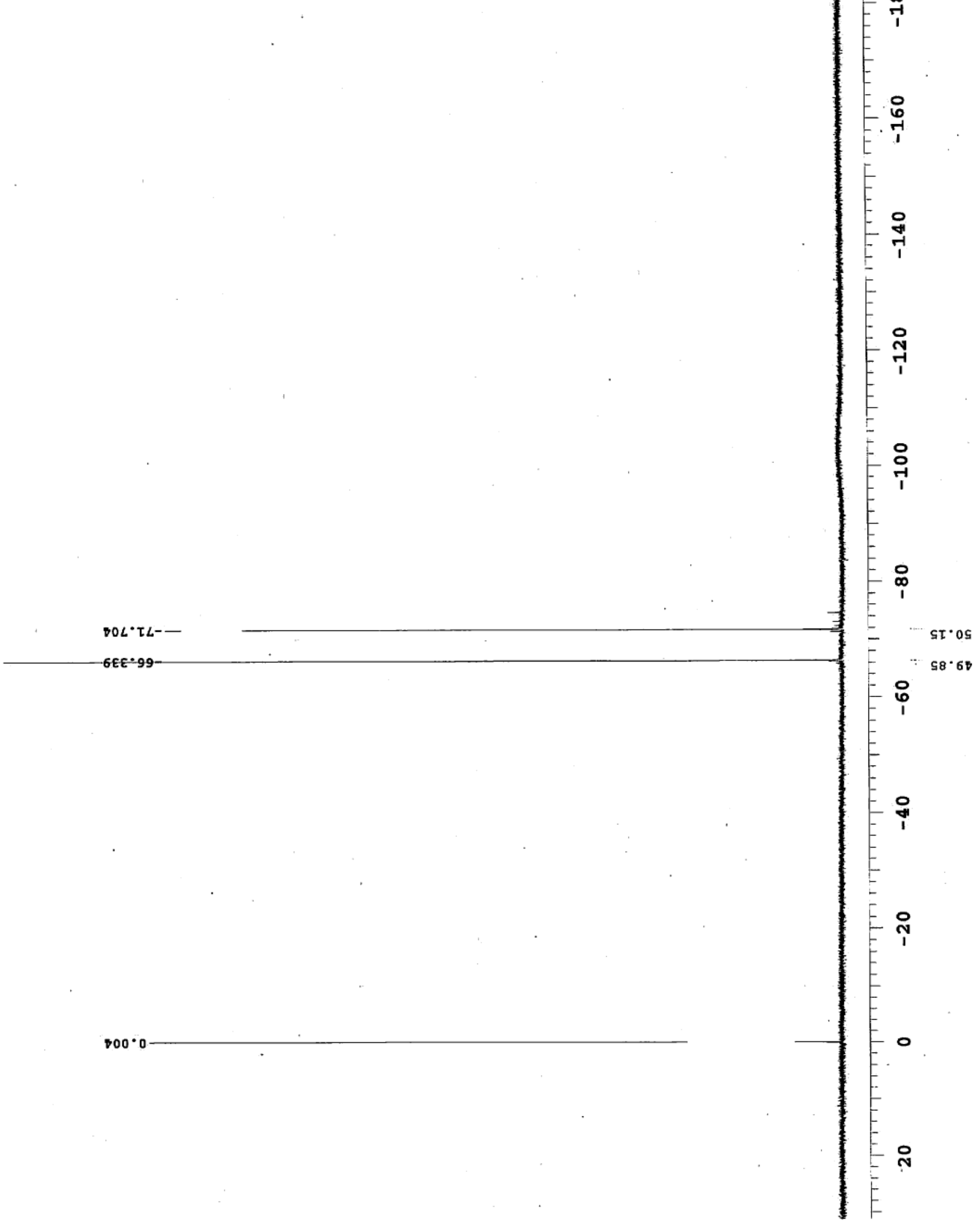

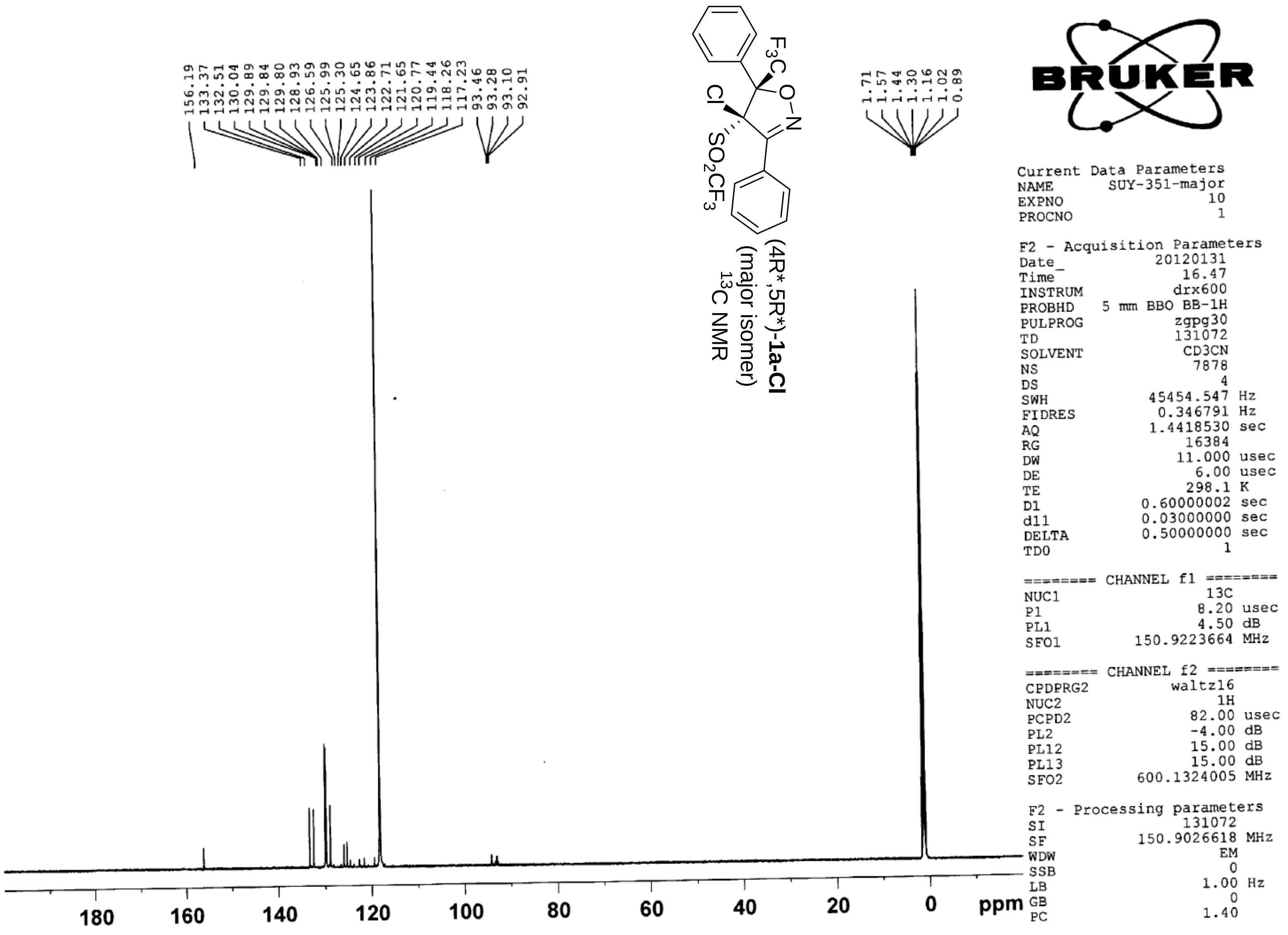
(4R*,5R*)-1a-Cl
(major isomer)
13C NMR
156.19
133.37
132.51
130.04
129.89
129.84
129.80
128.93
126.59
125.99
125.30
124.65
123.86
122.71
121.65
120.77
119.44
118.26
117.23
93.46
93.28
93.10
92.91
1.71
1.57
1.44
1.30
1.16
1.02
0.89
BRUKER
Current Data Parameters
NAME SUY-351-major
EXPNO 10
PROCNO 1
F2 - Acquisition Parameters
Date_ 20120131
Time_ 16.47
INSTRUM drx600
PROBHD 5 mm BBO BB-1H
PULPROG zgpg30
TD 131072
SOLVENT CD3CN
NS 7878
DS 4
SWH 45454.547 Hz
FIDRES 0.346791 Hz
AQ 1.4418530 sec
RG 16384
DW 11.000 usec
DE 6.00 usec
TE 298.1 K
D1 0.60000002 sec
d11 0.03000000 sec
DELTA 0.50000000 sec
TD0 1
======== CHANNEL f1 ========
NUC1 13C
P1 8.20 usec
PL1 4.50 dB
SFO1 150.9223664 MHz
======== CHANNEL f2 ========
CPDPRG2 waltz16
NUC2 1H
PCPD2 82.00 usec
PL2 -4.00 dB
PL12 15.00 dB
PL13 15.00 dB
SFO2 600.1324005 MHz
F2 - Processing parameters
SI 131072
SF 150.9026618 MHz
WDW EM
SSB 0
LB 1.00 Hz
GB 0
PC 1.40
180 160 140 120 100 80 60 40 20 0 ppm

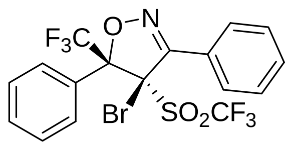

(4R*,5R*)-**1a-Br**
(dr = 95:5)
^{1}H NMR

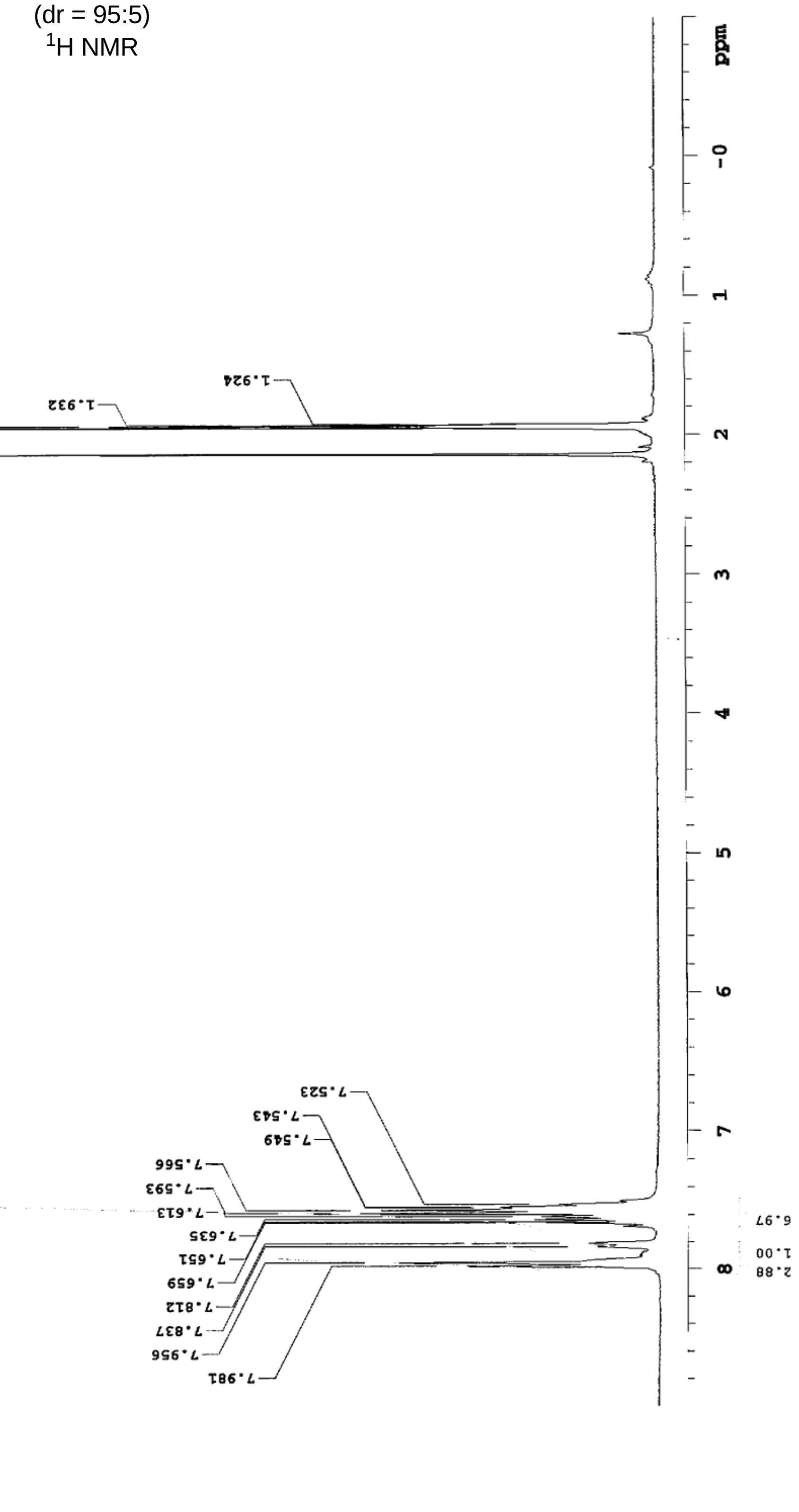

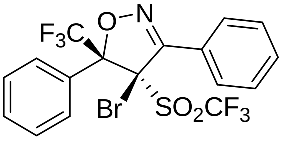

(4R*,5R*)-**1a-Br**
(dr = 95:5)
^{19}F NMR

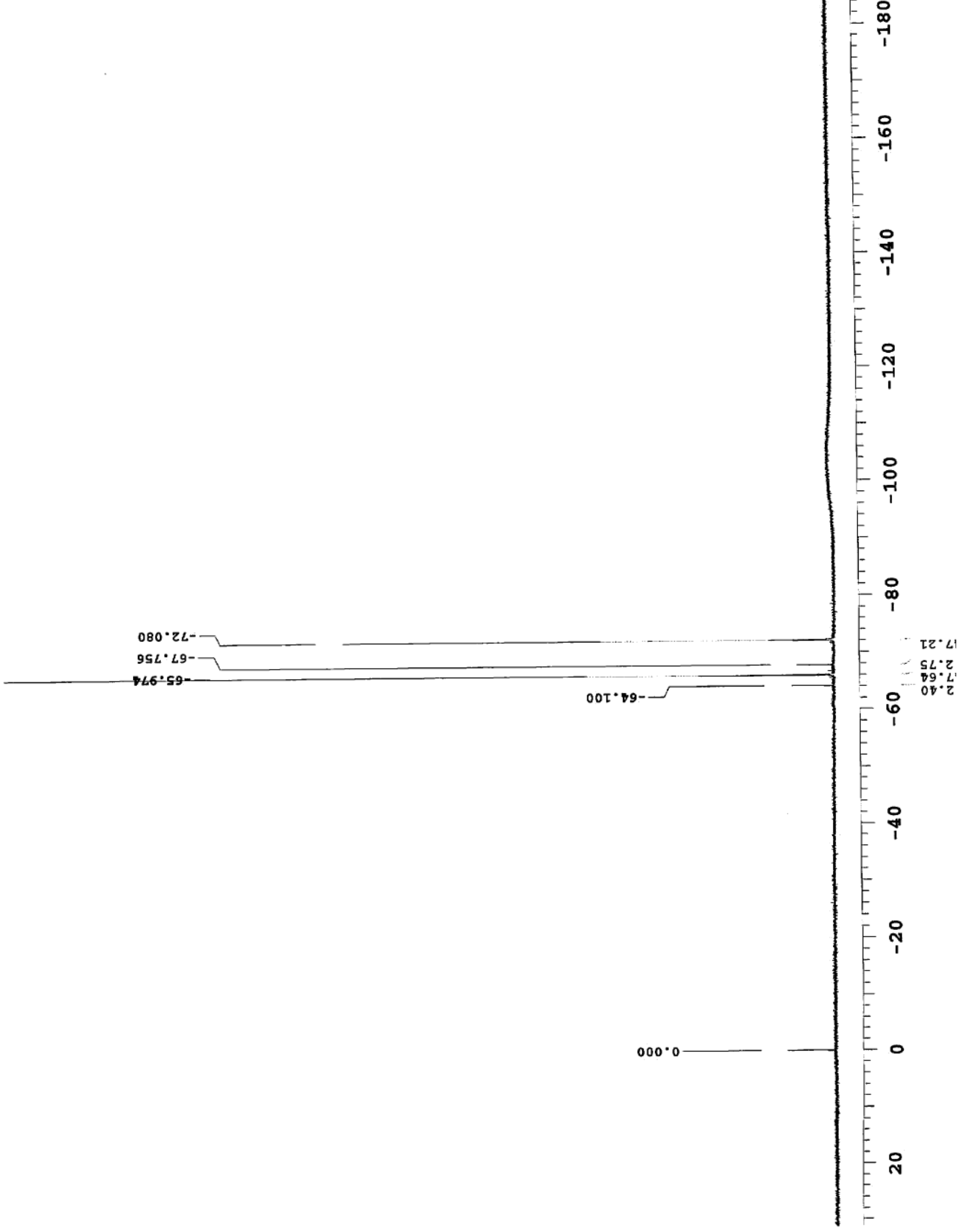

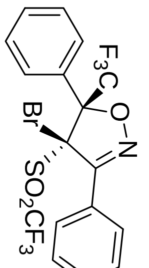

(4R*,5R*)-**1a-Br**
(dr = 95:5)
^{13}C NMR

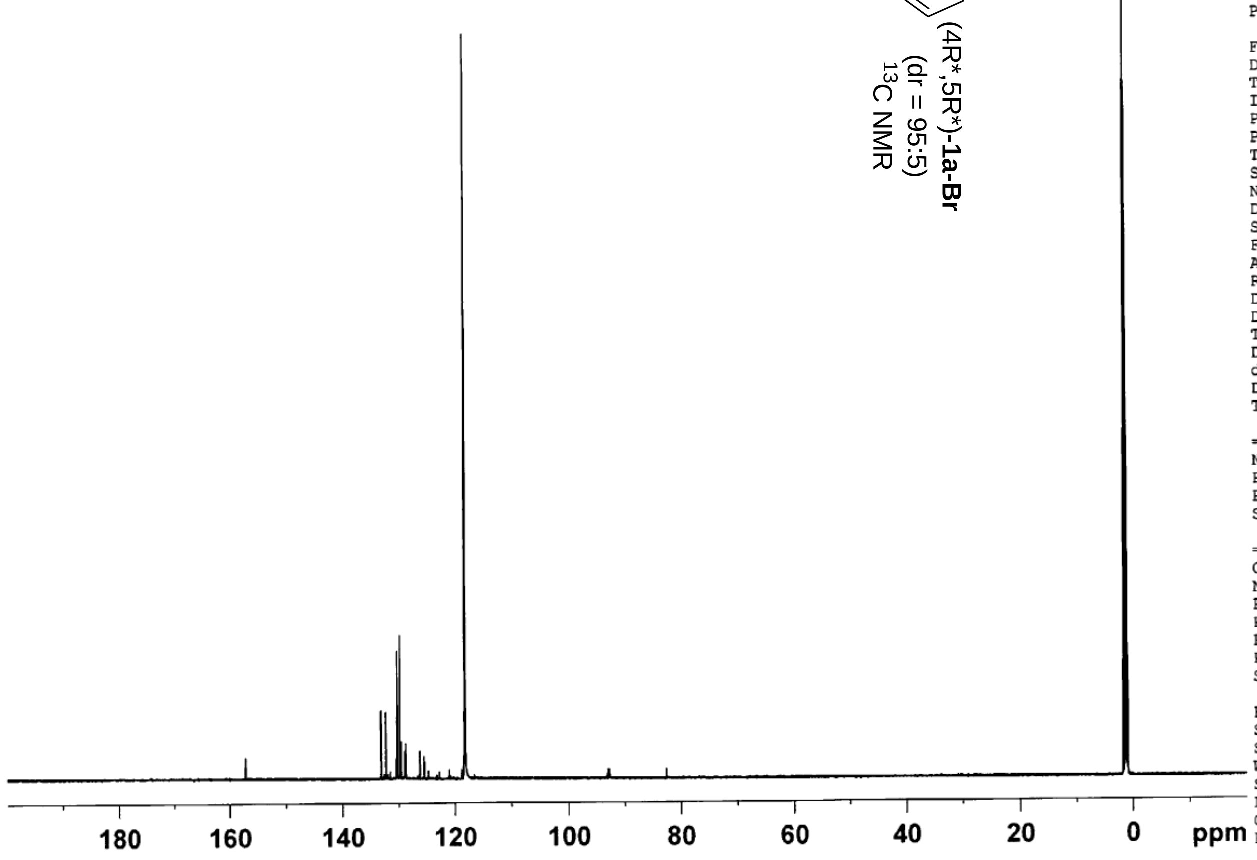


```
Current Data Parameters
NAME              SUY-365
EXPNO                  10
PROCNO                  1

F2 - Acquisition Parameters
Date_            20120203
Time                21.21
INSTRUM            drx600
PROBHD    5 mm BBO BB-1H
PULPROG           zgpg30
TD                 131072
SOLVENT             CD3CN
NS                   2824
DS                      4
SWH             45454.547 Hz
FIDRES           0.346791 Hz
AQ              1.4418530 sec
RG                   6502
DW                 11.000 usec
DE                   6.00 usec
TE                  296.3 K
D1             0.60000002 sec
d11            0.03000000 sec
DELTA          0.50000000 sec
TD0                     1

======== CHANNEL f1 ========
NUC1                  13C
P1                   8.20 usec
PL1                  4.50 dB
SFO1          150.9223664 MHz

======== CHANNEL f2 ========
CPDPRG2           waltz16
NUC2                   1H
PCPD2               82.00 usec
PL2                 -4.00 dB
PL12                15.00 dB
PL13                15.00 dB
SFO2         600.1324005 MHz

F2 - Processing parameters
SI                 131072
SF           150.9026656 MHz
WDW                    EM
SSB                     0
LB                   1.00 Hz
GB                      0
PC                   1.40
```
